# Peer-facilitated community-based interventions for adolescent health in low- and middle-income countries: A systematic review

**DOI:** 10.1371/journal.pone.0210468

**Published:** 2019-01-23

**Authors:** Kelly Rose-Clarke, Abigail Bentley, Cicely Marston, Audrey Prost

**Affiliations:** 1 Department of Global Health and Social Medicine, King’s College London, London, United Kingdom; 2 Institute for Global Health, University College London, London, United Kingdom; 3 Faculty of Public Health and Policy, London School of Hygiene and Tropical Medicine, London, United Kingdom; Aga Khan University, PAKISTAN

## Abstract

**Background:**

Adolescents aged 10–19 represent one sixth of the world’s population and have a high burden of morbidity, particularly in low-resource settings. We know little about the potential of community-based peer facilitators to improve adolescent health in such contexts.

**Methods:**

We did a systematic review of peer-facilitated community-based interventions for adolescent health in low- and middle-income countries (LMICs). We searched databases for randomised controlled trials of interventions featuring peer education, counselling, activism, and/or outreach facilitated by young people aged 10–24. We included trials with outcomes across key areas of adolescent health: infectious and vaccine preventable diseases, undernutrition, HIV/AIDS, sexual and reproductive health, unintentional injuries, violence, physical disorders, mental disorders and substance use. We summarised evidence from these trials narratively. PROSPERO registration: CRD42016039190.

**Results:**

We found 20 studies (61,014 adolescents). Fourteen studies tested interventions linked to schools or colleges, and 12 had non-peer-facilitated components, e.g. health worker training. Four studies had HIV-related outcomes, but none reported reductions in HIV prevalence or incidence. Nine studies had clinical sexual and reproductive health outcomes, but only one reported a positive effect: a reduction in Herpes Simplex Virus-2 incidence. Three studies had violence-related outcomes, two of which reported reductions in physical violence by school staff and perpetration of physical violence by adolescents. Seven studies had mental health outcomes, four of which reported reductions in depressive symptoms. Finally, we found eight studies on substance use, four of which reported reductions in alcohol consumption and smoking or tobacco use. There were no studies on infectious and vaccine preventable diseases, undernutrition, or injuries.

**Conclusions:**

There are few trials on the effects of peer-facilitated community-based interventions for adolescent health in LMICs. Existing trials have mixed results, with the most promising evidence supporting work with peer facilitators to improve adolescent mental health and reduce substance use and violence.

## Introduction

Adolescents (persons aged 10–19 years) constitute one sixth of the world’s population [1, 2]. Every year, 1.2 million die from preventable causes including road injury, self-harm, drowning, and interpersonal violence [3]. The burden of communicable diseases (HIV/AIDS, TB and malaria) is disproportionately high in this age group, and many non-communicable diseases in adulthood can be attributed to risk behaviours adopted during adolescence [2, 4, 5].

Global systematic reviews have found moderate- to high-quality evidence that interventions in communities and schools have positive effects on adolescent sexual and reproductive health, mental health, substance use, and intimate partner violence [6–10]. In several Low- and Middle-Income Countries (LMICs), peer facilitators, defined as adolescents or young adults selected from the group or community they serve, are employed to work in communities and schools as part of national and non-governmental adolescent health programmes. [11–14]. There are several reasons for this. Training lay peer facilitators to deliver adolescent health interventions can increase capacity for scaling up and be more cost-effective than working with specialised staff [15–17]. Peer facilitators may also be better able to communicate with adolescents than older adults, and perceived as a more credible source of information [18, 19]. Peer facilitators might have better access to marginalised groups who have limited engagement with existing health programmes [15, 20]. Critically, empowering young people to inform and implement adolescent health programmes should make these more relevant and effective [2]. The selection, training, supervision and incentivisation of peer facilitators are all deemed critical to success and sustainability [21].

Primary studies and reviews on the effects of peer-facilitated community interventions for adolescent health in LMICs have largely focused on sexual and reproductive health [15, 22–25]. No existing systematic review has examined evidence for the effects of peer-facilitated interventions across multiple areas of adolescent health in LMICs, despite the fact that community interventions are likely to rely on the same human resources for many areas of adolescent health. To address this gap, we conducted a systematic review of community-based peer-facilitated interventions in LMICs for the key areas of adolescent health defined by the Lancet Commission on Adolescent Health and Wellbeing: infectious and vaccine preventable diseases, undernutrition, HIV and AIDS, sexual and reproductive health, unintentional injuries, violence, physical disorders, mental disorders and substance use [2].

## Methods

We conducted the systematic review in accordance with the 2009 PRISMA statement (S1 Checklist) [26].

### Inclusion criteria for the systematic review

We only included randomised controlled trials (RCTs) because these studies have a lower risk of bias compared to quasi-experimental studies. We included trials in which the majority (>50%) of participants were adolescents or participants with a mean or median age of 10–19. Trials had to be located in the community (e.g. schools, youth clubs or primary health care centres) because this is where peer-facilitated interventions are commonly located. Trials also had to take place in LMICs (as defined by the World Bank [27]), and test an intervention delivered in whole or part by peer facilitators, defined here as persons or a majority of persons (>50%) with a mean or median age of 10–24 recruited from the group or community meant to benefit from interventions. We included trials of interventions involving peer education where peers sought to increase adolescents’ knowledge or influence their attitudes, ‘counselling’, defined as peers providing support to help adolescents resolve personal or psychological problems, ‘activism’ involving peer-led campaigns to change health-related policy, and ‘outreach’ with peers engaging marginalised adolescents [28, 29]. We included trials with primary or secondary outcomes relevant to areas of health need outlined in the report of the Lancet Commission on Adolescent Health and Wellbeing [2]: infectious and vaccine preventable diseases, undernutrition, HIV and AIDS, sexual and reproductive health, unintentional injuries, violence, physical disorders, mental disorders and substance use. We deliberately included interventions from across multiple adolescent health areas in order to compare effects across areas. For each area of health need, we included studies with outcomes related to the diseases and risk factors highlighted by the Lancet Commission Report, as well as diseases constituting the 10 main global causes of death or years lived with disability for 10–19 year olds [2, 4]. These outcomes are shown in Table 1. We also included educational and employment marginalisation, which were considered key determinants of adolescent health. We did not include studies that were conducted in underprivileged populations in high-income countries. No date or language restrictions were applied. The review protocol is registered with PROSPERO (CRD42016039190). Our methods did not deviate from those specified in the protocol.

**Table 1:** Outcomes included in the review by area of health need.

Area of health need/determinant	Condition*	Outcome measures included in the review
Infectious and vaccine preventable diseases	TB Malaria Hepatitis B Measles Rubella Diphtheria-tetanus Influenza Meningitis Diarrhoeal diseases Intestinal infectious diseases Lower respiratory tract infections Skin and subcutaneous infections	Clinical outcomes: serum/sputum /faecal/urine tests, biopsy, clinical assessment by a trained health worker Self-reported symptoms: e.g. of diarrhoea
Undernutrition	Underweight Stunting Wasting Iron deficiency anaemia	Clinical outcomes: anthropometric and serum tests
HIV and AIDS		Clinical outcomes: serum test
Sexual and reproductive health	Sexually transmitted disease (syphilis, herpes, gonorrhoea, trichomoniasis, chlamydia, human papilloma virus) Adolescent births Early marriage Met needs for contraception Maternal death	Clinical outcomes: serum/urine/swab test, clinical assessment by a trained health worker Self-reported symptoms/outcomes: STD symptoms, pregnancy, marriage Intermediate behavioural outcomes: condom use
Unintentional injuries	Road injuries Drowning Burns Exposure to mechanical forces	Clinical outcomes: clinical assessment/records Self reported symptoms/outcomes: exposure/injury
Violence	Physical, emotional or sexual violence	Self-reported symptoms/outcomes: exposure or perpetration of violence
Physical disorders	Overweight and obesity Haemoglobinopathies and haemolytic anaemias Congenital anomalies Ischaemic heart disease Skin and subcutaneous disorders (eczema, acne, psoriasis) Low back and neck pain Asthma Sense organ diseases (refractive errors) Migraine	Clinical outcomes: anthropometric, serum test, clinical assessment by a trained health worker, biopsy Self reported symptoms: symptoms e.g. migraine or asthma symptoms
Mental health disorders	Depressive disorders Anxiety disorders Autistic spectrum disorder Conduct disorder Suicide Self-harm	Clinical outcomes: clinical assessment Self reported symptoms: mental health screening tools
Substance use	Risky alcohol use Tobacco use Drug use disorders	Clinical outcomes: clinical assessment, serum or urine test Self-reported symptoms: screening tools, reported substance use
Educational and employment marginalisation	Education completion, School attendance Educational intentions	Self-reported outcomes: attendance and intentions

* For each area of health need we included studies with outcomes related to the diseases and risk factors highlighted by the Lancet Commission Report, as well as diseases constituting the 10 main global causes of death or years lived with disability for 10–19 year olds [2, 4].

### Search strategy

**Fig 1:**
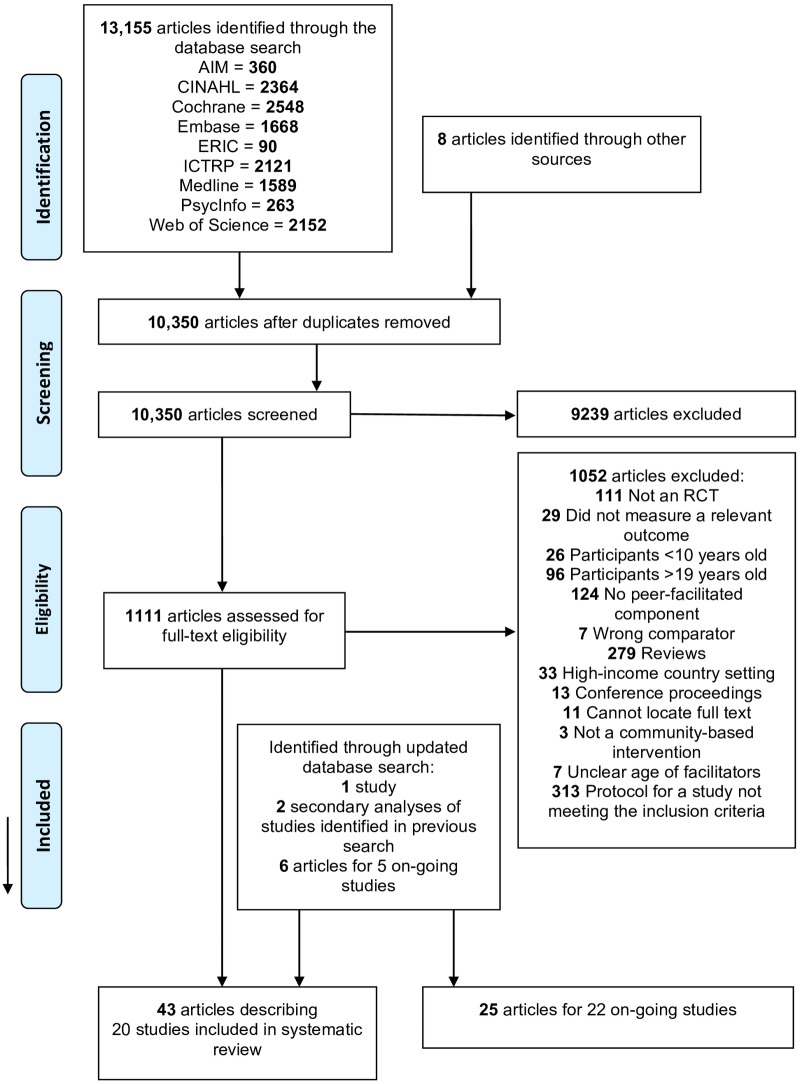
PRISMA 2009 Flow Diagram. *From*: Moher D, Liberati A, Tetzlaff J, Altman DG, The PRISMA Group (2009). *P*referred *R*eporting *I*tems for *S*ystematic Reviews and *M*eta-*A*nalyses: The PRISMA Statement. PLoS Med 6(7): e1000097. doi: 10.1371/journal.pmed1000097 **For more information, visit** www.prisma-statement.org.

KR-C used customised search strategies (S1 Text) to search for studies that met the inclusion criteria in Medline, Embase, Cochrane Library, CINAHL, African Index Medicus, Web of Science, Psycinfo and ERIC up to 9^th^ March 2017. The search was later updated to 22^nd^ June 2018. We identified ongoing studies by contacting adolescent health experts and searching the International Clinical Trials Registry Platform. We found further studies by searching relevant reviews. Fig 1 summarises the study selection process. KR-C or AB screened the title and abstract of each article to identify and exclude those that were irrelevant. KR-C and AB or AP then independently screened the full text of all remaining articles for relevance. Any discrepancies were discussed and resolved by the review team and/or by contacting authors. S2 Text outlines reasons for excluding articles at full text screening. S1 Table describes details of ongoing studies. We used Covidence and EndNote reference manager software to manage articles retrieved by the search [30].

For each study that met the inclusion criteria, KR-C and AB or AP independently extracted data on general study details, trial design, participant characteristics, sample size, intervention, control condition, outcomes and summary measures, for example a risk ratio (RR), odds ratio (OR), or linear regression coefficient (β). We noted whether interventions involved education, counselling, activism and/or outreach strategies. We extracted data from the first outcome assessment post-intervention based on a hierarchy of clinical outcomes first (e.g. HSV-2 serum test), then outcomes related to self-reported symptoms (e.g. STD symptoms), and finally behavioural outcomes (e.g. condom use). We did not exclude studies on the basis of methodological quality, but used the Cochrane Collaboration’s Risk of Bias Tool to assess studies across the following bias domains: sequence generation, allocation concealment, participants and personnel blinding, outcome assessment blinding, incomplete outcome data, selective outcome reporting and other bias [31].

### Data synthesis

We mapped the evidence using a narrative summary of intervention characteristics by area of health need. Within each area of health need, we also considered how complementary intervention activities, setting, type of facilitator and participant age could influence intervention effects. Although we initially planned to do a statistical meta-analysis, this was not possible because of the wide variation in types of interventions and outcomes.

## Results

We found 43 articles that described 20 relevant randomised controlled trials with a total of 61,014 participants at baseline. S2 Table summarises the characteristics of these studies. Six were conducted in low-income countries, seven in lower-middle income countries and seven in upper middle-income countries. Fourteen interventions were linked to schools or a college. Twelve interventions had additional non-peer-facilitated components, for example health worker or teacher training, and dissemination of educational materials. These other non-peer-facilitated components are described in Table 2.

**Table 2:** Characteristics of peer-facilitated components of adolescent health interventions and intervention effects.

First author and year of publication of main trial paper	*Peer facilitation only* or *multi-component* intervention	Strategy	Description of peer-facilitated component	Delivery method	Total duration (weeks)	Frequency and no. peer-facilitated sessions	Description of peer facilitators	Selection of peer facilitators	Training of peer facilitators	Supervision of peer facilitators	Incentives for peer facilitators	Outcome measure	Effect	P value
**HIV AND AIDS**														
Cowan 2010	Multi-component	Education	The youth programme for in- and out-of-school youth is delivered by carefully selected and trained Zimbabwean school leavers in the year between leaving school and starting university. These school leavers work as volunteers and go to live and work in the rural communities for 8–10 months of the year. They act both as role models for young people and as a bridge between adults and youth within communities. These professional peer educators (PPEs) use well structured, theoretically based materials, which they deliver in a highly participatory way. PPEs also help run ‘youth corners’ at clinics and help facilitate sessions in the parents programme	Groups, individual meetings	208	?	School leavers in the year between leaving school and starting university	?	?	?	?	HIV prevalence (male)	OR 1.2 (0.66, 2.18)	>0.05
												HIV prevalence (female)	OR 1.15 (0.81, 1.64)	>0.05
Jewkes 2008	Peer facilitation only	Education	Peer-facilitated group education sessions involving roleplay and drama based on participants’ lived experiences. Groups are single sex for the first 13 sessions then there are three meetings where males and females come together, and a community meeting at the end of the program. Group sessions cover topics such as sex and love, conception and contraception, unwanted pregnancy and sexually transmitted diseases and HIV.	Groups	6–8 weeks	16*3 hour sessions	Males and females the same age or a little older than participants. Most had further education or had undergone life skills training	Peer facilitators were selected whose attitudes were supportive of gender equity and non-judgemental regarding sexuality	3 weeks of training and 2 practice groups	Research staff made ad hoc visits to workshops that were in progress. Facilitators were observed and any issues related to the workshops were discussed with them, however there was no attempt to micro-manage the progress of intervention delivery	?	HIV incidence	RR 0.95 (0.67, 1.35)	0.78
Ross 2007	Multi-component	Education, counselling, outreach	Condom promoters/distributers (CPDs): four to five youth per village selected by their peers promoted and distributed condoms. School-based reproductive health education: this was led by teachers, but "selected pupils called class peer educators (CPE), were given a role in performing carefully scripted dramas which aimed to demonstrate desired behaviours and to emphasize the importance and relevance of key messages. Trainers of Peers: three male and three female youth were selected to act as trainers of peers (TOPs) and assisted in the training of CPE and other community activities. TOPs also acted as sources of information in their community.	Presentation, individual meetings	156	?	Primary school students	CPEs = Co-selected by teachers, research team and peers; CPDs = selected by peers; TOPs = selected by adults in their community	CPEs were trained by the TOPS. CPDs were trained for two days.	Teachers supervised CPEs. Research staff and TOPs supervised CPDs.	Salaries were provided for TOPS but after start up the TOPS training role devolved to teachers. CPEs and CPDs did not receive salaries	HIV incidence (female)	RR 0.75 (0.34, 1.66)	>0.05
Sherman 2009	Peer facilitation only	Education, outreach	Peer education using a curriculum of group sessions to teach participants to think critically about and reduce their methamphetamine use and sexual risk behaviours. "Participants were taught communication skills that they practiced in role plays during the sessions and used to convey methamphetamine and risk reduction messages to specific social network members that were identified through a social network inventory administered at baseline."	Groups, individual meetings	4	?	Current drug users aged 18–25 years	?	Peer facilitators were trained by the researchers in an intensive week-long training. Index participants received 7 education sessions	?	Index participants were compensated 200 Baht ($5 USD) for each of the five study assessments and each of the seven intervention and control sessions, resulting in the opportunity to earn a total of $55 USD.	HIV incidence	Control rate (per 100 PY) 0; Intervention rate 0.96	>0.05
**SEXUAL AND REPRODUCTIVE HEALTH**														
Balaji 2010	Multi-component	Education	Peer leaders were given a resource guide to help them to conduct group sessions and perform street plays to other youth in their communities in order to communicate information about intervention target issues. In each village, some youths were also trained to socially market condoms to other youth.	Groups, presentation	52	?	?	Selected by the research team	Trained by psychologists and social workers experienced in the field of adolescent health	Rural peer leaders were supported by a Community Advisory Board comprising of key people such as village council leaders. Urban peer leaders were supported by trained teachers and integrated into existing student forums	"Moderate" monetary and other incentives (certificates) provided	Complaints of penile discharge (rural)	0.89 (0.61, 1.30)	0.55
												Complaints of vaginal symptoms (rural)	OR 0.92 (0.26, 3.24)	0.9
												Complaints of penile discharge (urban)	OR 0.36 (0.24, 0.55)	<0.001
												Complaints of vaginal symptoms (urban)	OR 0.49 (0.26, 0.93)	0.03
Cowan 2010	Multi-component	Education	The youth programme for in- and out-of-school youth is delivered by carefully selected and trained Zimbabwean school leavers in the year between leaving school and starting university. These school leavers work as volunteers and go to live and work in the rural communities for 8–10 months of the year. They act both as role models for young people and as a bridge between adults and youth within communities. These professional peer educators (PPEs) use well structured, theoretically based materials, which they deliver in a highly participatory way. PPEs also help run ‘youth corners’ at clinics and help facilitate sessions in the parents programme	Groups, individual meetings	208	?	School leavers in the year between leaving school and starting university	?	?	?	?	HSV-2 infection (male)	OR 1.23 (0.69, 2.18)	>0.05
												HSV-2 infection (female)	OR 1.24 (0.93, 1.65)	>0.05
Decat 2015	Multi-component	Education, outreach, counselling, activism	Peer leaders ("Friends of Youth" FOYs) mentor adolescents in their communities to help them build competence in making deliberate choices, and to refer and accompany them to health care providers as necessary. FOYs also conduct family talks, facilitate mobile cinemas (films on SRH), distribute educational materials for parents of adolescents, workshops for parents, work with community leaders to provide opportunities for adolescents, maintain Facebook page, awareness raising/capacity building with health facilities, outreach to vulnerable adolescents to encourage them to go to healthcare centres, and work with the Ministry of Health	Groups, policy engagement, individual meetings	72	?	Youths aged 24 or younger living in the same community as study participants	?	?	Supervised by the programme implementers of the research team	Small financial incentives for FOYs	Improved condom use	β -2.66	0.039^a^
Jewkes 2008	Peer facilitation only	Education	Peer-facilitated group education sessions involving roleplay and drama based on participants’ lived experiences. Groups are single sex for the first 13 sessions then there are three meetings where males and females come together, and a community meeting at the end of the program. Group sessions cover topics such as sex and love, conception and contraception, unwanted pregnancy and sexually transmitted diseases and HIV.	Groups	6–8 weeks	16*3 hour sessions	Males and females the same age or a little older than participants. Most had further education or had undergone life skills training	Peer facilitators were selected whose attitudes were supportive of gender equity and non-judgemental regarding sexuality	3 weeks of training and 2 practice groups	Research staff made ad hoc visits to workshops that were in progress. Facilitators were observed and any issues related to the workshops were discussed with them, however there was no attempt to micro-manage the progress of intervention delivery	?	HSV-2 infection	RR 0.67 (0.47, 0.97)	0.036
Mmbaga 2017	Multi-component	Education	Nine peer-led lessons that were part of an after-school life skills training curriculum. Topics included decision-making skills, puberty and self-protection Sessions focused on experiential learning using narratives, role-play and drama.	Groups	9	Weekly 60–90 min sessions	?	?	?	Teachers were available in the lessons to offer support.	?	Condom use (male)	β 0.2173	0.004
												Condom use (female)	β 0.0162	0.463
Okonofua 2003	Multi-component	Education, counselling	Peer educators were trained in STD prevention and treatment to provide one-to-one or group counselling to other students, to distribute educational materials, and to refer adolescents with STD symptoms to trained health providers	Groups, individual meetings	44	?	Students aged 14–18 years	Selected by peers	Trained in school over 4 weeks on STD prevention and treatment including symptom recognition, benefits of early treatment, sources of treatment, prevention of STDs, need for partner notification and to defer sexual intercourse until treatment is complete. Training used standardised educational models.	?	?	Self-reported STD symptoms	OR 0.63 (0.43, 0.91)	<0.05
Ross 2007	Multi-component	Education, counselling, outreach	Condom promoters/distributers (CPDs): four to five youth per village selected by their peers promoted and distributed condoms. School-based reproductive health education: this was led by teachers, but "selected pupils called class peer educators (CPE), were given a role in performing carefully scripted dramas which aimed to demonstrate desired behaviours and to emphasize the importance and relevance of key messages. Trainers of Peers: three male and three female youth were selected to act as trainers of peers (TOPs) and assisted in the training of CPE and other community activities. TOPs also acted as sources of information in their community.	Presentation, individual meetings	156	?	Primary school students	CPEs = Co-selected by teachers, research team and peers; CPDs = selected by peers; TOPs = selected by adults in their community	CPEs were trained by the TOPS. CPDs were trained for two days.	Teachers supervised CPEs. Research staff and TOPs supervised CPDs.	Salaries were provided for TOPS but after start up the TOPS training role devolved to teachers. CPEs and CPDs did not receive salaries	HSV- infection (male)	RR 0.92 (0.69, 1.22)	>0.05
												Syphilis infection (male)	RR 0.78 (0.46, 1.30)	>0.05
												Chlamydia infection (male)	RR 1.14 (0.53, 2.43)	>0.05
												HSV-2 infection (female)	RR 1.05 (0.83, 1.32)	>0.05
												Syphillis infection (female)	RR 0.99 (0.67, 1.46)	>0.05
												Chlamydia (female)	RR 1.37 (0.98, 1.91)	>0.05
												Gonorrhoea (female)	RR 1.93 (1.01, 3.71)	<0.05 ^a^
												Trichomonas (female)	RR 1.13 (0.92, 1.37)	>0.05
												Pregnancy test (female)	RR 1.09 (0.85, 1.40)	>0.05
Sherman 2009	Peer facilitation only	Education, outreach	Peer education using a curriculum of group sessions to teach participants to think critically about and reduce their methamphetamine use and sexual risk behaviours. "Participants were taught communication skills that they practiced in role plays during the sessions and used to convey methamphetamine and risk reduction messages to specific social network members that were identified through a social network inventory administered at baseline."	Groups, individual meetings	4	?	Current drug users aged 18–25 years	?	Peer facilitators were trained by the researchers in an intensive week-long training. Index participants received 7 education sessions	?	Index participants were compensated 200 Baht ($5 USD) for each of the five study assessments and each of the seven intervention and control sessions, resulting in the opportunity to earn a total of $55 USD.	Chlamydia	Control rate 11.29; intervention rate 8.39	>0.05
												Gonorrhoea	Control rate 0.43; intervention rate 4.69	<0.05^a^
												HCV	Control rate 0.57; intervention rate 0	>0.05
												HSV-2	Control rate 2.93; intervention rate 4.09	>0.05
Thurman 2016	Peer facilitation only	Education, counselling	Facilitators led manualised interpersonal psychotherapy group (IPTG) sessions to help adolescents learn how to resolve distress and to access emotional support from group members. Groups were divided by gender. Facilitators also led a curriculum-based group behavioural intervention addressing HIV risk factors and pathways, covering alcohol, substance abuse, crime and sexual violence, HIV/AIDS, healthy sexual relationships, transactional sex and condom use. The intervention aimed to encourage social learning through reflection. Groups were mixed gender to encourage dialogue and understanding from different gender perspectives	Groups	IPTG = 16/Vhutshilo = 13	IPTG = weekly 90 min sessions/Vhutshilo = weekly 60 min sessions	High school graduates aged 23–25 years old with relevant prior experience e.g. coaching youth sports teams and teaching Sunday school	Selected by the research team	10 day training by the research team	Social workers provided supervision for facilitators	Monthly stipend of USD 230	Consistent condom use (male)	β 0.41 (SE -0.40)	0.31
												Consistent condom use (female)	β 1.21 (SE 0.52)	0.02
**VIOLENCE**														
Balaji 2010	Multi-component	Education	Peer leaders were given a resource guide to help them to conduct group sessions and perform street plays to other youth in their communities in order to communicate information about intervention target issues. In each village, some youths were also trained to socially market condoms to other youth.	Groups, presentation	52	?	?	Selected by the research team	Trained by psychologists and social workers experienced in the field of adolescent health	Rural peer leaders were supported by a Community Advisory Board comprising of key people such as village council leaders. Urban peer leaders were supported by trained teachers and integrated into existing student forums	"Moderate" monetary and other incentives (certificates) provided	Experience of physical abuse (rural)	OR 0.96 (0.49, 1.91)	0.92
												Experience of sexual abuse (rural)	OR 0.39 (0.12, 1.3)	0.12
												Perpetration of physical abuse (rural)	0.29 (0.15, 0.57)	<0.001
												Experience of physical abuse (urban)	OR 0.73 (0.42, 1.28)	0.27
												Experience of sexual abuse (urban)	OR 0.19 (0.09, 0.41)	<0.001
												Perpetration of physical abuse (urban)	OR 0.59 (0.40, 0.87)	0.01
Devries 2015	Multi-component	Education, counselling, activism	Students are selected to be members of the intervention-implementing committees in each school in order to contribute to decision-making and to be a role model for their peers. The intervention also involves students creating dramas and facilitating a ‘student court’ to handle school discipline issues.	Groups, presentation, policy engagement	76	?	Primary school students	Selected by peers or from existing student bodies	Members of the student court were trained by teacher ‘protagonists’ in positive discipline through role play and mock court sessions. Good school committee members were trained by Raising Voices staff and teachers using through manualised sessions	Supported by protagonist teachers and Raising Voices Staff	No financial incentive	Past week physical violence by school staff (reported by students)	OR 0.39 (0.25, 0.62)	<0.0001
												Past week physical violence by school staff (reported by school staff)	OR 0.37 (0.20, 0.69)	0.0018
												Past term physical violence by school staff (reported by students)	OR 0.31 (0.18, 0.53)	<0.0001
Jewkes 2008	Peer facilitation only	Education	Peer-facilitated group education sessions involving roleplay and drama based on participants’ lived experiences. Groups are single sex for the first 13 sessions then there are three meetings where males and females come together, and a community meeting at the end of the program. Group sessions cover topics such as sex and love, conception and contraception, unwanted pregnancy and sexually transmitted diseases and HIV.	Groups	6–8 weeks	16*3 hour sessions	Males and females the same age or a little older than participants. Most had further education or had undergone life skills training	Peer facilitators were selected whose attitudes were supportive of gender equity and non-judgemental regarding sexuality	3 weeks of training and 2 practice groups	Research staff made ad hoc visits to workshops that were in progress. Facilitators were observed and any issues related to the workshops were discussed with them, however there was no attempt to micro-manage the progress of intervention delivery	?	>1 incident of physical or sexual intimate partner violence (male)	OR 0.73 (0.50, 1.06)	0.099
												>1 incident of physical or sexual intimate partner violence (female)	OR 0.87 (0.64, 1.18)	0.36
												Rape or attempted rape (men)	OR 0.71 (0.47, 1.06)	0.094
**PHYSICAL DISORDERS**														
Al-Sheyab 2012	Peer facilitation only	Education	Peers came together in pairs and gave three 45-min lessons to Year 10 students on asthma self-management, using group discussions, videos, games, and problem-solving activities.	Groups	?	3 *45 min workshops	Year 11 students	?	Health workers delivered the content of the peer leader training programme.	?	?	Asthmatic quality of life	Mean difference 1.35 (1.04, 1.76)	0.02
Singhal 2010	Multi-component	Education, counselling	Student volunteers were trained to disseminate health messages through skits on nutrition-related topics such as the harmful effects of junk foods and healthy versus unhealthy lifestyles. Volunteers also gave recipe demonstrations and counselled junior students on how to select a healthy lunch.	Presentation, individual meetings	24	?	11th grade students	?	Weekly 1 hour training sessions	Supported by teachers and a nutritionist	?	BMI	Difference (-0.18, 0.34)	>0.05
**MENTAL DISORDERS**														
Balaji 2010	Multi-component	Education	Peer leaders were given a resource guide to help them to conduct group sessions and perform street plays to other youth in their communities in order to communicate information about intervention target issues. In each village, some youths were also trained to socially market condoms to other youth.	Groups, presentation	52	?	?	Selected by the research team	Trained by psychologists and social workers experienced in the field of adolescent health	Rural peer leaders were supported by a Community Advisory Board comprising of key people such as village council leaders. Urban peer leaders were supported by trained teachers and integrated into existing student forums	"Moderate" monetary and other incentives (certificates) provided	Probable depression (GHQ-12, rural)	OR 0.33 (0.23, 0.48)	<0.001
												Suicidal behaviour (rural)	OR 1.05 (0.28, 3.95)	0.94
												Probable depression (GHQ-12, urban)	OR 0.57 (0.41, 0.79)	0.001
												Suicidal behaviour (urban)	OR 0.38 (0.17, 0.84)	0.02
Church 2012	Peer facilitation only	Counselling	Peer facilitators provided Emotional Freedom Technique counselling group therapy	Groups	3	?	Students aged 24 or younger	?	Trained in EFT techniques	?	?	Depression (BDI)	Intervention mean 6.08 (SE 1.8); control mean 18.04 (SE 1.8)	0.001
Devries 2015	Multi-component	Education, counselling, activism	Students are selected to be members of the intervention-implementing committees in each school in order to contribute to decision-making and to be a role model for their peers. The intervention also involves students creating dramas and facilitating a ‘student court’ to handle school discipline issues.	Groups, presentation, policy engagement	76	?	Primary school students	Selected by peers or from existing student bodies	Members of the student court were trained by teacher ‘protagonists’ in positive discipline through role play and mock court sessions. Good school committee members were trained by Raising Voices staff and teachers using through manualised sessions	Supported by protagonist teachers and Raising Voices Staff	No financial incentive	Mental disorder symptoms (SDQ)	Difference 0.00 (-0.03, 0.03)	0.8907
Jewkes 2008	Peer facilitation only	Education	Peer-facilitated group education sessions involving roleplay and drama based on participants’ lived experiences. Groups are single sex for the first 13 sessions then there are three meetings where males and females come together, and a community meeting at the end of the program. Group sessions cover topics such as sex and love, conception and contraception, unwanted pregnancy and sexually transmitted diseases and HIV.	Groups	6–8 weeks	16*3 hour sessions	Males and females the same age or a little older than participants. Most had further education or had undergone life skills training	Peer facilitators were selected whose attitudes were supportive of gender equity and non-judgemental regarding sexuality	3 weeks of training and 2 practice groups	Research staff made ad hoc visits to workshops that were in progress. Facilitators were observed and any issues related to the workshops were discussed with them, however there was no attempt to micro-manage the progress of intervention delivery	?	Depression (CES-D, male)	OR 0.45 (0.16, 1.21)	0.11
												Depression (CES-D, female)	OR 1.32 (0.92, 1.89)	0.13
Sherman 2009	Peer facilitation only	Education, outreach	Peer education using a curriculum of group sessions to teach participants to think critically about and reduce their methamphetamine use and sexual risk behaviours. "Participants were taught communication skills that they practiced in role plays during the sessions and used to convey methamphetamine and risk reduction messages to specific social network members that were identified through a social network inventory administered at baseline."	Groups, individual meetings	4	?	Current drug users aged 18–25 years	?	Peer facilitators were trained by the researchers in an intensive week-long training. Index participants received 7 education sessions	?	Index participants were compensated 200 Baht ($5 USD) for each of the five study assessments and each of the seven intervention and control sessions, resulting in the opportunity to earn a total of $55 USD.	Depression (CES-D)	Control rate -0.092 (-0.018, -0.01); intervention rate -0.095 (-0.18- -0.01)	<0.05
Ssewamala 2010	Multi-component	Education	A mentorship component on life options and career planning, delivered by peer mentors	?	?	Monthly mentorship sessions. Total number of sessions is unclear	College-aged or college "bound" (for those in Senior Six vacation): ages 17–23.	?	?	?	?	Mental health functioning (Tennessee Self-Concept Scale)	β 3.48 (0.42, 6.55)	<0.05
												Depression (Children’s Depression Inventory)	β -0.34 (-0.61, -0.06)	0.02
Thurman 2016	Peer facilitation only	Education, counselling	Facilitators led manualised interpersonal psychotherapy group (IPTG) sessions to help adolescents learn how to resolve distress and to access emotional support from group members. Groups were divided by gender. Facilitators also led a curriculum-based group behavioural intervention addressing HIV risk factors and pathways, covering alcohol, substance abuse, crime and sexual violence, HIV/AIDS, healthy sexual relationships, transactional sex and condom use. The intervention aimed to encourage social learning through reflection. Groups were mixed gender to encourage dialogue and understanding from different gender perspectives	Groups	IPTG = 16/Vhutshilo = 13	IPTG = weekly 90 min sessions/Vhutshilo = weekly 60 min sessions	High school graduates aged 23–25 years old with relevant prior experience e.g. coaching youth sports teams and teaching Sunday school	Selected by the research team	10 day training by the research team	Social workers provided supervision for facilitators	Monthly stipend of USD 230	Depression (CES-DC)	β -0.53 (SE 1.05)	0.614
**SUBSTANCE USE**														
Ayaz 2015	Peer facilitation only	Education	Conducted group sessions for other school students using educational materials on smoking and its dangers. Sessions included discussion, question and answer, audiovisual devices (e.g. posters), and distribution of educational materials.	Groups	?	Unspecified number of 40 minute sessions	6th to 8th grade students aged 12–15 years	Co-selected by peers and teachers	Trained by researcher staff. Six training sessions in total lasting 40 min each. Peer educators completed pre and post tests to assess their proficiency	Sessions supervised by researchers	?	Smoking after peer education	χ2 3.056	0.08
Balaji 2010	Multi-component	Education	Peer leaders were given a resource guide to help them to conduct group sessions and perform street plays to other youth in their communities in order to communicate information about intervention target issues. In each village, some youths were also trained to socially market condoms to other youth.	Groups, presentation	52	?	?	Selected by the research team	Trained by psychologists and social workers experienced in the field of adolescent health	Rural peer leaders were supported by a Community Advisory Board comprising of key people such as village council leaders. Urban peer leaders were supported by trained teachers and integrated into existing student forums	"Moderate" monetary and other incentives (certificates) provided	Substance use (tobacco, cigarettes or alcohol) (rural)	OR 1.12 (0.8, 1.57)	0.52
												Substance use (tobacco, cigarettes or alcohol) (urban)	OR 0.63 (0.45, 0.89)	0.01
Chen 2014	Multi-component	Education, counselling, activism	Peer educators counselled their classmates to encourage them not to give or accept cigarettes during social activities, and to encourage smokers in their class to quit. Peer educators were also members of the school tobacco control group, which helped to develop and enforce school anti-smoking policies. They also organised educational group activities to share smoking prevention information with other students	Groups, policy engagement, individual meetings	52	?	Current students	Selected by peers	Trained on smoking prevention-related knowledge and communication skills	Teachers supported the organisation of group activities	?	Ever smoked (Linzhi)	OR 0.97 (0.71, 1.33)	>0.05
												Daily smoking (Linzhi)	OR 1.43 (0.82, 2.47)	>0.05
												Weekly smoking (Linzhi)	OR 1.63 (0.67, 3.95)	>0.05
												Current smoking (Linzhi)	OR 1.03 (0.69, 1.53)	>0.05
												Ever smoked (Guanghzou)	OR 0.87 (0.58, 1.32)	>0.05
												Daily smoking (Guanghzou)	OR 1.14 (0.40, 3.25)	>0.05
												Weekly smoking (Guanghzou)	OR 0.72 (0.06, 8.32)	>0.05
												Current smoking (Guanghzou)	OR 0.74 (0.31, 1.74)	>0.05
Harrell 2016	Multi-component	Education, outreach, counselling, activism	Trained peers led activities (films, street plays, games and role plays) and awareness rallies. Peer leaders were also involved in a group to enforce anti-tobacco policy by engaging tobacco vendors and promoted and monitored tobacco free zones.	Groups, presentation, policy engagement	104	At least six sessions (with films, street plays, games and role plays)	Community members aged 10–19 years	?	Trained by the project team at the beginning of each year	?	?	Current tobacco use	Control trajectory -0.10 (-0.24, -0.04); intervention trajectory -0.73 (-0.87, -0.59)	0.203
												Current smoking	Control trajectory -0.44 (-0.54, -0.34); intervention trajectory -0.65 (-0.77, -0.54)	0.328
												Current smokeless tobacco use	Control trajectory -0.76 (-0.91, -0.61); intervention trajectory -1.11 (-1.26, -0.96)	0.534
Jewkes 2008	Peer facilitation only	Education	Peer-facilitated group education sessions involving roleplay and drama based on participants’ lived experiences. Groups are single sex for the first 13 sessions then there are three meetings where males and females come together, and a community meeting at the end of the program. Group sessions cover topics such as sex and love, conception and contraception, unwanted pregnancy and sexually transmitted diseases and HIV.	Groups	6–8 weeks	16*3 hour sessions	Males and females the same age or a little older than participants. Most had further education or had undergone life skills training	Peer facilitators were selected whose attitudes were supportive of gender equity and non-judgemental regarding sexuality	3 weeks of training and 2 practice groups	Research staff made ad hoc visits to workshops that were in progress. Facilitators were observed and any issues related to the workshops were discussed with them, however there was no attempt to micro-manage the progress of intervention delivery	?	Problem drinking (AUDIT scale, male)	OR 0.68 (0.49, 0.94)	0.021
												Ever misused drugs (male)	OR 1.07 (0.65, 1.77)	0.78
												Problem drinking (AUDIT scale. female)	OR 0.94 (0.45, 1.95)	0.87
												Ever misused drugs (female)	OR 0.60 (0.29, 1.28)	0.19
Lotrean 2010	Peer facilitation only	Education	Peer leaders led classroom activity groups using material from an educational age appropriate video.	Groups	5	Weekly 45 min sessions	Students aged 13–14	?	1-hour information session before the start of the activities, providing information about the content and characteristics of the programme. Manuals summarising the content of the video and instructions for the activities were also given	Teachers helped to coordinate the sessions	?	Risk of non-smokers becoming regular smokers	OR 2.23 (1.20, 3.85)	<0.01
Perry 2009	Multi-component	Education, activism	Peer-led health activism outside of the classroom, including competitions between classrooms and schools.	Groups	104	Total of 14 peer-led classroom activities? More than 15 hours of activity overall	Students in the same classes as participants, aged 10–16 years	Election of students who were admired by their classmates	?	Manuals in local languages and continuous support of peer leaders by project staff	?	Chewing tobacco use, bidi smoking, cigarette smoking, any tobacco use	Control trajectory 0.94 (-0.10, 1.98); intervention trajectory -0.59 (-1.63, 0.45)	0.04
Sherman 2009	Peer facilitation only	Education, outreach	Peer education using a curriculum of group sessions to teach participants to think critically about and reduce their methamphetamine use and sexual risk behaviours. "Participants were taught communication skills that they practiced in role plays during the sessions and used to convey methamphetamine and risk reduction messages to specific social network members that were identified through a social network inventory administered at baseline."	Groups, individual meetings	4	?	Current drug users aged 18–25 years	?	Peer facilitators were trained by the researchers in an intensive week-long training. Index participants received 7 education sessions	?	Index participants were compensated 200 Baht ($5 USD) for each of the five study assessments and each of the seven intervention and control sessions, resulting in the opportunity to earn a total of $55 USD.	Methamphetamine use	OR 1.07 (0.79, 1.45)	>0.05
**EDUCATIONAL AND EMPLOYMENT MARGINALISATION**														
Carlson 2012	Peer facilitation only	Education	Facilitation of Young Citizen Program groups	Groups	28	Weekly 2–3 hour sessions	University and secondary school graduates, mostly aged 24 years or younger, with previous experience in youth-related HIV activities.	?	?	College-educated research staff supervised young adult peer facilitators	?	Academic self-efficacy	β 0.08 (-0.07, 0.22)	0.28
Ssewamala 2010	Multi-component	Education	A mentorship component on life options and career planning, delivered by peer mentors	?	?	Monthly mentorship sessions. Total number of sessions is unclear	College-aged or college "bound" (for those in Senior Six vacation): ages 17–23.	?	?	?	?	School attendance	F test 1.97	>0.05
												Planning to go on to secondary school	F test 8.11	≤0.01
												Planning to go to college or university	F test 1.36	>0.05
												Certainty to accomplish educational plans	F test 7.57	≤0.01

### Peer-facilitated strategies

Table 2 describes the characteristics of peer-facilitated intervention strategies, including the selection, training and supervision of peers. Interventions were diverse: peer facilitators conducted education, counselling, outreach and activism.

Nineteen of the 20 studies featured peer education activities. Peers ran group-based sessions for classmates and other students [32–36], facilitated groups in the community, [37–41] performed street plays or created dramas [37, 42–44], ran workshops with parents [45], and distributed educational materials [45, 46]. Nine of the 20 studies incorporated peer counselling strategies. These ranged from low intensity approaches where peers encouraged their classmates not to give or accept cigarettes [34], to higher intensity approaches where peers led manualised interpersonal psychotherapy groups [41]. Peer activism was used in five studies to develop and enforce anti-smoking/tobacco policies [34, 43], work with community leaders to provide opportunities for adolescents [45] and run a ‘student court’ to manage school discipline issues [42]. Peer outreach was used in four of the 19 studies. For example, in Thailand, peers used communication skills to convey risk reduction messages to drug users in their social networks [20]. As part of the CERCA (Community-Embedded Reproductive Health Care for Adolescents) intervention in Nicaragua, peers mentored adolescents to help them build decision-making competence related to sexual and reproductive health, and referred and accompanied them to health services when needed [45].

The duration of peer-facilitated components ranged from three weeks [47] to four years [39]. Training duration and intensity ranged from a one hour information session [35] to a four-week programme [46]. Peer facilitators were school students in nine of the 20 studies, and school graduates in six. Five studies did not provide information on the education level of facilitators.

Study quality was variable (Table 3): three studies were at low risk of bias across all seven domains [32, 40, 42]; 15 did not report methods used for allocation concealment; eight did not report methods for random sequence generation. One study was at high risk of bias because it had a small number of clusters and results were not adjusted for clustering or confounders [37]. In another, schools refused to participate after the baseline survey and it was not clear whether data were missing because of this or for other reasons [48]. Two studies encountered unexpectedly high rates of adolescent out-migration and were forced to change their study design substantially with implications for the statistical power of the study [39, 45]. In one study in Nicaragua, loss to follow up was 76%, with important differences between resurveyed adolescents and those lost to follow up [45].

**Table 3:** Risk of bias assessments of studies of peer-facilitated interventions for adolescent health.

First author and year of publication of main trial paper	Random sequence generation	Allocation concealment	Blinding of participants & personnel	Blinding of outcome assessment	Incomplete outcome data	Selective outcome reporting	Other bias
**Al-Sheyab 2012**	✓	✓	✓	✓	✓	✓	✓
**Ayaz 2015**	✓	?	✓	✓	?	?	✗
**Balaji 2010**	✓	?	✓	✓	✓	✓	✗
**Carlson 2012**	✓	?	✓	✓	✓	✓	✓
**Chen 2014**	?	?	✓	✓	✓	?	✗
**Church 2012**	?	?	✓	?	✗	?	?
**Cowan 2010**	?	?	✓	✓	✓	✓	✗
**Decat 2015**	?	?	✓	?	✗	✓	✗
**Devries 2015**	✓	✓	✓	✓	✓	✓	✓
**Harrell 2016**	✓	✓	✓	?	✓	✓	✓
**Jewkes 2008**	✓	✓	✓	✓	✓	✓	✓
**Lotrean 2010**	✓	?	✓	?	?	✗	✓
**Mmbaga 2017**	✓	✓	✓	?	✓	✓	✓
**Okonofua 2003**	✓	?	✓	?	✓	✓	✗
**Perry 2009**	?	?	✓	✓	✓	✓	✗
**Ross 2007**	?	?	✓	✓	✓	✓	✓
**Sherman 2009**	✓	?	✓	✓	✓	✗	✓
**Singhal 2010**	?	?	✓	?	?	✓	✗
**Ssewamala 2010**	?	?	✓	✓	✓	✓	✓
**Thurman 2016**	✓	?	✓	?	?	✗	✓

N.B. “✓ “represents a low risk of bias,”✗” high risk of bias and “?” unclear risk of bias.

### Study outcomes and intervention effects

We did not identify any studies focusing on infectious and vaccine preventable diseases, undernutrition or unintentional injuries. More studies measured outcomes related to sexual and reproductive health (nine studies), substance use (eight studies) and mental disorders (seven studies) than any other area of health need. Below, and in Table 2, we present intervention details and findings by area of health need.

#### HIV and AIDS

Four studies reported HIV/AIDS-related outcomes [20, 39, 40, 44]. All involved a community component and peer education. Two examined the effects of combining peer facilitation with programmes for parents, community stakeholders and health worker training [39, 44]. None of the four studies reported a positive effect of the interventions.

#### Sexual and reproductive health

Only one study [40] found an effect of peer-facilitated interventions on clinical sexual and reproductive health outcomes: Jewkes et al tested the effects of a structured curriculum of peer-facilitated group education on sex and love, contraception and sexually transmitted diseases among adolescent boys and girls in South Africa, and reported a reduction in Herpes Simplex Virus-2 infection (HSV-2) (RR 0.67 CI 0.47–0.97) [40]. However, other studies found negative results: one study from Tanzania reported an increased prevalence of gonorrhoea among young women (RR 1.93 CI 1.01–3.71) following school-based reproductive health education led by teachers followed by scripted dramas by peer educators [44]. Another study from Thailand used a curriculum of group education and role-play sessions to help young men and women reduce their use of metamphetamines and sexual risk-taking, and to communicate with others in their social networks about these risks [20]. The study found an increased incidence rate of gonorrhoea in the intervention group compared to the control group (4.69 per 100 person years vs. 0.43 per 100 person years, p<0.05).

Self-reported symptoms of sexually transmitted diseases (STDs) were reduced in two studies from India and Nigeria [37, 46]. In Balaji et al.’s Indian study, complaints of vaginal symptoms and penile discharge only decreased significantly in urban areas (OR: 0.49, 95% CI: 0.26–0.93 and OR: 0.36, 95% CI: 0.24–0.55, respectively), where peer facilitators were linked to schools [37]. Peers were also trained and supported within schools in the study by Okonofua et al, which reported a reduction in self-reported symptoms of STIs in Nigeria (OR 0.63 CI 0.43–0.91) [46]. A trial of peer-led after-school life skills training sessions reported an increase in condom use among boys (β 0.217 p = 0.004) in Tanzania. A South African trial of peer-led interpersonal psychotherapy groups to help adolescents learn how to resolve distress and access emotional support also led to girls reporting more condom use among their partners (β 0.21 p = 0.02 [36, 41]. Conversely, one trial of peer mentors helping adolescents build competence in making deliberate choices and referring them to health facilities reported reduced condom use (β -2.66 p = 0.039) [45].

#### Violence

Three studies reported violence-related outcomes, two of which found reductions in violence. Both of these successful interventions involved activities for teachers and adolescents and both used a combination of peer education, counseling and activism strategies. Devries et al evaluated the Good School Toolkit in Ugandan primary schools: students took part in intervention-implementing committees to reduce violence, create dramas and facilitate a student court to handle school discipline issues. They found reductions in past week and past term physical violence perpetrated by school staff, reported by students (past week: OR 0.39 CI 0.25–0.62; past term: OR 0.31 CI 0.18–0.53) [42]. They also reported a reduction in violence from peers, and a reduction in violence by school staff against adolescents who had functional difficulties and/or a disability [49]. Balaji et al.’s *Yuva Mitr* (Friend of Youth) intervention reduced perpetration of physical violence (rural areas OR 0.29 CI 0.15–0.57; urban areas OR 0.59 CI 0.40–0.87) and the experience of sexual violence (urban areas only: OR 0.19 CI 0.09–0.41) among adolescents in India [37]. Whilst the study by Devries et al. focused on reducing violence, *Yuva Mitr* sought to affect multiple areas of adolescent health through a multi-component intervention involving peer education, community activities, teacher training and dissemination of health materials.

#### Physical disorders

Only two studies reported outcomes relating to physical disorders. A school-based peer education intervention in Jordan improved quality of life among adolescents with asthma (mean difference 1.35 CI 1.04–1.76) [32]. An evaluation of a multicomponent school-based intervention to improve adolescent health and nutrition in India–judged to be at high risk of bias—measured no effect on BMI [50].

#### Mental disorders

Interventions for mental disorders were diverse and included peer outreach, counselling and education interventions that addressed determinants of mental health such as violence and substance use. Four of the seven studies with mental health outcomes reported improvements in depressive symptoms [19,36,46,50]. These four interventions were from diverse locations (Uganda, Philippines, India and Thailand) and involved a range of peer-facilitated strategies (education, outreach and counselling). Only one [47] of the four positive studies focused on an actual mental disorder, and reported a reduction in the severity of depression. Three of these four successful interventions were linked to schools or colleges [37, 47, 51].

#### Substance use

Four out of eight studies reporting substance use outcomes found positive effects. Interventions reduced alcohol drinking among young men (OR 0.68 CI 0.49–0.94) [40] and the risk of non-smokers becoming regular smokers (OR 2.23 CI 1.20–3.85) [35]. One study in urban schools in India tested Project MYTRI, a multi-component intervention with classroom curricula, a poster campaign and peer-led activism. The study found between-group differences in the rate of growth of cigarette smoking (p = 0.05), bidi smoking (p<0.01), and any tobacco use (p = 0.04) among students [48]. Among urban adolescents in India, Balaji et al reported a reduction in use of tobacco, cigarettes and alcohol (OR 0.63 CI 0.45–0.89) [37]. Three [35, 37, 48] of the four studies reporting positive effects were linked to schools, including two where school students acted as peer facilitators [35, 48].

#### Educational and employment marginalisation

Only two studies measured effects on educational and employment marginalisation [38, 51]. In Tanzania, the Young Citizens Programme aimed to develop adolescents’ individual and collective efficacy to raise awareness of HIV [38]. One outcome in this trial was academic self-efficacy (e.g. “I have learned how hard work helps me in math”), but there were no improvements in this outcome. The Suubi intervention in Uganda was aimed at AIDS-orphaned adolescents and involved a microfinance intervention, financial education and mentorship by older peers aged 17–23. Evaluation of the programme showed an increase in the number of adolescents saying they planned to go to secondary school and that they were more certain they could accomplish their education goals [51].

## Discussion

Our systematic review is the first to summarise results from trials of peer-facilitated interventions for all areas of adolescent health in LMICs: to our knowledge, the only other review of peer-facilitated interventions to assess effects for multiple health outcomes was conducted in 1999 and mainly included studies from high-income countries [28]. We found 20 trials focused on six of the nine areas identified by the Lancet Commission for Adolescent Health and Wellbeing: sexual and reproductive health, HIV/AIDS, physical disorders, mental health, violence, and substance use. There was some evidence that interventions improved mental health and reduced violence and substance use, but the diversity of components and outcomes prevented us from making definitive statements about effectiveness. We found no trials with positive effects on HIV-related outcomes, heterogenous results for physical disorders and sexual and reproductive health outcomes, and no trials on infectious and vaccine preventable diseases, undernutrition, or injuries.

Our review has three main limitations. The diversity of interventions and outcomes prevented us from meta-analysing the data within or across adolescent health areas. It also prevented us from understanding the extent to which facilitator characteristics, other intervention components and locations (e.g. school vs. non-school components) might explain heterogenous results within areas. To remedy this, future studies could provide more accurate descriptions of the content of interventions, and use comparable outcome measures within areas of adolescent health need. Further reviews could also focus on individual adolescent health areas and examine a broader range of study designs and methods.

A second limitation was our inability to assess publication bias. Although we contacted authors for clarifications, many articles screened lacked information about facilitator age, and we may not have identified all eligible studies [43, 45]. Risk of bias was variable across studies, with no specific pattern within and across areas.

Finally, several trials only included our outcomes of interest as secondary indicators. For example, some were powered to detect differences in sexual and reproductive health outcomes but also included outcomes related to violence and mental health [39]. Such trials may have been under-powered to detect significant differences between intervention and control arms for secondary indicators, and prone to false positive (Type I errors) due to multiple testing.

In line with previous systematic reviews, we found heterogeneous effects of peer-facilitated interventions on sexual and reproductive health, suggesting that peer facilitation alone is unlikely to be the solution to improving this area of health [15, 52, 53]. This is unsurprising given the breadth and strength of socio-political factors affecting sexuality and access to services for sexual and reproductive health.

We found more promising evidence for peer-facilitated interventions to improve adolescents’ mental health and reduce violence and substance use, but too much heterogeneity in interventions and outcomes to make definitive conclusions. Effects on mental health, violence and substance use have some plausibility: peer-facilitated interventions can strengthen peer networks, increase social support, change social norms and improve school environments [10, 54].

Fourteen out of 20 studies in our review examined interventions with a school- or college-based component, including three out of four studies with positive effects on depressive symptoms, and all positive studies on violence. There are many potential benefits to locating interventions in schools: there may be pre-existing support systems for peer facilitators, and facilitators have a ‘captive audience’ of participants in a classroom setting [55]. Potential disadvantages of working in schools include the potential for hierarchies between teachers, peer facilitators and participants to hinder communication, a lack of engagement with out-of-school adolescents, and the risk of entire schools dropping out of the intervention [28]. Previous studies have shown that using peer facilitators rather than teachers to deliver health education does not necessarily make an intervention more effective [28]. This may be because peer facilitation often involves implementing interventions developed by older adults. The benefits of such interventions could be lost if adolescents feel the intervention is no longer relevant or that they cannot relate to peer facilitators. Successful school-based interventions in this review were largely devised by research teams, though half consulted with young people during intervention design or implementation phases [37, 42, 46, 48]. More formalised involvement of adolescents in the development of peer-facilitated interventions is likely to be beneficial [24, 28, 52].

We identified two peer-facilitated interventions that engaged adolescents in peer leadership roles, and focused on capacity building rather than knowledge transfer [20, 38, 56]. These interventions had positive outcomes for mental health and self-efficacy (deliberative and communicative self-efficacy and emotional control). Interventions that engage a higher proportion of peer leaders may be more sustainable in populations with high rates of adolescent mobility, where retaining peer facilitators may be challenging. Interventions that engage peer facilitators in mobilising communities of young people have been successful in non-school settings [20, 38]. Reaching young people who are not in school is important to ensure equity. Offering them leadership opportunities through participatory interventions might help to achieve this.

Critically, twelve of the studies in this review involved interventions with additional, non-peer-facilitated components, with evidence of positive effects on mental disorders, violence and substance use. The enthusiasm for multi-component interventions—while challenging from the point of view of attribution—reflects the widespread acceptance that adolescent vulnerabilities are influenced by factors at multiple, interacting socio-ecological levels. Reviews of interventions for the prevention of violence have highlighted that interventions with multiple components that address these multiple layers are more likely to succeed than interventions that only address one [28]. These multi-component interventions require evaluations that theorise and assess the interaction between peer and non-peer-facilitated components, or the environment within which interventions are delivered as complex system [57, 58].

In conclusion, peer-facilitated community-based interventions show promise to improve mental health and reduce violence and substance use in LMICs, though further robust studies are needed to strengthen the evidence base. Future research should focus on theorising and assessing the contribution of peer-facilitated interventions and their interactions with non-peer-facilitated components in these areas of adolescent health.

## Supporting information

S1 Text Sample search strategy for Medline. (DOCX)

S2 Text Reasons for exclusion. (DOCX)

S1 Table Articles of potentially eligible registered studies or study protocols for which we did not find published results. (DOCX)

S2 Table Characteristics of studies of community-based peer-facilitated interventions for adolescent health. (DOCX)

S1 Checklist PRISMA 2009 Checklist. (DOC)

## Abbreviations

AIDS: autoimmune deficiency syndrome
BMI: body mass index
CERCA: Community-Embedded Reproductive Health Care for Adolescents
HIV: human immunodeficiency virus
HSV-2: herpes simplex virus-2 infection
LMICs: low- and middle-income countries
PRISMA: Preferred Reporting Items for Systematic Reviews and Meta-Analyses
RCT: randomised controlled trial
STD: sexually transmitted disease
STIs: sexually transmitted infection
