# Supplementary material for: Peer-facilitated community-based interventions for adolescent health in low- and middle-income countries: A systematic review
Source: PLoS One. 2019 Jan 23;14(1):e0210468. doi: 10.1371/journal.pone.0210468 (PMC6343892; doi:10.1371/journal.pone.0210468)
Supplement: S1 Text — (DOCX) [file pone.0210468.s001.docx]

**S1 text: Sample search strategy for Medline**

Search: Epub Ahead of Print, In-Process & Other Non-Indexed Citations, Ovid MEDLINE(R) Daily and Ovid MEDLINE(R) 1946 to Present

Executed: 03/06/2016 (updated 22/06/2018)

| **#** | **Searches** |
| --- | --- |
| 1 | Meta-Analysis as Topic/ |
| 2 | meta analy$.tw. |
| 3 | metaanaly$.tw. |
| 4 | Meta-Analysis/ |
| 5 | (systematic adj (review$1 or overview$1)).tw. |
| 6 | exp Review Literature as Topic/ |
| 7 | or/1-6 |
| 8 | cochrane.ab. |
| 9 | embase.ab. |
| 10 | (psychlit or psyclit).ab. |
| 11 | (psychinfo or psycinfo).ab. |
| 12 | (cinahl or cinhal).ab. |
| 13 | science citation index.ab. |
| 14 | bids.ab. |
| 15 | cancerlit.ab. |
| 16 | or/8-15 |
| 17 | reference list$.ab. |
| 18 | bibliograph$.ab. |
| 19 | hand-search$.ab. |
| 20 | relevant journals.ab. |
| 21 | manual search$.ab. |
| 22 | or/17-21 |
| 23 | selection criteria.ab. |
| 24 | data extraction.ab. |
| 25 | 23 or 24 |
| 26 | Review/ |
| 27 | 25 and 26 |
| 28 | Comment/ |
| 29 | Letter/ |
| 30 | Editorial/ |
| 31 | animal/ |
| 32 | human/ |
| 33 | 31 not (31 and 32) |
| 34 | or/28-30,33 |
| 35 | 7 or 16 or 22 or 27 |
| 36 | 35 not 34 |
| 37 | Randomized Controlled Trials as Topic/ |
| 38 | randomized controlled trial/ |
| 39 | Random Allocation/ |
| 40 | Double Blind Method/ |
| 41 | Single Blind Method/ |
| 42 | clinical trial/ |
| 43 | clinical trial, phase i.pt. |
| 44 | clinical trial, phase ii.pt. |
| 45 | clinical trial, phase iii.pt. |
| 46 | clinical trial, phase iv.pt. |
| 47 | controlled clinical trial.pt. |
| 48 | randomized controlled trial.pt. |
| 49 | multicenter study.pt. |
| 50 | clinical trial.pt. |
| 51 | exp Clinical Trials as topic/ |
| 52 | or/37-51 |
| 53 | (clinical adj trial$).tw. |
| 54 | ((singl$ or doubl$ or treb$ or tripl$) adj (blind$3 or mask$3)).tw. |
| 55 | PLACEBOS/ |
| 56 | placebo$.tw. |
| 57 | randomly allocated.tw. |
| 58 | (allocated adj2 random$).tw. |
| 59 | or/53-58 |
| 60 | 52 or 59 |
| 61 | case report.tw. |
| 62 | letter/ |
| 63 | historical article/ |
| 64 | or/61-63 |
| 65 | 60 not 64 |
| 66 | Developing Countries.sh,kf. |
| 67 | (Africa or Asia or Caribbean or West Indies or South America or Latin America or Central America).hw,kf,ti,ab,cp. |
| 68 | (Afghanistan or Albania or Algeria or Angola or Antigua or Barbuda or Armenia or Armenian or Aruba or Azerbaijan or Bahrain or Bangladesh or Barbados or Benin or Byelarus or Byelorussian or Belarus or Belorussian or Belorussia or Belize or Bhutan or Bolivia or Bosnia or Herzegovina or Hercegovina or Botswana or Brasil or Brazil or Bulgaria or Burkina Faso or Burkina Fasso or Upper Volta or Burundi or Urundi or Cambodia or Khmer Republic or Kampuchea or Cameroon or Cameroons or Cameron or Camerons or Cape Verde or Central African Republic or Chad or Chile or China or Colombia or Comoros or Comoro Islands or Comores or Mayotte or Congo or Zaire or Costa Rica or Cote d'Ivoire or Ivory Coast or Croatia or Cuba or Cyprus or Czechoslovakia or Czech Republic or Slovakia or Slovak Republic or Djibouti or French Somaliland or Dominica or Dominican Republic or East Timor or East Timur or Timor Leste or Ecuador or Egypt or United Arab Republic or El Salvador or Eritrea or Estonia or Ethiopia or Fiji or Gabon or Gabonese Republic or Gambia or Gaza or Georgia Republic or Georgian Republic or Ghana or Gold Coast or Greece or Grenada or Guatemala or Guinea or Guam or Guiana or Guyana or Haiti or Honduras or India or Maldives or Indonesia or Iran or Iraq or Isle of Man or Jamaica or Jordan or Kazakhstan or Kazakh or Kenya or Kiribati or Korea or Kosovo or Kyrgyzstan or Kirghizia or Kyrgyz Republic or Kirghiz or Kirgizstan or Lao PDR or Laos or Latvia or Lebanon or Lesotho or Basutoland or Liberia or Libya or Lithuania or Macedonia or Madagascar or Malagasy Republic or Malaysia or Malaya or Malay or Sabah or Sarawak or Malawi or Nyasaland or Mali or Malta or Marshall Islands or Mauritania or Mauritius or Agalega Islands or Mexico or Micronesia or Middle East or Moldova or Moldovia or Moldovian or Mongolia or Montenegro or Morocco or Ifni or Mozambique or Myanmar or Myanma or Burma or Namibia or Nepal or Netherlands Antilles or New Caledonia or Nicaragua or Niger or Nigeria or Northern Mariana Islands or Oman or Muscat or Pakistan or Palau or Palestine or Panama or Paraguay or Peru or Philippines or Philipines or Phillipines or Phillippines or Poland or Portugal or Puerto Rico or Romania or Rumania or Roumania or Russia or Russian or Rwanda or Ruanda or Saint Kitts or St Kitts or Nevis or Saint Lucia or St Lucia or Saint Vincent or St Vincent or Grenadines or Samoa or Samoan Islands or Navigator Island or Navigator Islands or Sao Tome or Saudi Arabia or Senegal or Serbia or Montenegro or Sierra Leone or Slovenia or Sri Lanka or Ceylon or Solomon Islands or Somalia or South Africa or Sudan or Suriname or Surinam or Swaziland or Syria or Tajikistan or Tadzhikistan or Tadjikistan or Tadzhik or Tanzania or Thailand or Togo or Togolese Republic or Tonga or Trinidad or Tobago or Tunisia or Turkey or Turkmenistan or Turkmen or Uganda or Ukraine or Uruguay or USSR or Soviet Union or Union of Soviet Socialist Republics or Uzbekistan or Uzbek or Vanuatu or New Hebrides or Vietnam or Viet Nam or West Bank or Yemen or Yugoslavia or Zambia or Zimbabwe or Rhodesia).hw,kf,ti,ab,cp. |
| 69 | ((developing or less* developed or under developed or underdeveloped or middle income or low* income or underserved or under served or deprived or poor*) adj (countr* or nation? or population? or world)).ti,ab. |
| 70 | ((developing or less* developed or under developed or underdeveloped or middle income or low* income) adj (economy or economies)).ti,ab. |
| 71 | (low* adj (gdp or gnp or gross domestic or gross national)).ti,ab. |
| 72 | (low adj3 middle adj3 countr*).ti,ab. |
| 73 | (lmic or lmics or third world or lami countr*).ti,ab. |
| 74 | transitional countr*.ti,ab. |
| 75 | or/66-74 |
| 76 | exp peer group/ or exp peer counselling/ or ((peer or teen* or adolesc* or youth or child* or (young adj person) or (young adj people) or (young adj adult) or (young adj wom*n) or (young adj female) or (young adj m*n) or (young adj male) or pupil* or student* or girl* or boy* or juvenile) adj3 (led or leader? or delivered or support* or approach or facilitat* or counsel* or educat* or promot* or train* or advisor? or tutor? or advocate? or teach* or instructor? or manage* or mentor* or assist* or directed or intervention* or group* or club*)).mp. |
| 77 | exp tuberculosis/ or tuberculosis.mp. or TB.mp. |
| 78 | exp malaria/ or malaria.mp. |
| 79 | exp meningitis/ or meningitis.mp. |
| 80 | exp Diarrhea/ or diarrh?ea.mp. |
| 81 | exp respiratory tract infections/ or lower respiratory tract infections.mp. or pneumonia.mp. |
| 82 | Intestin* infection.mp. |
| 83 | exp helminthiasis/ or exp intestinal diseases, parasitic/ |
| 84 | exp campylobacter infections/ or exp escherichia coli infections/ or exp paratyphoid fever/ or exp typhoid fever/ or exp vibrio infections/ or exp cholera/ or exp ascariasis/ or exp hookworm infections/ or exp ancylostomiasis/ or exp necatoriasis/ or exp anisakiasis/ or exp balantidiasis/ or exp blastocystis infections/ or exp cryptosporidiosis/ or exp dientamoebiasis/ or exp dysentery, amebic/ or exp giardiasis/ |
| 85 | exp Skin Diseases, Infectious/ or skin infection.mp. |
| 86 | exp cellulitis/ or cellulitis.mp. |
| 87 | exp Hepatitis, Viral, Human/ or viral hepatitis.mp. |
| 88 | measles.mp. or exp Measles/ |
| 89 | exp Rubella/ or rubella.mp. |
| 90 | influenza.mp. or exp Influenza, Human/ |
| 91 | exp diphtheria/ or diphtheria.mp. |
| 92 | exp Tetanus/ or tetanus.mp. |
| 93 | exp Vaccination/ or exp Immunization/ |
| 94 | (immunis* or immuniz* or vaccin*).mp. [mp=title, abstract, original title, name of substance word, subject heading word, keyword heading word, protocol supplementary concept word, rare disease supplementary concept word, unique identifier] |
| 95 | exp Anemia, Iron-Deficiency/ or iron deficiency an?emia.mp. |
| 96 | malnutrition.mp. or exp Malnutrition/ |
| 97 | underweight.mp. or exp Thinness/ |
| 98 | stunting.mp. or exp Growth Disorders/ |
| 99 | exp Wasting Syndrome/ or wasting.mp. |
| 100 | human immunodeficiency virus.mp. or exp HIV/ or HIV.mp. |
| 101 | exp acquired immunodeficiency syndrome/ or (acquired immunodeficiency syndrome or AIDS).mp. |
| 102 | sexually transmitted disease.mp. or exp Sexually Transmitted Diseases/ |
| 103 | exp Syphilis/ or syphillis.mp. |
| 104 | exp Herpes Simplex/ or herpes.mp. |
| 105 | exp Gonorrhea/ or gonorrhoea.mp. |
| 106 | exp Trichomonas Infections/ or trichomoniasis.mp. |
| 107 | chlamydia.mp. or exp Chlamydia Infections/ or exp Chlamydia/ |
| 108 | (human papilloma virus or hpv).mp. or exp Papillomavirus Infections/ |
| 109 | adolescent pregnancy.mp. or exp Pregnancy in Adolescence/ |
| 110 | (early or underage or "under age" or child or adolescent).mp. and (exp pregnancy/ or exp childbirth/ or pregnancy.mp. or childbirth.mp.) |
| 111 | (early or underage or "under age" or child or adolescent).mp. and (exp marriage/ or marriage.mp.) |
| 112 | exp contraception/ or contraception.mp. |
| 113 | exp contraceptive devices/ or contraceptive devices.mp. |
| 114 | exp contraceptive agents/ or contraceptive agents.mp. |
| 115 | maternal death.mp. or exp Maternal Mortality/ or exp Maternal Death/ |
| 116 | "Wounds and Injuries"/ or injur*.mp. |
| 117 | exp Drowning/ or drowning.mp. |
| 118 | burns/ or burn.mp. |
| 119 | exp Accidents, Traffic/ |
| 120 | violence.mp. or exp Violence/ |
| 121 | neglect.mp. |
| 122 | overweight.mp. or exp Overweight/ |
| 123 | exp Obesity/ or obesity.mp. or exp Obesity, Morbid/ |
| 124 | exp hemoglobinopathy/ or h*emoglobinopathy.mp. |
| 125 | exp Anemia, Hemolytic/ or h?emolytic an?emia.mp. |
| 126 | exp congenital abnormalities/ or congenital abnormalities.mp. |
| 127 | exp myocardial ischemia/ or isch?emic heart disease.mp. |
| 128 | exp low back pain/ or low back pain.mp. |
| 129 | exp neck pain/ or neck pain.mp. |
| 130 | exp asthma/ or asthma.mp. |
| 131 | exp migraine disorders/ or migraine.mp. |
| 132 | exp skin diseases/ or skin disease.mp. |
| 133 | exp sensation disorders/ or sensation disorders.mp. |
| 134 | exp eye diseases/ or refractive error*.mp. |
| 135 | exp anxiety/ or anxiety.mp. |
| 136 | exp autistic disorder/ or autism.mp. |
| 137 | exp conduct disorder/ or conduct disorder.mp. |
| 138 | exp depression/ or depression.mp. |
| 139 | exp Self-Injurious Behavior/ or exp suicide/ or self harm.mp. or suicide.mp. |
| 140 | exp Underage Drinking/ or exp Drinking/ or drinking.mp. |
| 141 | exp Alcohol-Related Disorders/ |
| 142 | alcohol drinking.mp. or exp Alcohol Drinking/ |
| 143 | "tobacco use".mp. or exp "Tobacco Use"/ |
| 144 | exp Substance-Related Disorders/ or drug misuse.mp. |
| 145 | exp education/ or education.mp. |
| 146 | employment.mp. or exp Employment/ |
| 147 | exp vocational education/ or vocational training.mp. |
| 148 | unemployment.mp. or exp Unemployment/ |
| 149 | 36 or 65 |
| 150 | or/77-148 |
| 151 | 75 and 76 and 149 and 150 |
| 152 | limit 151 to (humans and ("child (6 to 12 years)" or "adolescent (13 to 18 years)" or "young adult (19 to 24 years)")) |
