# Supplementary material for: Peer-facilitated community-based interventions for adolescent health in low- and middle-income countries: A systematic review
Source: PLoS One. 2019 Jan 23;14(1):e0210468. doi: 10.1371/journal.pone.0210468 (PMC6343892; doi:10.1371/journal.pone.0210468)
Supplement: S2 Text — (DOCX) [file pone.0210468.s002.docx]

**S2 text: Reasons for exclusion**

Excluded studies^[[1]](#footnote-1)^ are listed under each reason using a reference number from the reference list below:

**Cannot locate full text (n=11)**

Studies: [1-11]

**Conference proceedings (n=13)**

Studies: [12-24]

**Did not measure a relevant outcome (n=29)**

Studies: [25-53]

**High-income country setting (n=33)**

Studies: [54-86]

**No peer-facilitated intervention component (n=124)**

Studies: [87-210]

**Not a community-based intervention (n=3)**

Studies: [211-213]

**Not an RCT (n=111)**

Studies: [214-324]

**Participants <10 years old (n=26)**

Studies: [325-350]

**Participants >19 years old (n=96)**

Studies: [351-446]

**Protocol for a study not meeting the inclusion criteria (n=313)**

Studies: [447-759]

**Review (n=279)**

Studies: [760-1038]

**Unclear age of facilitators (n=7)**

Studies: [1039-1045]

**Wrong comparator (n=7)**

Studies: [1046-1052]

**References**

1. Daboer JC, Ogbonna C, Jamda MA Impact of Health Education on Sexual Risk Behaviour of Secondary School Students in Jos, Nigeria. Nigerian Journal of Medicine 17: 324-329.

2. Green SDR Caring for children with HIV/AIDS in rural Zaire. NU Nytt Om U-Landshälsovard 6: 10-12.

3. Kaddu JB, Gitta P, Mawejje D, et al. Children as Partners in the Struggle Against AIDS and Care for PWAS. The Uganda Health Information Digest 1: 29-29.

4. Ntege BK, Musasizi M, Mutumba R An Outreach Youth Specific AIDS Education Programme Targeting Young People in Uganda: the Young People&#039;s Initiative. The Uganda Health Information Digest 37-38.

5. Wani G Child-to-Child Programme in Uganda. The Uganda Health Information Bulletin 1: 17-17.

6. Ogutu RO, Oloo AJ, Ekissa WS, Genga IO, Mulaya N, et al. (1992) The effect of participatory school health programme on the control of malaria. pp. 298-302.

7. Wang X, Zhu Y (2009) [Peer education's effects on preventing accidental injuries in middle school students]. [Chinese]. Wei sheng yan jiu = Journal of hygiene research 38: 449-451.

8. Nayak BS, Bhat HV (2010) A study to evaluate the effectiveness of multicomponent intervention on lifestyle practices, body fat and self esteem of obese/overweight school children in selected English medium schools of Udupi district, Karnataka. International Journal of Nursing Education 2: 9-12 14p.

9. Wang L, Sun MX, Wang MF, Yan Y, Li BW, et al. (2011) Effects of different interventions on body mass index and body fat content in overweight and obese adolescents. [Chinese]. pp. 16-18.

10. Wang Y, Xu XY, Cao HM, Zhang XR, Wang JL, et al. (2014) Evaluation of intervention effect of peer-led education on sexual health of rural left-behind middle school students in Sichuan. [Chinese]. pp. 719-724+730.

11. Cueto C, Jacobson J, Smith J, Mumbengegwi D, Sturrock H, et al. (2017) Active surveillance through peer referral to identify populations at higher risk for malaria in Zambezi region, Namibia. American journal of tropical medicine and hygiene Conference: 64th annual meeting of the american society of tropical medicine and hygiene, ASTMH 2015 United states Conference start: 20151025 Conference end: 20151029. pp. 472-473.

12. Abrams AL, Siegfried NL, Ghersi D (2010) Filling the Gaps: The World Health Organization and the Pan African Clinical Trials Registry efforts to combat an unclear child-focused clincal trial landscape. Poster presentation at the Joint Cochrane and Campbell Colloquium; 2010 Oct 18-22; Keystone, Colorado, USA [abstract]. pp. 74-75.

13. Al-sheyab NA, Shah S, Gallagher R, Crisp J (2010) Effectiveness of a peer-led education program for adolescents with asthma in Jordanian schools.

14. Zhang L-s, Yang J, Zhu Y, Huang L, Zhou QY (2011) An Comprehensive Intervention Experiment on Psychological Health Development of Rural Left-behind Children. 85-90 p.

15. Manani R, Bahrami F, Abedi A (2012) Effectiveness of child centered attachment-based therapy on reducing childhood depression symptoms in early adolescent students in Isfahan, Iran. pp. 99-100.

16. Wirtz A, Pretorius C, Sherman S, Baral S, Decker M, et al. (2012) Modeling the impacts of a comprehensive community empowerment-based, HIV prevention intervention for female sex workers in generalized and concentrated epidemics: Infections averted among sex workers and adults. Journal of the International AIDS Society 15: 107.

17. Kaufman ZA, Kaufman EB, Dringus S, Weiss HA, Delany-Moretlwe S, et al. (2013) Baseline results: Of a cluster-randomised trial assessing the effectiveness of sport-based HIV Prevention in South African Schools.

18. Verstraeten R, Maes L, Kolsteren P (2013) Design and process evaluation of a paired-matched randomized controlled health promotion trial in ecuador. Annals of Nutrition and Metabolism 63: 471.

19. (2013) 2013 SYR Accepted Poster Abstracts. International journal of yoga therapy 23 Suppl: 32-53.

20. Anonymous (2013) 1st Annual Scientific Meeting of the European-Association-of-Psychosomatic-Medicine (EAPM), Cambridge, UK, July 04 -06, 2013. Journal of psychosomatic research 74: 539-562.

21. Decat P, Meyer S, Jaruseviciene L (2014) Community-based interventions promoting adolescents' sexual health in three Latin-American cities: Impact and impact modifying factors. pp. S76.

22. Macias M, Avila-Huerta C, Roca-Chiapas J, Garay-Sevilla M (2014) Effectiveness of program "5 steps for health" in scholar children in Mexico". pp. A236.

23. Xu F, Leslie E, Ware R, Tse L, Wang Z, et al. (2014) The effectiveness of a cluster-randomized controlled childhood obesity prevention study among Chinese primary school students: CLICK-obesity. pp. 236.

24. (2015) Abstracts of 21st FIGO World Congress of Gynecology and Obstetrics.

25. Andersen FL, Tolley HD, Schantz PM, Chi P, Liu F, et al. (1991) Cystic echinococcosis in the Xinjiang/Uygur Autonomous Region, People's Republic of China. II. Comparison of three levels of a local preventive and control program. pp. 1-10.

26. Rusakaniko S, Mbizvo MT, Kasule J, Gupta V, Kinoti SN, et al. (1997) Trends in reproductive health knowledge following a health education intervention among adolescents in Zimbabwe. pp. 1-6.

27. Agha S (2002) An evaluation of the effectiveness of a peer sexual health intervention among secondary-school students in Zambia. pp. 269-281.

28. Ilika AL, Obionu CO (2002) Personal hygiene practice and school-based health education of children in Anambra State, Nigeria. pp. 79-82.

29. Sánchez C S, Atencio L G, Duy N, Grande B M, Flores O M, et al. (2003) Comparación de la educación por pares y por profesionales de la salud para mejorar el conocimiento, percepción y la conducta sexual de riesgo en adolescentes. Revista Peruana de Medicina Experimental y Salud Publica 20: 206-210.

30. Agha S, Rossem R (2004) Impact of a school-based peer sexual health intervention on normative beliefs, risk perceptions, and sexual behavior of Zambian adolescents. pp. 441-452.

31. Parwej S, Kumar R, Walia I, Aggarwal AK (2005) Reproductive health education intervention trial. Indian Journal of Pediatrics 72: 287-291 285p.

32. (2008) Children with diabetes at school. West African Journal of Nursing 19: 79-79 71p.

33. Peña R, Quintanilla M, Navarro K, Martinez J, Castillo V, et al. (2008) Evaluating a peer intervention strategy for the promotion of sexual health-related knowledge and skills in 10- to 14-year old girls: findings from the 'Entre Amigas' project in Nicaragua. American Journal of Health Promotion 22: 275-281 277p.

34. Cai Y, Hong H, Shi R, Ye X, Xu G, et al. (2008) Long-term follow-up study on peer-led school-based HIV/AIDS prevention among youths in Shanghai. pp. 848-850.

35. Huang H, Ye X, Cai Y, Shen L, Xu G, et al. (2008) Study on peer-led school-based HIV/AIDS prevention among youths in a medium-sized city in China. pp. 342-346.

36. Gallegos EC, Villarruel AM, Loveland-Cherry C, Ronis DL, Zhou Y (2008) Intervention to reduce adolescents' sexual risk behaviors: A randomized controlled trial. [Spanish] Intervencion para reducir riesgo en conductas sexuales de adolescentes: Un ensayo aleatorizado y controlado. Salud Publica de Mexico 50: 59-66.

37. Taymoori P, Niknami S, Berry T, Lubans D, Ghofranipour F, et al. (2008) A school-based randomized controlled trial to improve physical activity among Iranian high school girls. International Journal of Behavioral Nutrition and Physical Activity 5 (no pagination).

38. Bate SL, Stigler MH, Thompson MS, Arora M, Perry CL, et al. (2009) Psychosocial mediators of a school-based tobacco prevention program in India: results from the first year of project MYTRI. pp. 116-128.

39. Cai Y, Shi R (2009) Follow-up study of peer education program on HIV/AIDS among senior high school students in Shanghai. [Chinese]. pp. 288-290.

40. Li S, Huang H, Cai Y, Ye X, Shen X, et al. (2010) Evaluation of a school-based HIV/AIDS peer-led prevention programme: The first intervention trial for children of migrant workers in China. pp. 82-86.

41. Liu XX, Wang H, Huang RR, Liu Q, Wang Y (2010) Instant and long-term effect comparison between peer education and traditional health education in tuberculosis prevention at middle schools of Chongqing three gorge areas. [Chinese]. pp. 449-453.

42. Kumar De A, Guha P, Chatterjee S, Singh YT, Singh LR, et al. (2010) Reproductive and sexual health issues of adolescent school children and impact of sensitization. JMS - Journal of Medical Society 24: 66-70.

43. Singhal N, Misra A, Shah P, Gulati S, Bhatt S, et al. (2011) Impact of intensive school-based nutrition education and lifestyle interventions on insulin resistance, beta-cell function, disposition index, and subclinical inflammation among asian indian adolescents: A controlled intervention study. pp. 143-150.

44. Cui Z, Shah S, Yan L, Pan Y, Gao A, et al. (2012) Effect of a school-based peer education intervention on physical activity and sedentary behaviour in Chinese adolescents: A pilot study.

45. Durán CE, Bahena EN, Rodríguez Mde L, Baca GJ, Uresti AS, et al. (2012) Near-peer teaching in an anatomy course with a low faculty-to-student ratio. pp. 171-176.

46. Sanaeinasab H, Saffari M, Pakpour AH, Nazeri M, Piper CN (2012) A model-based educational intervention to increase physical activity among Iranian adolescents. pp. 430-438.

47. Djalalinia S, Tehrani FR, Afzali HM, Hejazi F, Peykari N (2012) Parents or school health trainers, which of them is appropriate for menstrual health education? International Journal of Preventive Medicine 3: 622-627.

48. Lotrean LM, Loghin CR, Popa M, Vries HD (2013) Smoking prevention for adolescents in Romanian schools. pp. 7017-7021.

49. de Moura IH, Nadabe Silva A, Silvanéia dos Anjos J, Uchôa de Castro TH, de Almeida PC, et al. (2015) Educational strategies with adolescents at risk from diabetes mellitus: a comparative study. Online Brazilian Journal of Nursing 14: 25-31 27p.

50. Aninanya GA, Debpuur CY, Awine T, Williams JE, Hodgson A, et al. (2015) Effects of an adolescent sexual and reproductive health intervention on health service usage by young people in northern Ghana: a community-randomised trial. PLoS ONE [Electronic Resource] 10: e0125267.

51. Sushanth VH, Krishna M, Suresh Babu AM, Prashant GM, Chandu GN (2011) A peer group approach model of oral health promotion among orphans at Puduchery, South India. Journal of International Society of Preventive & Community Dentistry 1: 71-75.

52. Moshki M, Zamani-Alavijeh F, Mojadam M (2017) Efficacy of peer education for adopting preventive behaviors against head lice infestation in female elementary school students: A randomised controlled trial. PLoS ONE 12 (1) (no pagination).

53. Sukys S, Majauskiene D, Dumciene A (2017) The effects of a three-year integrated Olympic education programme on adolescents' prosocial behaviours. European journal of sport science 17: 335-342.

54. Wodarski JS (1987) Teaching adolescents about alcohol and driving: a two year follow-up. pp. 327-344.

55. Rickert VI, Gottlieb A, Jay MS (1990) A COMPARISON OF 3 CLINIC-BASED AIDS EDUCATION-PROGRAMS ON FEMALE ADOLESCENTS KNOWLEDGE, ATTITUDES, AND BEHAVIOR. Journal of Adolescent Health 11: 298-303.

56. Resnicow K, Davis M, Smith M, Baranowski T, Lin LS, et al. (1998) Results of the TeachWell worksite wellness program. pp. 250-257.

57. Stone EJ, Norman JE, Davis SM, Stewart D, Clay TE, et al. (2003) Design, implementation, and quality control in the Pathways American-Indian multicenter trial. Preventive Medicine 37: S13-23 11p.

58. Lau PWC, Yu CW, Lee A, Sung RYT (2004) The physiological and psychological effects of resistance training on Chinese obese adolescents. Journal of Exercise Science & Fitness 2: 115-120 116p.

59. Cabezón C, Vigil P, Rojas I, Leiva ME, Riquelme R, et al. (2005) Adolescent pregnancy prevention: An abstinence-centered randomized controlled intervention in a Chilean public high school. pp. 64-69.

60. Reynolds KD, Buller DB, Yaroch AL, Maloy JA, Cutter GR (2006) Mediation of a middle school skin cancer prevention program. Health Psychology 25: 616-625 610p.

61. Merakou K, Kourea KJ (2006) Peer education in HIV prevention: An evaluation in schools. pp. 128-132.

62. Huang S-H, Weng K-P, Hsieh K-S, Ou S-F, Lin C-C, et al. (2007) Effects of a classroom-based weight-control intervention on cardiovascular disease in elementary-school obese children. Acta Paediatrica Taiwanica 48: 201-206.

63. Malow RM, Stein JA, McMahon RC, Dévieux JG, Rosenberg R, et al. (2009) Effects of a culturally adapted HIV prevention intervention in Haitian youth. pp. 110-121.

64. Shechtman Z, Mor M (2010) Groups for children and adolescents with trauma-related symptoms: outcomes and processes. pp. 221-244.

65. Wyman PA, Brown CH, LoMurray M, Schmeelk-Cone K, Petrova M, et al. (2010) An outcome evaluation of the Sources of Strength suicide prevention program delivered by adolescent peer leaders in high schools. pp. 1653-1661.

66. Berry D, Colindres M, Sanchez-Lugo L, Sanchez M, Neal M, et al. (2011) Adapting, Feasibility Testing, and Pilot Testing a Weight Management Intervention for Recently Immigrated Spanish-Speaking Women and Their 2- to 4-Year-Old Children. Hispanic Health Care International 9: 186-193 188p.

67. Dkang KD, Choi JW, Kang SG, Han DH (2011) Sports Therapy for Attention, Cognitions and Sociality. International Journal of Sports Medicine 32: 953-959 957p.

68. Wyatt TJ, Oswalt SB (2011) Letting Students Be Innovative! Using Mini-Grants to Fund Student-Designed HIV/AIDS Education. Health promotion practice 12: 414-424 411p.

69. Gargano LM, Pazol K, Sales JM, Painter JE, Morfaw C, et al. (2011) Multicomponent interventions to enhance influenza vaccine delivery to adolescents. pp. e1092-1099.

70. Ghorob A, Vivas MM, Vore D, Ngo V, Bodenheimer T, et al. (2011) The effectiveness of peer health coaching in improving glycemic control among low-income patients with diabetes: protocol for a randomized controlled trial. pp. 208.

71. Bihan H, Méjean C, Castetbon K, Faure H, Ducros V, et al. (2012) Impact of fruit and vegetable vouchers and dietary advice on fruit and vegetable intake in a low-income population. pp. 369-375.

72. Ruger JP, Prah Ruger J, Abdallah AB, Luekens C, Cottler L (2012) Cost-effectiveness of peer-delivered interventions for cocaine and alcohol abuse among women: a randomized controlled trial. pp. e33594.

73. Robinson J, Yuen HP, Gook S, Hughes A, Cosgrave E, et al. (2012) Can receipt of a regular postcard reduce suicide-related behaviour in young help seekers? A randomized controlled trial. Early Intervention in Psychiatry 6: 145-152.

74. Silva B, Ferrada C, Santolaya ME (2012) Impact of an educational intervention on knowledge about appropriate use of antimicrobials in respiratory tract infections in adolescents. Revista Chilena de Infectologia 29: 499-502.

75. Leung KC, Marsh HW, Craven RG, Yeung AS, Abduljabbar AS (2013) Domain Specificity Between Peer Support and Self-Concept. Journal of Early Adolescence 33: 227-244 218p.

76. Ozer EJ, Douglas L (2013) The impact of participatory research on urban teens: an experimental evaluation. pp. 66-75.

77. Wang B, Stanton B, Knowles V, Russell-Rolle G, Deveaux L, et al. (2014) Sustained institutional effects of an evidence-based HIV prevention intervention. pp. 340-349.

78. Zhai F, Brooks-Gunn J, Waldfogel J (2014) Head Start's impact is contingent on alternative type of care in comparison group. pp. 2572-2586.

79. Dinaj-Koci V, Lunn S, Deveaux L, Wang B, Chen X, et al. (2014) Adolescent age at time of receipt of one or more sexual risk reduction interventions. Journal of Adolescent Health 55: 228-234.

80. Sibley MH, Altszuler AR, Ross JM, Sanchez F, Pelham WE, et al. (2014) A Parent-Teen Collaborative Treatment Model for Academically Impaired High School Students With ADHD. Cognitive and Behavioral Practice 21: 32-42.

81. Archibald CM, Newman D (2015) Pilot Testing HIV Prevention in an Afro Caribbean Faith-Based Community. pp. 43-49.

82. Dinaj-Koci V, Deveaux L, Wang B, Lunn S, Marshall S, et al. (2015) Adolescent Sexual Health Education: Parents Benefit Too! Health education & behavior 42: 648-653.

83. Dinaj-Koci V, Chen X, Deveaux L, Lunn S, Li X, et al. (2015) Developmental implications of HIV prevention during adolescence: Examination of the long-term impact of HIV prevention interventions delivered in randomized controlled trials in grade six and in grade 10. Youth & Society 47: 151-172.

84. Zarrouk L, Jellali IA, Hajji K, Youness S, Marrag I, et al. (2015) Impact of a psycho-educational intervention on quality-of-life among patients with type I bipolar disorder. Evolution Psychiatrique 80: 793-808.

85. Higley E, Walker SC, Bishop AS, Fritz C (2016) Achieving high quality and long-lasting matches in youth mentoring programmes: a case study of 4 Results mentoring. Child & Family Social Work 21: 240-248 249p.

86. Khanal S, Welsby D, Lloyd B, Innes-Hughes C, Lukeis S, et al. (2016) Effectiveness of a once per week delivery of a family-based childhood obesity intervention: a cluster randomised controlled trial. Pediatric Obesity 11: 475-483.

87. Richter-Strydom LM, Griesel RD, Glatthaar I (1985) Effects of a nutrition education programme on the psychological performance of malnourished children. A 3-year follow-up study. South African Medical Journal Suid-Afrikaanse Tydskrif Vir Geneeskunde 68: 659-662.

88. Aplasca MRA, Siegel D, Mandel JS, Santana-Arciaga RT, Paul J, et al. (1995) Results of a model AIDS prevention program for high school students in the Philippines. Aids 9: S7-S13.

89. Klepp KI, Ndeki SS, Leshabari MT, Hannan PJ, Lyimo BA (1997) AIDS education in Tanzania: promoting risk reduction among primary school children. pp. 1931-1936.

90. Mayaud P, Mosha F, Todd J, Balira R, Mgara J, et al. (1997) Improved treatment services significantly reduce the prevalence of sexually transmitted diseases in rural Tanzania: results of a randomized controlled trial. pp. 1873-1880.

91. Mbizvo MT, Kasule J, Gupta V, Rusakaniko S, Kinoti SN, et al. (1997) Effects of a randomized health education intervention on aspects of reproductive health knowledge and reported behaviour among adolescents in Zimbabwe. pp. 573-577.

92. Antunes MC, Stall RD, Paiva V, Peres CA, Paul J, et al. (1997) Evaluating an AIDS sexual risk reduction program for young adults in public night schools in Sao Paulo, Brazil. Aids 11 Suppl 1: S121-127.

93. Pokharel GP, Pant CR, Tilden RL, Pokhrel RP, Atmarita n, et al. (1998) Nutrition education and mega-dose vitamin A supplementation in Nepal. pp. 547-555.

94. Zivkovic M, Bjegovic V, Vukovic D, Marinkovic J (1998) [Evaluation of the effect of the health education intervention project 'Healthy School']. pp. 164-170.

95. Harvey B, Stuart J, Swan T (2000) Evaluation of a drama-in-education programme to increase AIDS awareness in South African high schools: a randomized community intervention trial. pp. 105-111.

96. Lou CH, Wang B, Shen Y, Gao ES (2004) Effects of a community-based sex education and reproductive health service program on contraceptive use of unmarried youths in Shanghai. pp. 433-440.

97. Micheli D, Fisberg M, Formigoni ML (2004) [Study on the effectiveness of brief intervention for alcohol and other drug use directed to adolescents in a primary health care unit]. pp. 305-313.

98. De Mello ED, Luft VC, Meyer F (2004) Individual outpatient care versus group education programs. Which leads to greater change in dietary and physical activity habits for obese children? Jornal de Pediatria 80: 468-474.

99. Zhang QH, Yue YL, Liu SQ, Li YQ, Lu AH (2004) Comprehensive intervention in children with simple obesity: Two-year effect observation. [Chinese]. Chinese Journal of Clinical Rehabilitation 8: 5084-5086.

100. (2005) [Nursing initiative for education for girls in Africa]. Pflege Aktuell 59: 696-697 692p.

101. Kaljee LM, Genberg B, Riel R, Cole M, Thoa LH, et al. (2005) Effectiveness of a theory-based risk reduction HIV prevention program for rural Vietnamese adolescents. AIDS Education & Prevention 17: 185-199 115p.

102. Nithikathkul C, Akarachantachote N, Wannapinyosheep S, Pumdonming W, Brodsky M, et al. (2005) Impact of health educational programmes on the prevalence of enterobiasis in schoolchildren in Thailand. Journal of Helminthology 79: 61-65.

103. Walker D, Gutierrez JP, Torres P, Bertozzi SM (2006) HIV prevention in Mexican schools: prospective randomised evaluation of intervention. BMJ: British Medical Journal (International Edition) 332: 1189-1192 1184p.

104. James S, Reddy P, Ruiter RAC, McCauley A, Borne B (2006) The impact of an HIV and AIDS life skills program on secondary school students in Kwazulu-Natal, South Africa. pp. 281-294.

105. Lou Ch, Zhao Q, Gao ES, Shah IH (2006) Can the internet be used effectively to provide sex education to young people in China? pp. 720-728.

106. Seal N (2006) Preventing tobacco and drug use among Thai high school students through life skills training. pp. 164-168.

107. Wang B, Li Y (2006) Cognition-behavior therapy for cultivating positive emotions in senior high school students. [Chinese]. Chinese Journal of Clinical Rehabilitation 10: 79-81.

108. Torres P, Walker DM, Gutierez JP, Bertozzi SM (2006) [A novel school-based strategy for the prevention of HIV/AIDS, sexually transmitted disease (STDs), and teen pregnancies]. Salud Publica de Mexico 48: 308-316.

109. Bolton P, Bass J, Betancourt T, Speelman L, Onyango G, et al. (2007) Interventions for depression symptoms among adolescent survivors of war and displacement in northern Uganda: a randomized controlled trial. pp. 519-527.

110. Lightfoot MA, Kasirye R, Comulada WS, Rotheram-Borus MJ (2007) Efficacy of a culturally adapted intervention for youth living with HIV in Uganda. pp. 271-273.

111. Bell CC, Bhana A, Petersen I, McKay MM, Gibbons R, et al. (2008) Building protective factors to offset sexually risky behaviors among black youths: a randomized control trial. Journal of the National Medical Association 100: 936-944 939p.

112. Banerjee AV, Banerji R, Duflo E, Glennerster R, Khemani S (2008) Pitfalls of Participatory Programs: Evidence from a Randomized Evaluation in Education in India.

113. Kain J, Uauy R, Leyton B, Cerda R, Olivares S, et al. (2008) [Effectiveness of a dietary and physical activity intervention to prevent obesity in school age children]. pp. 22-30.

114. Layne CM, Saltzman WR, Poppleton L, Burlingame GM, Pasalić A, et al. (2008) Effectiveness of a school-based group psychotherapy program for war-exposed adolescents: a randomized controlled trial. pp. 1048-1062.

115. Resnicow K, Reddy SP, James S, Gabebodeen Omardien R, Kambaran NS, et al. (2008) Comparison of two school-based smoking prevention programs among South African high school students: results of a randomized trial. pp. 231-243.

116. Wang JS (2008) The effects of swimming intervention for children with asthma [Abstract]. pp. A145 [p141-116].

117. Simao MO, Kerr-Correa F, Smaira SI, Trinca LA, Floripes TM, et al. (2008) Prevention of "risky" drinking among students at a Brazilian university.[Erratum appears in Alcohol Alcohol. 2008 Sep-Oct;43(5):608]. Alcohol & Alcoholism 43: 470-476.

118. Baptiste DR, Kapungu C, Miller S, Crown L, Henry D, et al. (2009) Increasing parent involvement in youth HIV prevention: a randomized Caribbean study. AIDS Education & Prevention 21: 495-511 417p.

119. Kumakech E, Cantor-Graae E, Maling S, Bajunirwe F (2009) Peer-group support intervention improves the psychosocial well-being of AIDS orphans: Cluster randomized trial. Social Science & Medicine 68: 1038-1043 1036p.

120. Barros MV, Nahas MV, Hallal PC, Farias Júnior JC, Florindo AA, et al. (2009) Effectiveness of a school-based intervention on physical activity for high school students in Brazil: the Saude na Boa project. pp. 163-169.

121. Berger R, Gelkopf M (2009) School-based intervention for the treatment of tsunami-related distress in children: a quasi-randomized controlled trial. pp. 364-371.

122. Jahanfar S, Lye MS, Rampal L (2009) A randomised controlled trial of peer-adult-led intervention on improvement of knowledge, attitudes and behaviour of university students regarding HIV/AIDS in Malaysia. pp. 173-180.

123. Nahas MV, Barros MV, Assis MA, Hallal PC, Florindo AA, et al. (2009) Methods and participant characteristics of a randomized intervention to promote physical activity and healthy eating among brazilian high school students: the Saude na Boa project. pp. 153-162.

124. Guo R, He Q, Shi J, Gong J, Wang H, et al. (2010) Short-term impact of cognition-motivation-emotional intelligence-resistance skills program on drug use prevention for school students in Wuhan, China. pp. 720-725.

125. Jordans MJ, Komproe IH, Tol WA, Kohrt BA, Luitel NP, et al. (2010) Evaluation of a classroom-based psychosocial intervention in conflict-affected Nepal: a cluster randomized controlled trial. pp. 818-826.

126. Li YP, Hu XQ, Schouten EG, Liu AL, Du SM, et al. (2010) Report on childhood obesity in China (8): effects and sustainability of physical activity intervention on body composition of Chinese youth. pp. 180-187.

127. Sakuma KL, Sun P, Unger JB, Johnson CA (2010) Evaluating depressive symptom interactions on adolescent smoking prevention program mediators: a mediated moderation analysis. pp. 1099-1107.

128. Wasserman D, Carli V, Wasserman C, Apter A, Balazs J, et al. (2010) Saving and empowering young lives in Europe (SEYLE): a randomized controlled trial. pp. 192.

129. Jemmott Iii JB, Jemmott LS, O'Leary A, Ngwane Z, Icard L, et al. (2011) Cognitive-behavioural health-promotion intervention increases fruit and vegetable consumption and physical activity among south african adolescents: a cluster-randomised controlled trial. Psychology & Health 26: 167-185 119p.

130. Ertl V, Pfeiffer A, Schauer E, Elbert T, Neuner F (2011) Community-implemented trauma therapy for former child soldiers in Northern Uganda: a randomized controlled trial. pp. 503-512.

131. Hallfors D, Cho H, Rusakaniko S, Iritani B, Mapfumo J, et al. (2011) Supporting adolescent orphan girls to stay in school as HIV risk prevention: evidence from a randomized controlled trial in Zimbabwe. pp. 1082-1088.

132. Tibbits MK, Smith EA, Caldwell LL, Flisher AJ (2011) Impact of HealthWise South Africa on polydrug use and high-risk sexual behavior. pp. 653-663.

133. Çiftçi EK, Arikan D (2012) The effect of training administered to working mothers on maternal anxiety levels and breastfeeding habits. Journal of Clinical Nursing 21: 2170-2178 2179p.

134. Rotheram-Borus MJ, Lightfoot M, Kasirye R, Desmond K (2012) Vocational training with HIV prevention for Ugandan youth. pp. 1133-1137.

135. Ulla Díez SM, Fortis AP, Franco SF (2012) Efficacy of a health-promotion intervention for college students: a randomized controlled trial. pp. 121-132.

136. Smith Fawzi MC, Eustache E, Oswald C, Louis E, Surkan PJ, et al. (2012) Psychosocial support intervention for HIV-affected families in Haiti: Implications for programs and policies for orphans and vulnerable children. Social Science and Medicine 74: 1494-1503.

137. Shahrivar Z, Alaghband-rad J, Gharaie JM, Seddigh A, Salesian N, et al. (2012) The efficacy of an integrated treatment in comparison with treatment as usual in a group of children and adolescents with first-episode psychosis during a two -year follow-up. Iranian Journal of Psychiatry and Clinical Psychology 18: 124-127.

138. Botha CJ, Wild LG (2013) Evaluation of a school-based intervention programme for South African children of divorce. Journal of Child & Adolescent Mental Health 25: 81-91 11p.

139. Mayurachat K, Warunee F, Jutamas C, Patcharaporn A, Kennedy C (2013) An Intervention Study of Changing Eating Behaviors and Reducing Weight in Thai Children Aged 10-12. Pacific Rim International Journal of Nursing Research 17: 317-328 312p.

140. Bieri FA, Gray DJ, Williams GM, Raso G, Li YS, et al. (2013) Health-education package to prevent worm infections in Chinese schoolchildren. pp. 1603-1612.

141. Gyorkos TW, Maheu-Giroux M, Blouin B, Casapia M (2013) Impact of health education on soil-transmitted helminth infections in schoolchildren of the Peruvian Amazon: a cluster-randomized controlled trial. pp. e2397.

142. Mersal FA, Esmat OM, Khalil GM (2013) Effect of prenatal counselling on compliance and outcomes of teenage pregnancy. pp. 10-17.

143. Miller T, Hallfors D, Cho H, Luseno W, Waehrer G (2013) Cost-effectiveness of school support for orphan girls to prevent HIV infection in Zimbabwe (Provisional abstract).

144. Safdie M, Lévesque L, González-Casanova I, Salvo D, Islas A, et al. (2013) Promoting healthful diet and physical activity in the Mexican school system for the prevention of obesity in children. pp. 357-373.

145. Tahlil T, Woodman RJ, Coveney J, Ward PR (2013) The impact of education programs on smoking prevention: a randomized controlled trial among 11 to 14 year olds in Aceh, Indonesia. pp. 367.

146. Cunha DB, Souza BdSNd, Pereira RA, Sichieri R (2013) Effectiveness of a Randomized School-Based Intervention Involving Families and Teachers to Prevent Excessive Weight Gain among Adolescents in Brazil. PLoS ONE 8 (2) (no pagination).

147. Barron IG, Abdallah G, Smith P (2013) Randomized Control Trial of a CBT Trauma Recovery Program in Palestinian Schools. Journal of Loss and Trauma 18: 306-321.

148. Poeta LS, Duarte Mde F, Giuliano Ide C, Mota J (2013) Interdisciplinary intervention in obese children and impact on health and quality of life. Jornal de Pediatria 89: 499-504.

149. Rousham EK, Uzaman B, Abbott D, Lee SF, Mithani S, et al. (2013) The effect of a school-based iron intervention on the haemoglobin concentration of school children in north-west Pakistan. European Journal of Clinical Nutrition 67: 1188-1192.

150. Loyalka P, Liu C, Song Y, Yi H, Huang X, et al. (2013) Can information and counseling help students from poor rural areas go to high school? Evidence from China. Journal of Comparative Economics 41: 1012-1025.

151. McMullen J, O'Callaghan P, Shannon C, Black A, Eakin J (2013) Group trauma-focused cognitive-behavioural therapy with former child soldiers and other war-affected boys in the DR Congo: a randomised controlled trial. Journal of Child Psychology and Psychiatry 54: 1231-1241.

152. Andrade S, Lachat C, Ochoa-Aviles A, Verstraeten R, Huybregts L, et al. (2014) A school-based intervention improves physical fitness in Ecuadorian adolescents: a cluster-randomized controlled trial. International Journal of Behavioral Nutrition & Physical Activity 11: 1-27 27p.

153. Xu F, Wang X, Ware RS, Tse LA, Wang Z, et al. (2014) A school-based comprehensive lifestyle intervention among Chinese kids against Obesity (CLICK-Obesity) in Nanjing City, China: the baseline data. Asia Pacific Journal of Clinical Nutrition 23: 48-54 47p.

154. Betancourt TS, McBain R, Newnham EA, Akinsulure-Smith AM, Brennan RT, et al. (2014) A behavioral intervention for war-affected youth in Sierra Leone: a randomized controlled trial. pp. 1288-1297.

155. Bhana A, Mellins CA, Petersen I, Alicea S, Myeza N, et al. (2014) The VUKA family program: Piloting a family-based psychosocial intervention to promote health and mental health among HIV infected early adolescents in South Africa. pp. 1-11.

156. Habib-Mourad C, Ghandour L, Moore H, Hwalla N, Summerbell C (2014) An intervention to promote Healthy Eating and Physical Activity in Lebanese School children: Health-E-PALS, a pilot cluster randomised controlled trial. pp. 158-159.

157. Hawal NP, Kulkarni N, Shivaswamy, Mallapur, Naik VA (2014) Evaluation of a school based health education model on malaria: A randomized control trial. pp. 105-111.

158. Kapadia-Kundu N, Storey D, Safi B, Trivedi G, Tupe R, et al. (2014) Seeds of prevention: the impact on health behaviors of young adolescent girls in Uttar Pradesh, India, a cluster randomized control trial. pp. 169-179.

159. Marsiglia FF, Booth JM, Ayers SL, Nuño-Gutierrez BL, Kulis S, et al. (2014) Short-term effects on substance use of the keepin' it real pilot prevention program: linguistically adapted for youth in Jalisco, Mexico. pp. 694-704.

160. Richards J, Foster C, Townsend N, Bauman A (2014) Physical fitness and mental health impact of a sport-for-development intervention in a post-conflict setting: randomised controlled trial nested within an observational study of adolescents in Gulu, Uganda. pp. 619.

161. Sankaranarayanan A, Cycil C (2014) Resiliency training in Indian children: a pilot investigation of the Penn Resiliency Program. pp. 4125-4139.

162. Taylor M, Jinabhai C, Dlamini S, Sathiparsad R, Eggers MS, et al. (2014) Effects of a teenage pregnancy prevention program in KwaZulu-Natal, South Africa. pp. 845-858.

163. Viswanathan V, Rengarajan M, Aravindalo Chanan V, Ahmed Khan B, Kuppusamy S, et al. (2014) Positive impact of structured behavior intervention on childhood obesity-Chennai slim and fit program. pp. A317.

164. Dunbar MS, Kang Dufour MS, Lambdin B, Mudekunye-Mahaka I, Nhamo D, et al. (2014) The SHAZ! project: Results from a pilot randomized trial of a structural intervention to prevent HIV among adolescent women in Zimbabwe. PLoS ONE 9 (11) (no pagination).

165. O'Donnell K, Dorsey S, Gong W, Ostermann J, Whetten R, et al. (2014) Treating maladaptive grief and posttraumatic stress symptoms in orphaned children in Tanzania: group-based trauma-focused cognitive-behavioral therapy. Journal of traumatic stress 27: 664-671.

166. Tol WA, Komproe IH, Jordans MJD, Ndayisaba A, Ntamutumba P, et al. (2014) School-based mental health intervention for children in war-affected Burundi: A cluster randomized trial. BMC medicine 12 (1) (no pagination).

167. Hinerman KM, Hull DM, Hayes D, Powell MG, Ferguson S, et al. (2014) Childhood Resiliency Effects from Schoolwide Treatment: A Cluster Randomized Trial. Society for Research on Educational Effectiveness.

168. Irct2014080618713N, Islamic Azad University of Central T (2014) Effect of exercise training in inactive adolescent girls.

169. Theriault FL, Maheu-Giroux M, Blouin B, Casapia M, Gyorkos TW (2014) Effects of a Post-Deworming Health Hygiene Education Intervention on Absenteeism in School-Age Children of the Peruvian Amazon. PLoS Neglected Tropical Diseases 8.

170. Namisi F, Aarø L, Kaaya S, Kajula L, Kilonzo G, et al. (2015) Adolescents' Communication with Parents, Other Adult Family Members and Teachers on Sexuality: Effects of School-Based Interventions in South Africa and Tanzania. AIDS & Behavior 19: 2162-2176 2115p.

171. O'Callaghan P, McMullen J, Shannon C, Rafferty H (2015) Comparing a trauma focused and non trauma focused intervention with war affected Congolese youth: a preliminary randomised trial. Intervention (15718883) 13: 28-44 17p.

172. Tahlil T, Woodman RJ, Coveney J, Ward PR (2015) Six-months follow-up of a cluster randomized trial of school-based smoking prevention education programs in Aceh, Indonesia. BMC public health 15: 1-10 10p.

173. Chen Y, Ma L, Ma Y, Wang H, Luo J, et al. (2015) A national school-based health lifestyles interventions among Chinese children and adolescents against obesity: rationale, design and methodology of a randomized controlled trial in China. pp. 210.

174. Hallfors DD, Cho H, Rusakaniko S, Mapfumo J, Iritani B, et al. (2015) The impact of school subsidies on HIV-related outcomes among adolescent female orphans. pp. 79-84.

175. He FJ, Wu Y, Feng XX, Ma J, Ma Y, et al. (2015) School based education programme to reduce salt intake in children and their families (School-EduSalt): cluster randomised controlled trial. pp. h770.

176. Leme AC, Philippi ST (2015) The "Healthy Habits, Healthy Girls" randomized controlled trial for girls: study design, protocol, and baseline results. pp. 1381-1394.

177. Leventhal KS, Gillham J, DeMaria L, Andrew G, Peabody J, et al. (2015) Building psychosocial assets and wellbeing among adolescent girls: A randomized controlled trial. pp. 284-295.

178. McBain RK, Salhi C, Hann K, Kellie J, Kamara A, et al. (2015) Improving outcomes for caregivers through treatment of young people affected by war: A randomized controlled trial in Sierra Leone. pp. 834-841.

179. Minossi V, Pellanda LC (2015) The "Happy Heart" educational program for changes in health habits in children and their families: Protocol for a randomized clinical trial.

180. Saraf DS, Gupta SK, Pandav CS, Nongkinrih B, Kapoor SK, et al. (2015) Effectiveness of a School Based Intervention for Prevention of Non-communicable Diseases in Middle School Children of Rural North India: A Randomized Controlled Trial. pp. 354-362.

181. Diab M, Peltonen K, Qouta SR, Palosaari E, Punamaki RL (2015) Effectiveness of psychosocial intervention enhancing resilience among war-affected children and the moderating role of family factors. Child Abuse and Neglect 40: 24-35.

182. Gray DJ, Williams G, Bieri F, Li Y, Du ZW, et al. (2015) The 'Magic Glasses'research programme for the global control of intestinal worms. Tropical Medicine and International Health 20: 5.

183. Luseno W, Zhang L, Rusakaniko S, Cho H, Hallfors D (2015) HIV infection and related risk behaviors: Does school support level the playing field between orphans and nonorphans in Zimbabwe? AIDS Care - Psychological and Socio-Medical Aspects of AIDS/HIV 27: 1191-1195.

184. Xu F, Ware RS, Leslie E, Tse LA, Wang Z, et al. (2015) Effectiveness of a randomized controlled lifestyle intervention to prevent obesity among Chinese primary school students: Click-obesity study. PLoS ONE 10 (10) (no pagination).

185. Irct2014051017639N, Vice chancellor for research IUoMS (2015) Nutrition education among obese adolescents.

186. Irct2015070623089N, Vice Chancellor for Research TUoMS (2015) The impact of training programs on reproductive health behavior and its determinants in students.

187. do Nascimento MO, De Micheli D (2015) Evaluation of different school-based preventive interventions for reducing the use of psychotropic substances among students: a randomized study. Ciencia & Saude Coletiva 20: 2499-2510.

188. Yi H, Zhang H, Ma X, Zhang L, Congdon NG, et al. (2015) Impact of a Teacher Incentive on Children's Use of Eyeglasses: A Cluster-Randomized Controlled Trial. Investigative ophthalmology & visual science 56.

189. Gelli A, Masset E, Folson G, Kusi A, Arhinful DK, et al. (2016) Evaluation of alternative school feeding models on nutrition, education, agriculture and other social outcomes in Ghana: Rationale, randomised design and baseline data. Trials 17 (1) (no pagination).

190. Andrade S, Lachat C, Cardon G, Ochoa-Aviles A, Verstraeten R, et al. (2016) Two years of school-based intervention program could improve the physical fitness among Ecuadorian adolescents at health risk: subgroups analysis from a cluster-randomized trial. BMC Pediatrics 16.

191. Erismann S, Shrestha A, Diagbouga S, Knoblauch A, Gerold J, et al. (2016) Complementary school garden, nutrition, water, sanitation and hygiene interventions to improve children's nutrition and health status in Burkina Faso and Nepal: a study protocol. BMC public health 16.

192. Zhao CH, Qiu HS, Qiu HX (2006) Interventions to prevent accidental injuries in children between 7 and 13 years of age. [Chinese]. Chinese Journal of Contemporary Pediatrics 8: 331-333.

193. Barros T, Barreto D, Perez F, Santander R, Yepez E, et al. (2001) A model for the primary prevention of sexually transmitted diseases and HIV/AIDS among adolescents. [Spanish]. pp. 86-94.

194. Duflo E (2007) Education and HIV/AIDS prevention: evidence from a randomized evaluation in Western Kenya. (Background Paper to the 2007 World Development Report WPS4024).

195. Lanerolle P, Atukorala S, de Silva G, Samarasinghe S, Dharmawardena L (2000) Evaluation of nutrition education for improving iron status in combination with daily iron supplementation. Food and Nutrition Bulletin 21: 259-269.

196. Shamah Levy T, Morales Ruán C, Amaya Castellanos C, Salazar Coronel A, Jiménez Aguilar A, et al. (2012) Effectiveness of a diet and physical activity promotion strategy on the prevention of obesity in Mexican school children. pp. 152.

197. Abdulmalik J, Ani C, Ajuwon AJ, Omigbodun O (2016) Effects of problem-solving interventions on aggressive behaviours among primary school pupils in Ibadan, Nigeria. Child and Adolescent Psychiatry and Mental Health 10 (1) (no pagination).

198. Baiocchi M, Omondi B, Langat N, Boothroyd DB, Sinclair J, et al. (2016) A Behavior-Based Intervention That Prevents Sexual Assault: the Results of a Matched-Pairs, Cluster-Randomized Study in Nairobi, Kenya. Prevention science : the official journal of the Society for Prevention Research.

199. Cao ZJ, Wang SM, Chen Y (2017) A randomized trial of multiple interventions for childhood obesity in China. American journal of preventive medicine. pp. 552-560.

200. Cunha CTd, Poblacion AP, Colugnati FAB, Taddei JAdAC, Bracco MM (2016) Effect of an Educational Program on Schoolchildren's Energy Expenditure during Physical Education Classes DURANTE AS AULAS DE EDUCAÇÃO FÍSICA. MedicalExpress 3.

201. Gazzinelli MF, Lobato L, Andrade G, Matoso LF, Diemert DJ, et al. (2016) Improving the understanding of schistosomiasis among adolescents in endemic areas in Brazil: A comparison of educational methods. Patient Education and Counseling 99: 1657-1662.

202. Hidayanty H, Bardosono S, Khusun H, Damayanti R, Kolopaking R (2016) A social cognitive theory-based programme for eating patterns and sedentary activity among overweight adolescents in Makassar, South Sulawesi: a cluster randomised controlled trial. Asia Pacific Journal of Clinical Nutrition 25: S83-S92.

203. Leventhal KS, DeMaria LM, Gillham JE, Andrew G, Peabody J, et al. (2016) A psychosocial resilience curriculum provides the "missing piece" to boost adolescent physical health: A randomized controlled trial of Girls First in India. Social Science and Medicine 161: 37-46.

204. Li X, Harrison SE, Fairchild AJ, Chi P, Zhao J, et al. (2017) A randomized controlled trial of a resilience-based intervention on psychosocial well-being of children affected by HIV/AIDS: Effects at 6- and 12-month follow-up. Social Science and Medicine 16.

205. Liu J, Liu S, Yan J, Lee E, Mayes L (2016) The Impact of Life Skills Training on Behavior Problems in Left-Behind Children in Rural China: A Pilot Study. School Psychology International 37: 73-84.

206. Mushtaq A, Lochman JE, Tariq PN, Sabih F (2016) Preliminary Effectiveness Study of Coping Power Program for Aggressive Children in Pakistan. Prevention science : the official journal of the Society for Prevention Research.

207. Nayak BS, Bhat VH (2016) School based multicomponent intervention for obese children in Udupi district, South India - A randomized controlled trial. Journal of Clinical and Diagnostic Research 10: SC24-SC28.

208. Shen J, Pang S, Schwebel DC (2016) Evaluation of a drowning prevention program based on testimonial videos: A randomized controlled trial. Journal of Pediatric Psychology 41: 555-565.

209. Thakur JS, Bharti B, Tripathy JP, Dhawan V, Bhansali A (2017) Impact of 20 week lifestyle intervention package on anthropometric biochemical and behavioral characteristics of schoolchildren in North India. Journal of tropical pediatrics. pp. 368-376.

210. Zuilkowski SS, Collet K, Jambai M, Akinsulure-Smith AM, Betancourt TS (2016) Youth and Resilience in Postconflict Settings: An Intervention for War-Affected Youth in Sierra Leone. Human Development 59: 64-80.

211. Zirembuzi GW, Mugero C Diarrhoea case management experience from a specialised unit. 215 p.-215 p.

212. Stelmach W, Korzeniewska A, Jerzycska J, Majak P, Stelmach P, et al. (2008) Asthma education program and asthma symptoms, activity, and emotions in children. International Review of Allergology and Clinical Immunology 14: 22-27.

213. Wu JZ, Dai MH, Xiong J, Liu HX (2016) Effect of motivational interviewing combined with peer participation on obesity management in adolescents. [Chinese]. Chinese Journal of Contemporary Pediatrics 18: 645-649.

214. Bracken PJ, Giller JR, Ssekiwanuka JK The rehabilitation of child soldiers: defining needs and appropriate responses. Med-Confl-Surviv 12: 114-115.

215. Horizons Evaluating the Kenya girl guides association&#039;s HIV/AIDS Peer education program for younger youth: Baseline results. 10 p.-10 p.

216. Perry CL, Grant M A cross-cultural pilot study on alcohol education and young people. World health statistics quarterly 44: 70-73.

217. Visser MJ HIV/AIDS Prevention through Peer Education and Support in Secondary Schools in South Africa. SAHARA Journal of Social Aspects of HIV/AIDS/Journal des Aspects Sociaux du VIH/SIDA 4: 678-694, fig.

218. Iyengar SD, Grover A, Kumar R, Ganguly NK, Anand IS, et al. (1991) A rheumatic fever and rheumatic heart disease control programme in a rural community of north india. pp. 268-271.

219. Schall VT, Dias AGP, Malaquias MLG, Gomes Dos Santos M (1993) Health education in first level schools at the outskirts of Belo Horizonte, Minas Gerais State, Brazil: I. Evaluation of a health education program on schistosomiasis. Revista do Instituto de Medicina Tropical de Sao Paulo 35: 563-572.

220. Locklin MP (1995) Telling the world: low income women and their breastfeeding experiences. Journal of Human Lactation 11: 285-291 287p.

221. Briggs LA (1998) 'Contraceptive counselling amongst adolescents in Port Harcourt, Nigeria: social welfare workers can help too'. West African Journal of Nursing 9: 84-87 84p.

222. Martini JG, Gregis C, Jardim L (1999) Teenage pregnancy: from disciplinary practice to a pedagogy commited [sic] to freedom. Revista brasileira de enfermagem 52: 539-546 538p.

223. Kim J, Alderman H, Orazem PF (1999) Can Private School Subsidies Increase Enrollment for the Poor? The Quetta Urban Fellowship Program. pp. 443-465.

224. Campbell C, Mzaidume Z (2001) Grassroots participation, peer education, and HIV prevention by sex workers in South Africa. American Journal of Public Health 91: 1978-1986 1979p.

225. Dale N, Danko R, Breen M (2001) Confronting adolescent bias and intolerance through cross-cultural immersion: an American-Croatian collaboration. Child Welfare 80: 623-630 628p.

226. Campbell C, MacPhail C (2002) Peer education, gender and the development of critical consciousness: participatory HIV prevention by South African youth. Social Science & Medicine 55: 331-345 315p.

227. Lansdown R, Ledward A, Hall A, Issae W, Yona E, et al. (2002) Schistosomiasis, helminth infection and health education in Tanzania: achieving behaviour change in primary schools. pp. 425-433.

228. Wolf RC, Bond KC (2002) Exploring similarity between peer educators and their contacts and AIDS-protective behaviours in reproductive health programmes for adolescents and young adults in Ghana. AIDS care 14: 361-373.

229. Bhuiya A, Chowdhury M (2002) Beneficial effects of a woman-focused development programme on child survival: evidence from rural Bangladesh. Social Science & Medicine 55: 1553-1560.

230. Fang X, Li X, Stanton B, Dong Q (2003) Social network positions and smoking experimentation among Chinese adolescents. American journal of health behavior 27: 257-267 211p.

231. Maluleke TX (2003) Improving the health status of women through puberty rites for girls. Health SA Gesondheid 8: 68-73 66p.

232. Jindal-Snape D (2004) Generalization and maintenance of social skills of children with visual impairments: self-evaluation and the role of feedback. Journal of Visual Impairment & Blindness 98: 470-483 414p.

233. Jordan CM, Lee PA, Hampton R, Pirie PL (2004) Recommendations from lead poisoning prevention program participants: best practices. Health promotion practice 5: 429-437 429p.

234. Campbell S (2005) Using peer education projects to prevent HIV/AIDS in young people. Nursing Standard 20: 50-55 56p.

235. Daenseekaew S, Srisontisuk S, Thongkrajar E, Sriruecha P (2005) Mobilizing communities to combat illicit drug use in Northeast Thailand. Thai Journal of Nursing Research 9: 141-154 114p.

236. Fongkaew W, Rutchanagul P, Fongkaew K (2005) Linking sexual and reproductive health to HIV/AIDS prevention among Thai early adolescents: youth and adult partnership approaches. Thai Journal of Nursing Research 9: 251-267 217p.

237. Jindal-Snape D (2005) Use of feedback from sighted peers in promoting social interaction skills. Journal of Visual Impairment & Blindness 99: 403-412 410p.

238. Vásquez ML, Argote LA, Castillo E, Mejía ME, Tunjo R, et al. (2005) Education and sexual and reproductive rights: a comprehensive perspective with adolescents in school. Colombia Medica 36: 6-13 18p.

239. Magnani R, MacIntyre K, Karim AM, Brown L, Hutchinson P, et al. (2005) The impact of life skills education on adolescent sexual risk behaviors in KwaZulu-Natal, South Africa. Journal of Adolescent Health 36: 289-304.

240. Sorensen G, Gupta PC, Sinha DN, Shastri S, Kamat M, et al. (2005) Teacher tobacco use and tobacco use prevention in two regions in India: results of the Global School Personnel Survey. Preventive Medicine 41: 417-423.

241. Amoo PO, Bakare P, Ajibade BL (2006) Teaching styles as determinants of learning outcome among student nurses in Oyo State. West African Journal of Nursing 17: 110-115 116p.

242. Schuler SR, Bates LM, Islam F, Islam MK (2006) The timing of marriage and childbearing among rural families in Bangladesh: choosing between competing risks. Social Science & Medicine 62: 2826-2837 2812p.

243. Cartagena RG, Veugelers PJ, Kipp W, Magigav K, Laing LM (2006) Effectiveness of an HIV Prevention Program for Secondary School Students in Mongolia. pp. 925.e929-925.e916.

244. Allen D (2007) Malnutrition in low income countries: lessons from the field. Paediatric Nursing 19: 20-21 22p.

245. Kaponda CPN, Dancy BL, Norr KF, Kachingwe SI, Mbeba MM, et al. (2007) Research brief: community consultation to develop an acceptable and effective adolescent HIV prevention intervention. JANAC: Journal of the Association of Nurses in AIDS Care 18: 72-77 76p.

246. Murakami JK, Filho JFP, Filho PCP, Acorinte AC, Napoleao AA (2007) Planning, developing, and evaluating a group intervention with adolescents: a systemic perspective. Revista Eletronica de Enfermagem 9: 772-783 712p.

247. Gungoren B, Latipov R, Regallet G, Musabaev E (2007) Effect of hygiene promotion on the risk of reinfection rate of intestinal parasites in children in rural Uzbekistan. pp. 564-569.

248. Maldonado JC, Meléndez SD, Figueras A (2007) Long-term effects of an educational intervention on self-medication and appropriate drug use in single-sex secondary public schools, Quito, Ecuador. pp. 92-99.

249. Rozanov VB, Alexandrov AA, Shugaeva EN, Perova NV, Maslennikova GY, et al. (2007) Primary prevention of cardiovascular diseases: Long term results of five year long preventive intervention in 12-year old boys (ten year prospective study). Kardiologiya 47: 60-68.

250. Carvalho QCM, Cardoso MVL, da Silva MJ, Braga VAB, Galvão MTG (2008) Violence against children and adolescents: reflective study on public policies. Revista da Rede de Enfermagem do Nordeste 9: 157-164 158p.

251. Tu X, Lou C, Gao E, Shah IH (2008) Long-term effects of a community-based program on contraceptive use among sexually active unmarried youth in Shanghai, China. Journal of Adolescent Health 42: 249-258 210p.

252. Shooshtary MH, Panaghi L, Moghadam JA (2008) Outcome of cognitive behavioral therapy in adolescents after natural disaster. pp. 466-472.

253. Lidia Nuno-Gutierrez B, Alvarez-Nemegyei J, Madrigal-de Leon EA (2008) Effect of an anti-tobacco intervention in high school students from Guadalajara, Mexico. Salud Mental 31: 181-188.

254. Cluver L (2009) Peer group support intervention reduces psychological distress in AIDS orphans. Evidence Based Mental Health 12: 120-120 121p.

255. King R, Lloyd C, Clune A, Allan R (2009) The experience of being a peer outreach volunteer: benefits and challenges. Australian e-Journal for the Advancement of Mental Health 8: 1-11 11p.

256. Nor B, Zembe Y, Daniels K, Doherty T, Jackson D, et al. (2009) 'Peer but not peer': Considering the context of infant feeding peer counseling in a high HIV prevalence area. Journal of Human Lactation 25: 427-434 428p.

257. Simba DO, Kakoko DC (2009) Volunteerism among out-of-school adolescent reproductive health peer educators: is it a sustainable strategy in resource constrained countries? African journal of reproductive health 13: 99-110 112p.

258. Mukoma W, Flisher AJ, Ahmed N, Jansen S, Mathews C, et al. (2009) Process evaluation of a school-based HIV/AIDS intervention in South Africa. pp. 37-47.

259. Alaofe H, Zee J, Dossa R, O'Brien HT (2009) Effect of a nutrition education program and diet modification in beninese adolescent girls suffering from mild iron deficiency anemia. Ecology of Food and Nutrition 48: 21-38.

260. Trofor A, Mihaltan F, Mihaicuta S, Lotrean L (2009) Smoking cessation and prevention for young people--Romanian expertise. Pneumologia (Bucharest, Romania) 58: 72-78.

261. Arevian M (2010) Training trainees, young activists, to conduct awareness campaigns about prevention of substance abuse among Lebanese/Armenian young people. Journal of Interprofessional Care 24: 173-182 110p.

262. Goenka S, Tewari A, Arora M, Stigler MH, Perry CL, et al. (2010) Process evaluation of a tobacco prevention program in Indian schools--methods, results and lessons learnt. Health education research 25: 917-935 919p.

263. Nankunda J, Tumwine JK, Nankabirwa V, Tylleskär T (2010) 'She would sit with me': mothers' experiences of individual peer support for exclusive breastfeeding in Uganda. International Breastfeeding Journal 5: 13p-13p 11p.

264. Das P, Pal R, Pal S (2010) Awareness on psychosomatic health among adolescent girls of three schools in north Kolkata. pp. 355-359.

265. Schwarzer R, Cao DS, Lippke S (2010) Stage-matched minimal interventions to enhance physical activity in Chinese adolescents. pp. 533-539.

266. Tol WA, Komproe IH, Jordans MJ, Gross AL, Susanty D, et al. (2010) Mediators and moderators of a psychosocial intervention for children affected by political violence. pp. 818-828.

267. Qin JP, Chen S, Di N, Yang YP, Zhou L, et al. (2010) [Evaluation of the effect of post-abortion counseling and education among unmarried abortion adolescents]. [Chinese]. Zhonghua fu chan ke za zhi 45: 201-204.

268. Kennedy C (2010) Positive prevention, serosorting, and a matrimonial service for people living with HIV in Chennai, India. Dissertation Abstracts International: Section B: The Sciences and Engineering 71: 258.

269. Ayi I, Nonaka D, Adjovu JK, Hanafusa S, Jimba M, et al. (2010) School-based participatory health education for malaria control in Ghana: engaging children as health messengers. Malaria Journal 9.

270. Beretta MIR, de Freitas MA, Dupas G, Fabbro MRC, Ruggiero EMS (2011) The construction of a Project in adolescent maternity: an experience report. Revista Da Escola de Enfermagem Da Usp 45: 533-536 534p.

271. de MendonÃ§a FigueirÃªdo Coelho Coelho M, CorrÃªa Lima Miranda K, Taciana Firmino Bezerra S, VilanÃ­ Cavalcante Guedes MG, Leite Cabral R, et al. (2011) 'Papo Irado': technology of popular health education with adolescents. Revista de Atencao Primaria a Saude 14: 502-506 505p.

272. Dehghan-Nayeri N, Adib-Hajbaghery M (2011) Effects of progressive relaxation on anxiety and quality of life in female students: A non-randomized controlled trial. Complementary Therapies in Medicine 19: 194-200 197p.

273. Rabello N, Anderson MIP (2011) Eating habits and physical activity in school children: report of a health education experience. Revista de Atencao Primaria a Saude 14: 239-249 211p.

274. Rijsdijk LE, Bos AE, Ruiter RA, Leerlooijer JN, Haas B, et al. (2011) The World Starts With Me: A multilevel evaluation of a comprehensive sex education programme targeting adolescents in Uganda. pp. 334.

275. Acemoglu H, Palanci Y, Set T, Vancelik S, Isik M, et al. (2011) An intervention study for viral hepatitis: Peer-led health education among high school students. Saudi Medical Journal 32: 183-187.

276. Scarcella P, Buonomo E, Zimba I, Doro Altan AM, Germano P, et al. (2011) The impact of integrating food supplementation, nutritional education and HAART (Highly Active Antiretroviral Therapy) on the nutritional status of patients living with HIV/AIDS in Mozambique: results from the DREAM Programme. Igiene e sanita pubblica 67: 41-52.

277. Burke, H M, Pedersen, K F, Williamson, et al. (2012) An assessment of cost, quality and outcomes for five HIV prevention youth peer education programs in Zambia (Provisional abstract). pp. 359-369.

278. Ismayilova L, Ssewamala FM, Karimli L (2012) Family support as a mediator of change in sexual risk-taking attitudes among orphaned adolescents in rural Uganda. pp. 228-235.

279. Ndebele M, Kasese-Hara M, Greyling M (2012) Application of the information, motivation and behavioural skills model for targeting HIV risk behaviour amongst adolescent learners in South Africa. pp. S37-47.

280. Pitt C, Diawara H, Ouedraogo DJ, Diarra S, Kabore H, et al. (2012) Intermittent preventive treatment of malaria in children: A qualitative study of community perceptions and recommendations in Burkina Faso and Mali.

281. Montgomery P, Ryus CR, Dolan CS, Dopson S, Scott LM (2012) Sanitary pad interventions for girls' education in Ghana: a pilot study. PLoS ONE [Electronic Resource] 7: e48274.

282. Dadashzadeh H, Yazdandoost R, Gharraee B, Farid AA (2012) Effectiveness of cognitive-behavioral group therapy and exposure therapy on interpretation bias and fear of negative evaluation in social anxiety disorder. Iranian Journal of Psychiatry and Clinical Psychology 18: 40-51.

283. Jimerson SR, Nickerson AB, Mayer MJ, Furlong MJ (2012) Handbook of school violence and school safety: International research and practice (2nd ed.). 645.

284. Gazzinelli MF, de Souza V, Lobato de Araujo LH, Costa RdM, Soares AN, et al. (2012) Theater in the education of children and teenagers participating in a clinical trial. Revista de Saude Publica 46: 999-1006.

285. Kaushik U, Shrivastav R, Arora M, Reddy KS (2012) Scaling up a successful school based tobacco use prevention program in India: from efficacy to effectiveness. Circulation 125: E898-E898.

286. D'Souza RP, Renuka N (2013) A Study to Evaluate the effectiveness of Child to Child Programme on Road Safety Measures among Primary School Children at Selected School, Bangalore. International Journal of Nursing Education 5: 165-167 163p.

287. McCreary LL, Kaponda CPN, Davis K, Kalengamaliro M, Norr KF (2013) Empowering Peer Group Leaders for HIV Prevention in Malawi. Journal of Nursing Scholarship 45: 288-297 210p.

288. Zack B, Smith C, Andrews MC, May JP (2013) Peer Health Education in Haiti’s National Penitentiary: The “Health through Walls” Experience. Journal of Correctional Health Care 19: 65-68 64p.

289. Letona P, Gittelsohn J, Chacon V, Ramirez-Zea M, Caballero B (2013) Process evaluation of a community-based pilot intervention for chronic disease prevention in Guatemalan school-aged children.

290. Tewari A, Bassi S, Stigler MH, Arora M, Perry C, et al. (2013) Innovative intervention strategy for tobacco control in low SES communities in Delhi: Project activity. pp. S17.

291. Yusuf FA (2013) Effects of peer modelling technique in reducing substance abuse among undergraduates in Osun State, Nigeria. IFE Psychologia: An International Journal 21: 194-205.

292. Daivadanam M, Absetz P, Sathish T, Thankappan KR, Fisher EB, et al. (2013) Lifestyle change in Kerala, India: needs assessment and planning for a community-based diabetes prevention trial. BMC public health 13.

293. Michielsen K, Temmerman M, Van Rossem R (2013) Limited effectiveness of HIV prevention for young people in sub-Saharan Africa: studying the role of intervention and evaluation. Facts, views & vision in ObGyn 5: 196-208.

294. Callister LC (2014) Fostering Hope: Documenting Interventions Targeting Ethiopian Child Brides. MCN: The American Journal of Maternal Child Nursing 39: 335-335 331p.

295. Liliana Muñoz Ortega M, Carolina Barbosa Ramírez L, Méndez Heilman M, del Pilar Cardona Gómez G, Fernanda Ruiz Eslava L (2014) 'Take Control', a Prevention Program for Problem Drinking in University Communities. Investigacion en Enfermeria: Imagen y Desarrollo 16: 27-48 22p.

296. Pereira DC, da Silva EKA, Ito CY, Bell BB, Ribeiro CMG, et al. (2014) Culinary workshop as a strategy for Occupational Therapy intervention with adolescents in situation of social vulnerability. Cadernos de Terapia Ocupacional da UFSCar 22: 621-626 626p.

297. Jemmott LS, Jemmott JB, Ngwane Z, Icard L, O'Leary A, et al. (2014) 'Let Us Protect Our Future' a culturally congruent evidenced-based HIV/STD risk-reduction intervention for young South African adolescents. pp. 166-181.

298. Miljković S, Baljozović D, Krajnović D, Tasić L, Sbutega-Milosević G (2014) The impact of education on adolescents' sun behavior: experiences from Serbia. pp. 330-336.

299. Sarnquist C, Omondi B, Sinclair J, Gitau C, Paiva L, et al. (2014) Rape prevention through empowerment of adolescent girls. pp. e1226-1232.

300. Li XH, Lin S, Guo H, Huang Y, Wu L, et al. (2014) Effectiveness of a school-based physical activity intervention on obesity in school children: a nonrandomized controlled trial. BMC public health 14: 1282.

301. Fatti G, Shaikh N, Eley B, Grimwood A (2014) Improved virological suppression in children on antiretroviral treatment receiving community-based adherence support: a multicentre cohort study from South Africa. AIDS care 26: 448-453.

302. Leerlooijer JN, Kok G, Weyusya J, Bos AER, Ruiter RAC, et al. (2014) Applying Intervention Mapping to develop a community-based intervention aimed at improved psychological and social well-being of unmarried teenage mothers in Uganda. Health education research 29: 598-610.

303. Menacho L, Galea J, Young S (2015) Feasibility of Recruiting Peer Educators to Promote HIV Testing Using Facebook Among Men Who have Sex with Men in Peru. AIDS & Behavior 19: 123-129 127p.

304. Pridmore P, Carr-Hill R, Amuyunzu-Nyamongo M, Lang'o D, McCowan T, et al. (2015) Tackling the urban health divide though enabling intersectoral action on malnutrition in Chile and Kenya. pp. 313-321.

305. Shen M, Hu M, Sun Z (2015) Assessment of school-based quasi-experimental nutrition and food safety health education for primary school students in two poverty-stricken counties of West China.

306. Sorsdahl K, Myers B, Ward CL, Matzopoulos R, Mtukushe B, et al. (2015) Adapting a blended motivational interviewing and problem-solving intervention to address risky substance use amongst South Africans. Psychotherapy research : journal of the Society for Psychotherapy Research 25: 435-444.

307. Wirtz AL, Trapence G, Jumbe V, Umar E, Ketende S, et al. (2015) Feasibility of a combination HIV prevention program for men who have sex with men in Blantyre, Malawi. Journal of Acquired Immune Deficiency Syndromes 70: 155-162.

308. Diez-Canseco F, Boeren Y, Quispe R, Chiang ML, Miranda JJ (2015) Engagement of adolescents in a health communications program to prevent noncommunicable diseases: Multiplicadores Jovenes, Lima, Peru, 2011. Preventing Chronic Disease 12: E28.

309. Zelikson DI (2015) INFLUENCE OF POSITIVE PSYCHOLOGICAL INTERVENTIONS ON ADOLESCENTS' WELL-BEING. Rossiyskiy Psikhologicheskiy Zhurnal 12: 33-+.

310. Bhave S, Pandit A, Yeravdekar R, Madkaikar V, Chinchwade T, et al. (2016) Effectiveness of a 5-year school-based intervention programme to reduce adiposity and improve fitness and lifestyle in Indian children; the SYM-KEM study. Archives of Disease in Childhood 101: 33-41 39p.

311. Bagnoud FX A Community Based Approaches to Assist Orphans and Vulnerable Children in Luwero, Uganda-Strategies for Implementation. 11 p.-11 p.

312. Watson C AIDS focus. AIDS in Uganda. Nurs Stand 4: 50-52.

313. Mahat G, Scoloveno M (2006) An HIV/AIDS Education Intervention for Nepalese Adolescent Females. Nursing Clinics of North America 41: 409-423.

314. Al-Iryani B, Al-Sakkaf K, Basaleem H, Kok G, Van Den Borne B (2011) Process evaluation of a three-year community-based peer education intervention for HIV prevention among yemeni young people. International Quarterly of Community Health Education 31: 133-154.

315. Falb KL, Annan J, Kpebo D, Cole H, Willie T, et al. (2015) Differential Impacts of an Intimate Partner Violence Prevention Program Based on Child Marriage Status in Rural Cote d'Ivoire. Journal of Adolescent Health 57: 553-558.

316. Iritani BJ, Cho H, Rusakaniko S, Mapfumo J, Hartman S, et al. (2016) Educational Outcomes for Orphan Girls in Rural Zimbabwe: Effects of a School Support Intervention. Health Care for Women International 37: 301-322 322p.

317. Michielsen K, Beauclair R, Delva W, Roelens K, Van Rossem R, et al. (2012) Effectiveness of a peer-led HIV prevention intervention in secondary schools in Rwanda: results from a non-randomized controlled trial. BMC public health 12: 729.

318. Dehayem MY, Takogue R, Choukem SP, Donfack OT, Katte JC, et al. (2016) Impact of a pioneer diabetes camp experience on glycemic control among children and adolescents living with type 1 diabetes in sub-Saharan Africa. BMC Endocrine Disorders 16: 5.

319. Harrison A, Hoffman S, Mantell JE, Smit JA, Leu C-S, et al. (2016) Gender-focused HIV and pregnancy prevention for school-going adolescents: The Mpondombili pilot intervention in KwaZulu-Natal, South Africa. Journal of Hiv-Aids & Social Services 15: 29-47.

320. Miller KS, Cham HJ, Taylor EM, Berrier FL, Duffy M, et al. (2016) Formative Work and Community Engagement Approaches for Implementing an HIV Intervention in Botswana Schools. American Journal of Public Health 106: 1439-1441.

321. Polo-Oteyza E, Ancira-Moreno M, Rosel-Pech C, Sánchez-Mendoza MT, Salinas-Martínez V, et al. (2017) An intervention to promote physical activity in Mexican elementary school students: building public policy to prevent noncommunicable diseases. Nutrition Reviews 75: 70-78.

322. Steffenon Weber IT, Strassburger SZ, Strassburger MJ, Borin Busnello M, Bento Franz LB (2017) ORIGINAL ARTICLE EFFECTS OF A DOMICILIARY NUTRITIONAL EDUCATION PROGRAM FOR ASTHMATIC ADOLESCENTS. Journal of Nursing UFPE / Revista de Enfermagem UFPE 11: 112-120.

323. Van der Heijden I, Swartz S (2010) Bereavement, Silence and Culture within a Peer-Led HIV/AIDS-Prevention Strategy for Vulnerable Children in South Africa. African Journal of AIDS Research 9.

324. Vig J, Miller KS, Chirwa-Motswere C, Winskell K, Stallcup E (2016) Involving parents from the start: Formative evaluation for a large randomised controlled trial with Botswana Junior Secondary School students. African Journal of AIDS Research 15: 9-15.

325. Kabahenda M, Mullis RM, Erhardt JG, Northrop-Clewes C, Nickols SY Nutrition Education to Improve Dietary Intake and Micronutrient Nutriture among Children in Less-Resourced Areas: A Randomised Controlled Intervention in Kabarole District, Western Uganda. South African Journal of Clinical Nutrition 24: 83-88, tab., fig.

326. Haggerty PA, Muladi K, Kirkwood BR, Ashworth A, Manunebo M (1994) Community-based hygiene education to reduce diarrhoeal disease in rural Zaire: impact of the intervention on diarrhoeal morbidity. pp. 1050-1059.

327. Khamhoung K, Bodhisane N, Pathammavong C, Ouenvilay S, Senthavisouk B, et al. (2000) Nutritional status of pre-school children and women in selected villages in the Suvannakhet Province, Lao PDR--an intervention trial. pp. 63-74.

328. Dybdahl R (2001) Children and mothers in war: an outcome study of a psychosocial intervention program. pp. 1214-1230.

329. Bowen A, Ma H, Ou J, Billhimer W, Long T, et al. (2007) A cluster-randomized controlled trial evaluating the effect of a handwashing-promotion program in Chinese primary schools. pp. 1166-1173.

330. Jiang J, Xia X, Greiner T, Wu G, Lian G, et al. (2007) The effects of a 3-year obesity intervention in schoolchildren in Beijing. pp. 641-646.

331. Van Winghem J, Telfer B, Reid T, Ouko J, Mutunga A, et al. (2008) Implementation of a comprehensive program including psycho-social and treatment literacy activities to improve adherence to HIV care and treatment for a pediatric population in Kenya. BMC Pediatrics 8: 52.

332. Toruner EK, Savaser S (2010) A controlled evaluation of a school-based obesity prevention in Turkish school children. pp. 473-482.

333. Draper CE, de Villiers A, Lambert EV, Fourie J, Hill J, et al. (2010) HealthKick: a nutrition and physical activity intervention for primary schools in low-income settings. BMC public health 10: 398.

334. Bos K, Zeanah CH, Fox NA, Drury SS, McLaughlin KA, et al. (2011) Psychiatric outcomes in young children with a history of institutionalization [NCT00747396] [Review]. pp. 15-24.

335. Perez-Morales ME, Bacardi-Gascon M, Jimenez-Cruz A (2011) Long-term randomized school-based intervention: Effect on obesity and lifestyles in Mexico. pp. 74.

336. Bacardí-Gascon M, Pérez-Morales ME, Jiménez-Cruz A (2012) A six month randomized school intervention and an 18-month follow-up intervention to prevent childhood obesity in Mexican elementary schools. Nutricion Hospitalaria 27: 755-762 758p.

337. Lobato L, Miranda A, Faria IM, Bethony JM, Gazzinelli MF (2012) Development of cognitive abilities of children infected with helminths through health education. pp. 514-519.

338. Grimwood A, Fatti G, Mothibi E, Malahlela M, Shea J, et al. (2012) Community adherence support improves programme retention in children on antiretroviral treatment: a multicentre cohort study in South Africa. Journal of the International AIDS Society 15: 17381.

339. Fischer AB, Miller LC, Rogers BL, Lohani M, Singh P, et al. (2012) Child food consumption patterns and diet quality in rural Nepal: Effectiveness of a community development intervention. FASEB journal 26.

340. Alvirde-García U, Rodríguez-Guerrero AJ, Henao-Morán S, Gómez-Pérez FJ, Aguilar-Salinas CA (2013) [Results of a community-based life style intervention program for children]. pp. 406-414.

341. Meng L, Xu H, Liu A, Raaij J, Bemelmans W, et al. (2013) The costs and cost-effectiveness of a school-based comprehensive intervention study on childhood obesity in China (Provisional abstract). pp. e77971.

342. Safdie M, Jennings-Aburto N, Levesque L, Janssen I, Campirano-Nunez F, et al. (2013) Impact of a school-based intervention program on obesity risk factors in Mexican children. Salud Publica de Mexico 55: S374-S387.

343. Mohammadzaheri F, Koegel L, Rezaee M, Rafiee S (2014) A Randomized Clinical Trial Comparison Between Pivotal Response Treatment (PRT) and Structured Applied Behavior Analysis (ABA) Intervention for Children with Autism. Journal of Autism & Developmental Disorders 44: 2769-2777 2769p.

344. Cao Z, Wang S, Zheng W, Guo J, Qu S (2014) [Evaluation on the effectiveness of intervention comprehensive program on child obesity, using Generalized Estimating Equation]. pp. 773-778.

345. Mohammadzaheri F, Koegel L, Rezaei M, Bakhshi E (2015) A Randomized Clinical Trial Comparison Between Pivotal Response Treatment (PRT) and Adult-Driven Applied Behavior Analysis (ABA) Intervention on Disruptive Behaviors in Public School Children with Autism. Journal of Autism & Developmental Disorders 45: 2899-2907 2899p.

346. Maselko J, Sikander S, Bhalotra S, Bangash O, Ganga N, et al. (2015) Effect of an early perinatal depression intervention on long-term child development outcomes: Follow-up of the Thinking Healthy Programme randomised controlled trial. pp. 609-617.

347. Verbestel V, Henauw S, Barba G, Eiben G, Gallois K, et al. (2015) Effectiveness of the IDEFICS intervention on objectively measured physical activity and sedentary time in European children. pp. 57-67.

348. Waswa LM, Jordan I, Herrmann J, Krawinkel MB, Keding GB (2015) Community-based educational intervention improved the diversity of complementary diets in western Kenya: results from a randomized controlled trial. Public Health Nutrition 18: 3406-3419.

349. Cao ZJ, Wang SM, Chen Y (2014) A randomized trial of multiple interventions for childhood obesity in china. pp. 552-560.

350. Lin H, Wang YF, Wu YP (2007) [A control study of a school-based life skills education on prevention of behavior problems in third-grade schoolchildren]. pp. 319-322.

351. Singogo PP Bargirls change behaviour with the introduction of peer group education: Nanjiri Trading Centre, Lilongwe District. Moyo XXV: 19-21.

352. Victora CG, Langer A, Barros F, Belizan J, Farnot U, et al. (1994) The Latin American Multicenter Trial on psychosocial support during pregnancy: methodology and baseline comparability. Latin American Network for Perinatal and Reproductive Research (LANPER). pp. 379-394.

353. Pauw J, Ferrie J, Rivera Villegas R, Medrano Martínez J, Gorter A, et al. (1996) A controlled HIV/AIDS-related health education programme in Managua, Nicaragua. pp. 537-544.

354. Griensven GJ, Limanonda B, Ngaokeow S, Ayuthaya SI, Poshyachinda V (1998) Evaluation of a targeted HIV prevention programme among female commercial sex workers in the south of Thailand. pp. 54-58.

355. Wu Z, Detels R, Zhang J, Li V, Li J (2002) Community-based trial to prevent drug use among youths in Yunnan, China. American Journal of Public Health 92: 1952-1957 1956p.

356. Amirkhanian YA, Kelly JA, Kabakchieva E, McAuliffe TL, Vassileva S (2003) Evaluation of a social network HIV prevention intervention program for young men who have sex with men in Russia and Bulgaria. AIDS Education & Prevention 15: 205-220 216p.

357. Basu I, Jana S, Rotheram-Borus MJ, Swendeman D, Lee SJ, et al. (2004) HIV prevention among sex workers in India. pp. 845-852.

358. Ergene T, Çok F, Tümer A, Ünal S (2005) A controlled-study of preventive effects of peer education and single-session lectures on HIV/AIDS knowledge and attitudes among university students in Turkey. AIDS Education & Prevention 17: 268-278 211p.

359. Feldblum PJ, Hatzell T, Damme K, Nasution M, Rasamindrakotroka A, et al. (2005) Results of a randomised trial of male condom promotion among Madagascar sex workers. pp. 166-173.

360. Morisky DE, Nguyen C, Ang A, Tiglao TV (2005) HIV/AIDS prevention among the male population: results of a peer education program for taxicab and tricycle drivers in the Philippines. pp. 57-68.

361. Wang S, Keats D (2005) Developing an innovative cross-cultural strategy to promote HIV/AIDS prevention in different ethnic cultural groups of China. pp. 874-891.

362. Morisky DE, Stein JA, Chiao C, Ksobiech K, Malow R (2006) Impact of a social influence intervention on condom use and sexually transmitted infections among establishment-based female sex workers in the Philippines: a multilevel analysis. Health Psychology 25: 595-603 599p.

363. Bastani F, Hidarnia A, Montgomery KS, Aguilar-Vafaei ME, Kazemnejad A (2006) Does relaxation education in anxious primigravid Iranian women influence adverse pregnancy outcomes?: a randomized controlled trial. pp. 138-146.

364. Thomsen SC, Ombidi W, Toroitich-Ruto C, Wong EL, O Tucker H, et al. (2006) A prospective study assessing the effects of introducing the female condom in a sex worker population in Mombasa, Kenya. Sexually Transmitted Infections 82: 397-402.

365. Gregson S, Adamson S, Papaya S, Mundondo J, Nyamukapa CA, et al. (2007) Impact and process evaluation of integrated community and clinic-based HIV-1 control: a cluster-randomised trial in eastern Zimbabwe. pp. e102.

366. Hoke TH, Feldblum PJ, Damme KV, Nasution MD, Grey TW, et al. (2007) Randomised controlled trial of alternative male and female condom promotion strategies targeting sex workers in Madagascar. pp. 448-453.

367. Kalichman SC, Cherry C, White D, Pope H, Cain D, et al. (2007) Altering key characteristics of a disseminated effective behavioral intervention for HIV positive adults: the "healthy relationships" experience. pp. 145-153.

368. Pearson CR, Micek MA, Simoni JM, Hoff PD, Matediana E, et al. (2007) Randomized control trial of peer-delivered, modified directly observed therapy for HAART in Mozambique. pp. 238-244.

369. Kerrigan D, Telles P, Torres H, Overs C, Castle C (2008) Community development and HIV/STI-related vulnerability among female sex workers in Rio de Janeiro, Brazil. Health education research 23: 137-145.

370. Latkin CA, Donnell D, Metzger D, Sherman S, Aramrattna A, et al. (2009) The efficacy of a network intervention to reduce HIV risk behaviors among drug users and risk partners in Chiang Mai, Thailand and Philadelphia, USA. Social Science & Medicine 68: 740-748 749p.

371. Stubbs BA, Micek MA, Pfeiffer JT, Montoya P, Gloyd S (2009) Treatment partners and adherence to HAART in Central Mozambique. pp. 1412-1419.

372. Yotebieng M, Turner AN, Hoke TH, Van Damme K, Rasolofomanana JR, et al. (2009) Effect of consistent condom use on 6-month prevalence of bacterial vaginosis varies by baseline BV status. Tropical Medicine and International Health 14: 480-486.

373. Lau JT, Wan SP, Yu XN, Cheng F, Zhang Y, et al. (2009) Changes in condom use behaviours among clients of female sex workers in China. Sexually Transmitted Infections 85: 376-382.

374. Gao L, Chan SW, Li X, Chen S, Hao Y (2010) Evaluation of an interpersonal-psychotherapy-oriented childbirth education programme for Chinese first-time childbearing women: A randomised controlled trial. International Journal of Nursing Studies 47: 1208-1216 1209p.

375. Chang LW, Kagaayi J, Nakigozi G, Ssempijja V, Packer AH, et al. (2010) Effect of peer health workers on AIDS care in Rakai, Uganda: a cluster-randomized trial. pp. e10923.

376. Grant AD, Coetzee L, Fielding KL, Lewis JJ, Ntshele S, et al. (2010) 'Team up against TB': Promoting involvement in Thibela TB, a trial of community-wide tuberculosis preventive therapy. pp. S37-s44.

377. Tripathy P, Nair N, Barnett S, Mahapatra R, Borghi J, et al. (2010) Effect of a participatory intervention with women's groups on birth outcomes and maternal depression in Jharkhand and Orissa, India: a cluster-randomised controlled trial. pp. 1182-1192.

378. Yeomans PD, Forman EM, Herbert JD, Yuen E (2010) A randomized trial of a reconciliation workshop with and without PTSD psychoeducation in Burundian sample. pp. 305-312.

379. Nct, University of Colorado D, National Institute on Drug A (2010) Peer Leaders as HIV Risk Reduction Change Agents Among Injection Drug Users (IDUs) in Ukraine.

380. Ramesh BM, Beattie TS, Shajy I, Washington R, Jagannathan L, et al. (2010) Changes in risk behaviours and prevalence of sexually transmitted infections following HIV preventive interventions among female sex workers in five districts in Karnataka state, south India. Sexually Transmitted Infections 86 Suppl 1: i17-24.

381. Young S, Konda K, Caceres C, Galea J, Sung-Jae L, et al. (2011) Effect of a Community Popular Opinion Leader HIV/STI Intervention on Stigma in Urban, Coastal Peru. AIDS & Behavior 15: 930-937 938p.

382. Fritz K, McFarland W, Wyrod R, Chasakara C, Makumbe K, et al. (2011) Evaluation of a peer network-based sexual risk reduction intervention for men in beer halls in Zimbabwe: results from a randomized controlled trial. pp. 1732-1744.

383. Hamad R, Fernald L, Karlan DS (2011) Health education for microcredit clients in Peru: a randomized controlled trial. pp. 51.

384. Tylleskär T, Jackson D, Meda N, Engebretsen IM, Chopra M, et al. (2011) Exclusive breastfeeding promotion by peer counsellors in sub-Saharan Africa (PROMISE-EBF): a cluster-randomised trial. pp. 420-427.

385. Yang X, Xia G, Li X, Latkin C, Celentano D (2011) The efficacy of a peer-assisted multi-component behavioral intervention among female entertainment workers in China: an initial assessment. pp. 1509-1518.

386. Alamo S, Wabwire-Mangen F, Kenneth E, Sunday P, Laga M, et al. (2012) Task-Shifting to Community Health Workers: Evaluation of the Performance of a Peer-Led Model in an Antiretroviral Program in Uganda. AIDS Patient Care & Stds 26: 101-107 107p.

387. Bulduk S, Erdogan S (2012) The Effects of Peer Education on Reduction of the HIV/Sexually Transmitted Infection Risk Behaviors Among Turkish University Students. JANAC: Journal of the Association of Nurses in AIDS Care 23: 233-243 211p.

388. Gao L-l, Chan SW-c, Sun K (2012) Effects of an interpersonal-psychotherapy-oriented childbirth education programme for Chinese first-time childbearing women at 3-month follow up: Randomised controlled trial. International Journal of Nursing Studies 49: 274-281 278p.

389. Ibrahim N, Rampal L, Jamil Z, Zain AM (2012) Effectiveness of peer-led education on knowledge, attitude and risk behavior practices related to HIV among students at a Malaysian public university - A randomized controlled trial. Preventive Medicine 55: 505-510 506p.

390. Nor B, Ahlberg BM, Doherty T, Zembe Y, Jackson D, et al. (2012) Mother's perceptions and experiences of infant feeding within a community-based peer counselling intervention in South Africa. Maternal & Child Nutrition 8: 448-458 411p.

391. Van Tam V, Larsson M, Pharris A, Diedrichs B, Nguyen HP, et al. (2012) Peer support and improved quality of life among persons living with HIV on antiretroviral treatment: A randomised controlled trial from north-eastern Vietnam. Health & Quality of Life Outcomes 10: 53-53 51p.

392. García PJ, Holmes KK, Cárcamo CP, Garnett GP, Hughes JP, et al. (2012) Prevention of sexually transmitted infections in urban communities (Peru PREVEN): a multicomponent community-randomised controlled trial. pp. 1120-1128.

393. Go V, Frangakis C, Nguyen Le M, Tran Viet H, Latkin C, et al. (2012) Effects of an HIV/AIDS peer prevention intervention on sexual and injecting risk behaviours among injecting drug users (IDU) and their risk partners in Thai Nguyen, Vietnam: A randomized controlled trial. pp. 151-152.

394. Morris J, Jones L, Berrino A, Jordans MJ, Okema L, et al. (2012) Does combining infant stimulation with emergency feeding improve psychosocial outcomes for displaced mothers and babies? A controlled evaluation from northern Uganda. pp. 349-357.

395. Peltzer K, Simbayi L, Banyini M, Kekana Q (2012) HIV risk reduction intervention among medically circumcised young men in South Africa: a randomized controlled trial. pp. 336-341.

396. Baghianimoghadam MH, Hadavandkhani M, Mohammadi M, Fallahzade H, Baghianimoghadam B (2012) Current education versus peer-education on walking in type 2 diabetic patients based on Health Belief Model: a randomized control trial study. Romanian journal of internal medicine = Revue roumaine de medecine interne 50: 165-172.

397. Moshki M, Amiri M, Khosravan S (2012) Mental health promotion of Iranian university students: The effect of self-esteem and health locus of control. Journal of Psychiatric and Mental Health Nursing 19: 715-721.

398. Saad A, Lekhraj R, Sabitu K, AbdulRahman H, Awaisu A, et al. (2012) An HIV-STI risk reduction program among undergraduate students at a northern Nigerian university: A randomized controlled field trial. Journal of Public Health (Germany) 20: 549-559.

399. Decat P, Zhang W-H, Delva W, Moyer E, Cheng Y, et al. (2012) Promoting contraceptive use among female rural-to-urban migrants in Qingdao, China: A comparative impact study of worksite-based interventions. European Journal of Contraception and Reproductive Health Care 17: 363-372.

400. Lewycka S, Mwansambo C, Rosato M, Kazembe P, Phiri T, et al. (2013) Effect of women's groups and volunteer peer counselling on rates of mortality, morbidity, and health behaviours in mothers and children in rural Malawi (MaiMwana): a factorial, cluster-randomised controlled trial. Lancet 381 North American Edition: 1721-1735 1715p.

401. Ochola SA, Labadarios D, Nduati RW (2013) Impact of counselling on exclusive breast-feeding practices in a poor urban setting in Kenya: a randomized controlled trial. Public Health Nutrition 16: 1732-1740 1739p.

402. Doherty T, Tabana H, Jackson D, Naik R, Zembe W, et al. (2013) Effect of home based HIV counselling and testing intervention in rural South Africa: cluster randomised trial. pp. f3481.

403. Duan Y, Zhang H, Wang J, Wei S, Yu F, et al. (2013) Community-based peer intervention to reduce HIV risk among men who have sex with men in Sichuan province, China. pp. 38-48.

404. Go VF, Frangakis C, Minh N, Latkin CA, Ha TV, et al. (2013) Effects of an HIV peer prevention intervention on sexual and injecting risk behaviors among injecting drug users and their risk partners in Thai Nguyen, Vietnam: a randomized controlled trial. pp. 154-164.

405. Graff Zivin J, Orozco V, Thirumurthy H, Sakho C, Diallo P, et al. (2013) Social mobilization and peer-mentoring to encourage voluntary counseling and testing and post-testing behavior: A randomized experiment in senegal.

406. Heeren GA, Jemmott JB, Ngwane Z, Mandeya A, Tyler JC (2013) A randomized controlled pilot study of an HIV risk-reduction intervention for sub-Saharan African university students. pp. 1105-1115.

407. Kiweewa FM, Wabwire D, Nakibuuka J, Mubiru M, Bagenda D, et al. (2013) Noninferiority of a task-shifting HIV care and treatment model using peer counselors and nurses among Ugandan women initiated on ART: evidence from a randomized trial. pp. e125-132.

408. Li L, Guan J, Liang LJ, Lin C, Wu Z (2013) Popular Opinion Leader intervention for HIV stigma reduction in health care settings. pp. 327-335.

409. Ross R, Sawatphanit W, Suwansujarid T, Stidham AW, Drew BL, et al. (2013) The effect of telephone support on depressive symptoms among HIV-infected pregnant women in Thailand: an embedded mixed methods study. pp. e13-24.

410. Wingood GM, Reddy P, Lang DL, Saleh-Onoya D, Braxton N, et al. (2013) Efficacy of SISTA South Africa on sexual behavior and relationship control among isiXhosa women in South Africa: results of a randomized-controlled trial. pp. S59-65.

411. Jayakrishnan R, Mathew A, Uutela A, Auvinen A, Sebastian P (2013) Multiple Approaches and Participation Rate for a Community Based Smoking Cessation Intervention Trial in Rural Kerala, India. Asian Pacific Journal of Cancer Prevention 14: 2891-2896.

412. Naidoo S, Morar NS, Ramjee G (2013) Participants as community-based peer educators: Impact on a clinical trial site in KwaZulu-Natal. South African Journal of Science 109: 01-05.

413. Beattie TSH, Mohan HL, Bhattacharjee P, Chandrashekar S, Isac S, et al. (2014) Community Mobilization and Empowerment of Female Sex Workers in Karnataka State, South India: Associations With HIV and Sexually Transmitted Infection Risk. American Journal of Public Health 104: 1516-1525 1510p.

414. Richter L, Rotheram-Borus M, Heerden A, Stein A, Tomlinson M, et al. (2014) Pregnant Women Living with HIV (WLH) Supported at Clinics by Peer WLH: A Cluster Randomized Controlled Trial. AIDS & Behavior 18: 706-715 710p.

415. Blebil AQ, Sulaiman SA, Hassali MA, Dujaili JA, Zin AM (2014) Impact of additional counselling sessions through phone calls on smoking cessation outcomes among smokers in Penang State, Malaysia. pp. 460.

416. Chang LW, Nakigozi G, Billioux V, Serwadda D, Quinn T, et al. (2014) Peer support and engagement, HIV care and sexual behaviors among PLHIV not on art: a randomized trial. pp. 561-562.

417. Clarke K, Azad K, Kuddus A, Shaha S, Nahar T, et al. (2014) Impact of a participatory intervention with women's groups on psychological distress among mothers in rural Bangladesh: Secondary analysis of a cluster-randomised controlled trial.

418. Maman S, Moodley D, McNaughton-Reyes HL, Groves AK, Kagee A, et al. (2014) Efficacy of enhanced HIV counseling for risk reduction during pregnancy and in the postpartum period: a randomized controlled trial. pp. e97092.

419. Petersen I, Hanass Hancock J, Bhana A, Govender K (2014) A group-based counselling intervention for depression comorbid with HIV/AIDS using a task shifting approach in South Africa: A randomized controlled pilot study. pp. 78-84.

420. Rotheram-Borus MJ, Richter LM, Heerden A, Rooyen H, Tomlinson M, et al. (2014) A cluster randomized controlled trial evaluating the efficacy of peer mentors to support South African women living with HIV and their infants.

421. Wouters E, Masquillier C, Ponnet K, Roux Booysen F (2014) A peer adherence support intervention to improve the antiretroviral treatment outcomes of HIV patients in South Africa: the moderating role of family dynamics. pp. 145-153.

422. Yao L, Ju C, Ye X, Sun Z, Xie B, et al. (2014) Effect of peer support intervention on diabetes distress: A pilot for a randomized controlled trial. pp. 57-58.

423. Rashid RA, Kamali K, Habil MH, Shaharom MH, Seghatoleslam T, et al. (2014) A mosque-based methadone maintenance treatment strategy: implementation and pilot results. International Journal of Drug Policy 25: 1071-1075.

424. White JS (2014) A team-based behavioral economics experiment on smoking cessation. Dissertation Abstracts International: Section B: The Sciences and Engineering 75: No Pagination Specified.

425. Bolton P, Lee C, Haroz EE, Murray L, Dorsey S, et al. (2014) A Transdiagnostic Community-Based Mental Health Treatment for Comorbid Disorders: Development and Outcomes of a Randomized Controlled Trial among Burmese Refugees in Thailand. PLoS Medicine 11.

426. Wagner N, Arcand J-L, Sakho C, Diallo PA (2014) HIV/AIDS sensitisation and peer mentoring: evidence from a randomised experiment in Senegal. Journal of Development Effectiveness 6: 147-166.

427. Maarefvand M, Eghlima M, Rafiey H, Rahgozar M, Tadayyon N, et al. (2015) Community-Based Relapse Prevention for Opiate Dependents: A Randomized Community Controlled Trial. Community mental health journal 51: 21-29 29p.

428. Masquillier C, Wouters E, Mortelmans D, Roux Booysen F (2015) The Impact of Community Support Initiatives on the Stigma Experienced by People Living with HIV/AIDS in South Africa. AIDS & Behavior 19: 214-226 213p.

429. Coker M, Etiebet MA, Chang H, Awwal G, Jumare J, et al. (2015) Socio-demographic and adherence factors associated with viral load suppression in hiv-infected adults initiating therapy in northern nigeria: A randomized controlled trial of a peer support intervention. pp. 279-285.

430. Lubega M, Tumwesigye NM, Kadobera D, Marrone G, Wabwire-Mangen F, et al. (2015) Effect of community support agents on retention of people living with HIV in pre-antiretroviral care: A randomized controlled trial in eastern Uganda. pp. e36-e43.

431. Simmons N, Donnell D, Ou SS, Celentano DD, Aramrattana A, et al. (2015) Assessment of contamination and misclassification biases in a randomized controlled trial of a social network peer education intervention to reduce HIV risk behaviors among drug users and risk partners in Philadelphia, PA and Chiang Mai, Thailand. pp. 1818-1827.

432. Singla DR, Kumbakumba E, Aboud FE (2015) Effects of a parenting intervention to address maternal psychological wellbeing and child development and growth in rural Uganda: A community-based, cluster-randomised trial.

433. Wagman JA, Gray RH, Campbell JC, Thoma M, Ndyanabo A, et al. (2015) Effectiveness of an integrated intimate partner violence and HIV prevention intervention in Rakai, Uganda: analysis of an intervention in an existing cluster randomised cohort. pp. e23-33.

434. Young SD, Cumberland WG, Nianogo R, Menacho LA, Galea JT, et al. (2015) The HOPE social media intervention for global HIV prevention in Peru: A cluster randomised controlled trial. pp. e27-e32.

435. Traore IT, Meda N, Hema NM, Ouedraogo D, Some F, et al. (2015) HIV prevention and care services for female sex workers: Efficacy of a targeted community-based intervention in Burkina Faso. Journal of the International AIDS Society 18 (1) (no pagination).

436. Domingues MR, Bassani DG, da Silva SG, Coll Cde V, da Silva BG, et al. (2015) Physical activity during pregnancy and maternal-child health (PAMELA): study protocol for a randomized controlled trial. Trials [Electronic Resource] 16: 227.

437. Puchalski Ritchie LM, Schull MJ, Martiniuk AL, Barnsley J, Arenovich T, et al. (2015) A knowledge translation intervention to improve tuberculosis care and outcomes in Malawi: a pragmatic cluster randomized controlled trial. Implementation Science 10: 38.

438. Chang LW, Nakigozi G, Billioux VG, Gray RH, Serwadda D, et al. (2015) Effectiveness of peer support on care engagement and preventive care intervention utilization among pre-antiretroviral therapy, HIV-infected adults in Rakai, Uganda: A randomized trial. AIDS and Behavior 19: 1742-1751.

439. Deng K, Ren Y, Luo Z, Du K, Zhang X, et al. (2016) Peer support training improved the glycemic control, insulin management, and diabetic behaviors of patients with type 2 diabetes in rural communities of Central China: A randomized controlled trial. pp. 267-275.

440. Tripathy P, Nair N, Sinha R, Gope RK, Rath S, et al. (2016) Effect of participatory women's groups facilitated by Accredited Social Health Activists on birth outcomes in rural eastern India: A cluster-randomised controlled trial. pp. e119-e128.

441. Irct201601231407N, Vice chancellor for R, Technology of Qazvin University of Medical Sciences. Qazvin I (2016) Assessing the effect of face to face education and training with short messages on the body mass index and nutritional habits of students.

442. Arem H, Nakyanjo N, Kagaayi J, Mulamba J, Nakigozi G, et al. (2011) Peer Health Workers and AIDS Care in Rakai, Uganda: A Mixed Methods Operations Research Evaluation of a Cluster-Randomized Trial. AIDS Patient Care & Stds 25: 719-724 716p.

443. Morisky DE, Malow RM, Tiglao TV, Lyu SY, Vissman AT, et al. (2010) Reducing sexual risk among Filipina female bar workers: effects of a CBPR-developed structural and network intervention. pp. 371-385.

444. Li C, Chu F, Wang H, Wang XP (2014) Efficacy of Williams LifeSkills training for improving psychological health: a pilot comparison study of Chinese medical students. pp. 161-169.

445. Hidiroglu S, Topuzoglu A, Onsuz MF (2017) A community-based intervention programme on hormonal contraceptives: the utilisation of social networks. Journal of obstetrics and gynaecology. pp. 126-130.

446. Wang D, Stewart D, Chang C (2016) School-based intervention for nutrition promotion in Mi Yun County, Beijing, China Does a health-promoting school approach improve parents' knowledge, attitudes and behaviour? Health Education 116: 310-319.

447. Isrctn, Cardiff U (2000) Randomised controlled trial of the effectiveness of a schools-based, peer-led, smoking intervention <Acronym />.

448. Isrctn, University College L (2000) A Randomised Intervention trial of PuPil-Led sex Education in schools <Acronym />.

449. Nct, San Diego State U, National Heart L, Blood I (2000) CVD Nutrition Education for Low Literacy ESL Students <Acronym />.

450. Nct, National Heart L, Blood I (2001) Peer-Based Skills Training to Enhance Teen Weight Loss <Acronym />.

451. Nct, National Institute of A, Infectious D, Eunice Kennedy Shriver National Institute of Child H, Human D, et al. (2002) A Study of Peer Education to Prevent HIV Transmission Among Injection Drug Users and Their HIV Risk Contacts.

452. Nct, Eunice Kennedy Shriver National Institute of Child H, Human D (2003) Peer Mentors for Adolescents in HIV Affected Families.

453. Isrctn, Nhs R, amp, Health DRPR-Do (2004) Randomised controlled study of group therapy for adolescents who self-harm <Acronym />.

454. Isrctn, Nhs R, amp, Health DRPR-Do (2004) Randomised controlled trial of the effects of training peer reviewers on the quality of review <Acronym />.

455. Nct, National Institute of A, Infectious D, Eunice Kennedy Shriver National Institute of Child H, Human D (2004) Directly Observed Therapy in HIV Infected Adolescent Focus Groups.

456. Isrctn, Centre for A, Mental H (2005) Controlled longitudinal evaluation of a school based program to prevent adolescent dating violence and related risk behaviours.

457. Nct, Eunice Kennedy Shriver National Institute of Child H, Human D, National Institute on Drug A, National Institute of Mental H, et al. (2005) Connect to Protect Partnerships for Youth Prevention Interventions: Phase II.

458. Nct, The Center for Mind-Body M (2005) Treatment of Posttraumatic Stress Disorder in Kosovar High School Students Using Mind-Body Skills Groups.

459. Nct, Centers for Disease C, Prevention, Health Research A (2005) Multi-level Interventions for STD Prevention Among Adolescents.

460. Nct, University of P, Eunice Kennedy Shriver National Institute of Child H, Human D (2005) The S.A.F.E. Study : Computer-Aided Counseling to Prevent Teen Pregnancy/Sexually Transmitted Diseases (STDs).

461. Nct, Centers for Disease C, Prevention (2005) Adolescent Impact: A Behavioral Intervention for Adolescents Living With HIV/AIDS.

462. Nct, Centers for Disease C, Prevention (2005) Community Intervention Trial for Youth (CITY) Study.

463. Nct, Westat, National Institute on Drug A, National Institute of Mental H, National Institute on Alcohol A, et al. (2005) Development of a Life Skills Intervention for Young Adolescents Perinatally-Infected With HIV and Their Caregivers.

464. Nct, University of California SF, Associates ETR, William, Flora Hewlett F (2005) The Young Woman's Reach Project: Trial of an Intervention to Impact Contraceptive Behavior, Unintended Pregnancy, and Sexually Transmitted Infections (STIs) Among Adolescent Females.

465. Nct, Westat, National Institute on Drug A, National Institute of Mental H, National Institute on Alcohol A, et al. (2005) Intervention Development for Newly Diagnosed Youth With HIV.

466. Nct, Rutgers U, National Institute of Mental H (2005) Comparison of Three Therapy-Based Interventions for Preventing Depression in Adolescents.

467. Nct, New York State Psychiatric I, National Institute of Mental H (2005) Group Interpersonal Psychotherapy for Depressed Adolescents in School-based Clinics.

468. Nct, Westat, National Institute on Drug A, National Institute of Mental H, National Institute on Alcohol A, et al. (2005) C2P (With Venues): Connect to Protect® Partnerships For Youth Prevention Interventions.

469. Nct, Yale U, National Institute of Mental H (2005) Group Prenatal Care for Reducing the Risk of STDs in Pregnant Young Women.

470. Actrn, Hospital The Children's Hospital at W, Nil (2006) Community-based weight management of overweight and obese adolescents: a randomised controlled trial.

471. Isrctn, The BF (2006) Telecare motivational interviewing for diabetes patient education and support: a randomised controlled trial based in primary care comparing nurse and peer supporter delivery <Acronym />.

472. Nct, National Institute of D, Digestive, Kidney D (2006) Peer-enhanced Intervention to Support Teen Weight Loss.

473. Nct, Canadian Research Institute for Social P, Women's Health Research U, Social Support Research P, University Health Network T, et al. (2006) Trial to Evaluate the Effect of Home-Based Peer Support on Maternal-Infant Interaction, Infant Health Outcomes, and Postpartum Depression (PPD).

474. Nct, National Institute of Environmental Health S (2006) A Cohort Study of Smoking Prevention and Health Promotion for Middle School Students in Wuhan, China.

475. Nct, King's College L, Action on A (2006) Personality-targeted Interventions for Adolescent Alcohol Misuse.

476. Nct, Anhui Medical U (2006) Evaluation of Interventions to Prevent Suicide of Medical Students Through the School-Based Social Support System.

477. Isrctn, University of B (2007) A single blind randomised controlled trial to determine the effectiveness of group Cognitive Behaviour Therapy (CBT) in the prevention of depression in high risk adolescents <Acronym />.

478. Isrctn, Accare (2007) Evaluation of a group training for adolescents (Emotion Regulation Training) with emotion regulation problems - a randomised controlled clinical trial <Acronym />.

479. Nct, University of WH (2007) Obesity Intervention "Obeldicks" for Obese Children, Adolescents and Their Parents.

480. Nct, University of Massachusetts W (2007) Provider and Peer Delivered Youth Smoking Intervention.

481. Nct, Medical University of V, National Bank of A (2007) Randomised Controlled Study on the Use of Psychoeducation for Bulimia Nervosa in Young Women.

482. Nct, Westat, National Institute on Drug A, National Institute of Mental H (2007) Intervention for Newly Diagnosed Youth With HIV.

483. Nct, McGill University Health C (2007) Mentors in Motion: A Physical Activity Intervention for Obese Adolescents.

484. Actrn, Individual Caroline H, Kathryn B (2008) Evaluation of a group program for anxious adolescents who have been bullied at school.

485. Actrn, University Deakin U, Tonga Ministry of H, Fiji School of M (2008) Ma'alahi Youth Project; The effects of a community based intervention promoting healthy eating and physical activity in secondary school students on changes in body size and composition.

486. Actrn, University The University of M (2008) Health and coping in type 1 diabetes: The effects of a psycho-educational program to improve metabolic control in adolescents with type 1 diabetes.

487. Isrctn, The Ipswich Hospital NHST (2008) A randomised controlled trial to determine whether family centred structured education improves blood glucose control in adolescents with type 1 diabetes <Acronym />.

488. Nct, Children's Hospital B (2008) Medical Office Intervention for Adolescent Drug Use - Attention Study Supplement.

489. Nct, Children's Hospital B (2008) Mentored Clinical Career Award in Adolescent Substance Abuse.

490. Nct, Children's H, Health System Foundation W (2008) Intervention Groups for Adolescents With Type 1 Diabetes Mellitus.

491. Nct, University of M, National Institute of Mental H (2008) A Waitlist-Controlled Trial of the Unified Protocol for the Treatment of Emotional Disorders in Adolescents.

492. Nct, University of California LA, National Institute of Mental H (2008) Effectiveness of a Family-Based Intervention for Adolescent Suicide Attempters (The SAFETY Study).

493. Nct, Uni R, Sykehuset i Vestfold HF, Helse S-O (2008) Ibedrift- A Trial Comparing a New Approach to Musculoskeletal Pain Consisting of Education and Peer Involvement to Treatment as Usual.

494. Nct, University of M, Annie ECF, The Thomas Wilson Sanitarium for Children of Baltimore C, Johns Hopkins Bloomberg School of Public H, et al. (2008) Challenge!, a Health Promotion/ Obesity Prevention Program for Teens.

495. Nct, University of Colorado D, The Kempe Foundation for the P, Treatment of Child A, Neglect, et al. (2008) Intervention Development and Pilot for Foster Care Youth.

496. Actrn, University University of A (2009) Randomised controlled trial of exercise duration and mood in adolescents.

497. Actrn, Other Burnet I, Monash U (2009) Randomised controlled trial of paper, online and text message diaries for sexual health data collection from young people.

498. Actrn, Curtin University of T (2009) Amoung obese adolescents, does a multidisciplinary intervention (Curtin Activity, Food and Attitudes program) lead to improved mental health status?

499. Actrn, Individual Professor Brian O (2009) A controlled evaluation of the Australasian Peers for Progress Diabetes Program (PfP-DP) and its transferability to other countries.

500. Actrn, Individual Sarah S (2009) Resistance weight training to improve body composition and biochemical markers in obese adolescents with varying metabolic profiles: a randomised controlled trial.

501. Isrctn, Cancer Research UK (2009) Evaluation of QUIT's Break Free smoking cessation intervention for young people.

502. Isrctn, University of W (2009) Mums 4 Mums: telephone peer support for women experiencing post-natal depression.

503. Nct, Women, Infants Hospital of Rhode I, University of Rhode I (2009) Partner-Specific HIV Risk Reduction Intervention for Drug-Using Adolescents.

504. Nct, University of California LA, National Institutes of H (2009) An Arts Intervention for Drug-Using Homeless Youth.

505. Nct, Eunice Kennedy Shriver National Institute of Child H, Human D, National Institute on Drug A, National Institute of Mental H (2009) Empowerment Intervention for Young Women - Phase I.

506. Nct, University of K (2009) Trauma and Truth Interventions (NET) Versus Conflict Resolution and Social Skills Trainings for Vulnerable Youths in Northern Uganda.

507. Nct, The Hospital for Sick C, The Provincial Centre of Excellence for C, Youth Mental Health at C (2009) Effects Of A Computerized Working Memory Training Program On Attention, Working Memory, And Academics, In Adolescents With Severe ADHD/LD.

508. Nct, Lawson Health Research I, Canadian Institutes of Health R (2009) Activity and Metformin Intervention in Obese Adolescents.

509. Nct, Children's Hospital Medical Center C, National Institute of Mental H (2009) Comparing School Based Interventions for Adolescents With Attention Deficit Hyperactivity Disorder.

510. Nct, University of Colorado D, National Institute on Drug A (2009) Martial Arts as Early Intervention for Teen Drug Abuse.

511. Nct, University of Sao Paulo General H (2009) Physical Activity and Nutritional Education as School-based Interventions to Control Obesity in Children and Adolescents.

512. Nct, University G, Institute of Tropical Medicine B, Nutrition Third World B, Vlir-Iuc B (2009) Health Promotion in Adolescents in Ecuador.

513. Nct, Brooke Army Medical C, Wilford Hall Medical C (2009) "Efficacy in Adolescents of Continued Behavior Modification Following a Six Month Sibutramine-based Weight Management Intervention".

514. Wilson AJ, Prapavessis H, Jung ME, Cramp AG, Vascotto J, et al. (2009) Lifestyle modification and metformin as long-term treatment options for obese adolescents: study protocol. BMC public health 9.

515. Li Y, Hu X, Zhang Q, Liu A, Fang H, et al. (2010) The nutrition-based comprehensive intervention study on childhood obesity in China (NISCOC): a randomised cluster controlled trial. pp. 229.

516. Actrn, University The Australian National U (2010) Randomised controlled trial of two implementation methods of the e-couch Anxiety and Worry program in an adolescent school-based population.

517. Isrctn, University of B (2010) Randomised controlled trial of a school-based intervention to improve the mental health of low-income, secondary school students in Santiago, Chile.

518. Isrctn, Universiti Putra M (2010) Integrated human immunodeficiency virus (HIV)-sexually transmitted infections (STI) risk reduction intervention program among youths.

519. Nct, University of C, National Institute on Alcohol A, Alcoholism (2010) Motivational Interventions for Lifestyle and Exercise in College Students.

520. Nct, Duke U, National Heart L, Blood I, National Heart L, et al. (2010) Cell Phone Intervention in Young Adults.

521. Nct, Hunter C (2010) Risk Reduction Intervention for Vulnerable Young Adult Males.

522. Nct, Massachusetts General H (2010) Computerized Information-Processing Bias Retraining in Depressed Adolescents.

523. Nct, Mayo C, James, Esther King Biomedical Research P (2010) Integrated Behavioral Intervention for Cigarette Smoking and Binge Drinking in Young Adults.

524. Nct, Institute of Child H (2010) Evaluating PEGASUS - a Group Intervention for Young People With an Autism Spectrum Disorder.

525. Nct, Centre Hospitalier Universitaire de B (2010) Effects of Physical Training and Nutrition Education on Adipocytokines in Severely Obese Teenagers.

526. Nct, Assaf-Harofeh Medical C (2010) Psychosocial Interventions in the Treatment of Youth With Type 1 Diabetes Mellitus.

527. Nct, Rutgers U, National Institute of Mental H, National Institute of Mental H (2010) Depression Prevention Initiative - A Study of Interpersonal Psychotherapy-Adolescent Skills Training (IPT-AST) in School Settings.

528. Nct, Boston Medical C (2010) An Intervention Study To Improve HPV Immunization in Haitian and African American Girls.

529. Nct, Westat, National Institute on Drug A, National Institute of Mental H (2010) Development of an Empowerment Intervention for Young Women Living With HIV.

530. Eble A, Mann V, Bhakta P, Lakshminarayana R, Frost C, et al. (2010) The STRIPES Trial - Support to Rural India's Public Education System. Trials 11.

531. Rotheram-Borus MJ, Richter L, Rooyen H, Heerden A, Tomlinson M, et al. (2011) Project Masihambisane: a cluster randomised controlled trial with peer mentors to improve outcomes for pregnant mothers living with HIV. pp. 2.

532. Actrn, Government body SAH, University of South A (2011) A randomised controlled trial assessing the effects of a school-based sleep intervention in Year 6 and 7 students.

533. Actrn, Griffith U, University of Q (2011) Factors predicting therapy outcome in online cognitive behaviour therapy (with minimal therapist assistance) in the treatment of anxiety disorders among children and adolescents.

534. Irct201012235440N, Research Institute for Endocrine S (2011) Effectiveness of an educational program to adolescents' weight management.

535. Isrctn, Newcastle U (2011) A brief alcohol intervention to prevent hazardous drinking in young people aged 14-15 in a high school setting.

536. Isrctn, University of the A (2011) Targeted school based intervention to improve depressive symptoms among at risk Chilean adolescents.

537. Isrctn, University of S (2011) Investigating the effectiveness of a working memory training intervention to increase educational achievement and reduce anxiety in young people.

538. Isrctn, German Federal Ministry of H (2011) CAN Stop - psychoeducation and relapse prevention for young persons with problematic cannabis use.

539. Jprn U, Department of Preventive Services KuSoPh (2011) A Randomized Controlled Trial to Assess the Effectiveness of Peer-assisted Learning on Body Weight Reduction in Health Guidance <Acronym />.

540. Nct, University of North Carolina CH, National Institute of D, Digestive, Kidney D, et al. (2011) FL3X Study: An Adaptive Intervention to Improve Outcomes for Youth With Type 1 Diabetes (FL3X).

541. Nct, Karolinska I, Stockholm County Council S, City of Stockholm Competence F, Lindeparkens upper secondary school for intellectual disability s, et al. (2011) School Intervention With Daily Physical Activity and Healthy Food for Students With an Intellectual Disability.

542. Nct, University of Illinois at C, Substance A, Mental Health Services A (2011) A Randomized Controlled Trial of Mental Health Peer-Led Education.

543. Nct, Seattle Children's H (2011) Internet Intervention for Adolescents With Chronic Pain.

544. Nct, Rhode Island H (2011) Dating Violence and HIV Prevention in Girls: Adapting Mental Health Interventions.

545. Nct, University of M, National Institute on Drug A (2011) Project Chill: Tailored Youth Drug Intervention In Primary Care.

546. Nct, Michigan State U (2011) Middle School Physical Activity Intervention for Girls.

547. Nct, Ann, amp, Robert HLCsHoC, National Institute on Drug A (2011) Text Messaging Intervention to Improve ART Adherence Among HIV-positive Youth.

548. Nct, Johns Hopkins U, Rakai Health Sciences P (2011) The PeerCARE Study (Peer Community-based Assistant in REtention).

549. Nct, Children's Hospital B (2011) FITT Exercise Counseling With Interactive Accelerometry and Physical Activity in Adolescents at Increased Risk of Early Cardiovascular Disease: A Pilot Study.

550. Nct, University Hospital C-F (2011) CANABIC : CANnabis and Adolescents, a Brief Intervention (BI) to Reduce Their Consumption.

551. Nct, Weill Medical College of Cornell U (2011) Stress Management Intervention in Inner City Adolescents With Asthma.

552. Nct, Massachusetts General H (2011) Development and Testing of Adolescent Twelve-Step Facilitation.

553. Nct, University of Massachusetts W (2011) School Nurse Intervention and After School Exercise Program for Overweight Teens.

554. Nct, Sante Diabete M, Bridges p (2011) A Randomized Trial of an Intensive Education Intervention Using a Network of Involved Diabetic Patients (Peer Educators) to Improve Glycemic Control of Type 2 Diabetic Patients.

555. Nct, Ann, amp, Robert HLCsHoC (2011) Resilience Promotion in Teens With Type 1 Diabetes: Preventing Negative Outcomes.

556. Nct, Queen's University B (2011) Randomized Control Trial of Group Intervention With Former War-affected Boys in the Democratic Republic of Congo.

557. Nct, Pacific Institute for R, Evaluation, National Institute of Mental H, Moi U, et al. (2011) School Support as Structural HIV Prevention for Adolescent Orphans in Kenya.

558. Nct, Michigan State U, University of M (2011) Girls on the Move Intervention to Increase Physical Activity Among Middle School Girls.

559. Ntr, Brabant GGZO (2011) A Randomized Controlled Trial Testing the Effectiveness of an Indicated Depression Prevention Program ('Op Volle Kracht') Among Adolescent Girls with Elevated Depressive Symptoms.

560. Wijesuriya M, Gulliford M, Vasantharajah L, Viberti G, Gnudi L, et al. (2011) DIABRISK - SL Prevention of cardio-metabolic disease with life style modification in young urban Sri Lankan's - study protocol for a randomized controlled trial. Trials 12.

561. Betancourt T (2012) A Feasibility Trial of the Youth Readiness Intervention: A Group Psychosocial Intervention for War-affected Youth in Sierra Leone.

562. Overgaard HJ, Alexander N, Matiz MI, Jaramillo JF, Olano VA, et al. (2012) Diarrhea and dengue control in rural primary schools in Colombia: Study protocol for a randomized controlled trial.

563. Xu F, Ware RS, Tse LA, Wang Z, Hong X, et al. (2012) A school-based comprehensive lifestyle intervention among chinese kids against obesity (CLICK-Obesity): rationale, design and methodology of a randomized controlled trial in Nanjing city, China. pp. 316.

564. Actrn, Other The George Institute for Global Health C, The University of S (2012) Beijing Adolescent Obesity Peer Education.

565. Actrn, Individual Professor Martin E, Professor Knut W (2012) Utilizing the Internet to deliver mental health self-help interventions to senior high-school students.

566. Chi CO, The George Institute for Global Health C (2012) A pilot study of school-based peer education and obesity-related behaviours in adolescents in Beijing, China.

567. Ctri, St Johns Research I (2012) A study to test the effectiveness of a school-based educational program on risk factors related to heart diseases among high school students in selected rural schools.

568. Ctri, Indian Council Of Medical R (2012) Impact of Health Promoting lifestyle intervention on Manipuri and Non-Manipuri University Graduate Students in a North Indian City.

569. Drks, Universitätsklinik für Psychiatrie u. Psychosomatik AfPuPiK-uJ (2012) Evaluation of the Freiburg Social Skills Training Program with Focus on Theory of Mind for Children and Adolescents with High-Functioning Autism Spectrum Disorders (TOMTASS).

570. Drks, Integriertes Forschungs- und Behandlungszentrum AdipositasErkrankungen UL (2012) Feasibility and efficacy of a weight maintenance treatment approach for adolescent obesity via telephone counseling following an obesity treatment program: a randomized controlled trial.

571. Irct201110153027N, Research Deputy of Tabriz University of Medical S (2012) Effects of peer education on knowledge and practice of ninth – grade girl students about iron deficiency and use of iron supplements.

572. Irct201202087352N, Tehran University Of Medical S (2012) Peace Education and Empowering Primary School Students.

573. Irct201202266582N, Tabriz university of medical sciences-Vice chancellor for r (2012) Effect of peer support on anxiety, self-esteem and postpartum depression.

574. Isrctn, Cambridge University Hospitals NHSFT (2012) RAPSID: Can peer supprt, delivered as a group or individual intervention, enable people with diabetes and improve their health? A RAndomised controlled trial of Peer Support In type 2 Diabetes <Acronym />.

575. Isrctn, University of O (2012) A sport-for-development intervention for the physical and mental health of young adolescents in Gulu, Uganda.

576. Nct, Medical University of V, National Bank of A (2012) Interventions to Prevent Adolescents With Type 1 Diabetes From Long-term Complications.

577. Nct, Yale U, National Institute of Mental H (2012) Effectiveness and Cost Effectiveness of Peer Mentors in Reducing Hospital Use.

578. Nct, University of C, Juvenile Diabetes Research F, Diabetes UK, British Heart F, et al. (2012) Adolescent Type 1 Diabetes Cardio-Renal Intervention Trial.

579. Nct, The Center for Mind-Body M, The Atlantic P (2012) Mind-Body Skills Groups for the Treatment of War-Related Trauma in Adolescents in Gaza.

580. Nct, University of North Carolina CH (2012) A School Health Center Intervention to Increase Adolescent Vaccination.

581. Nct, Fundació d'investigació Sanitària de les Illes B, Instituto de Salud C, III (2012) Effectiveness of Intervention on Reducing the Prevalence of Smoking Among Adolescents.

582. Nct, University of M, National Heart L, Blood I (2012) Tailored Web and Peer Email Cessation Counseling for College Smokers.

583. Nct, Centre for A, Mental H, Canadian Institutes of Health R, Children's Hospital of Eastern O, et al. (2012) Evaluating the Effectiveness of Motivational Interviewing and a Dialectical Therapy Skills- Based Intervention for Youth.

584. Nct, University of California SD, San Diego State U (2012) PACE+: Counseling Adolescents for Exercise and Nutrition.

585. Nct, Harvard School of Public H, Caritas F, International Rescue C, Harvard Medical S, et al. (2012) Trial of the Youth Readiness Intervention.

586. Nct, Oslo, Akershus University College of Applied S, Norwegian School of Sport S (2012) Young, Fit and Happy. A Web-based Intervention to Prevent Obesity in Adolescents.

587. Nct, University of California LA (2012) HOPE (Harnessing Online Peer Education): Using Online Social Networks for HIV Prevention and Testing.

588. Nct, Oregon Social Learning C (2012) Students, Parents, and Teachers On Track: Intervention Development for Youth With Emotional Disturbance.

589. Nct, University of R (2012) HEALTHY (Health Education Adolescent Leadership Training to Help Youth) Leaders.

590. Nct, Azienda Sanitaria Locale CNA-B, University of Eastern P, Eclectica Sas di Beccaria Franca EAeC (2012) A School-based Prevention Intervention to Promote Wellbeing in Preadolescents.

591. Nct, University of C, Heart, Stroke Foundation of C, Canadian Council of Cardiovascular N (2012) Effect of Peer Support Intervention on Early Recovery Outcomes Post Coronary Artery Bypass Graft Surgery.

592. Nct, Seattle Children's H (2012) Peer Counseling in Family-Based Treatment for Childhood Obesity.

593. Pactr, Oxford U (2012) Sanitary Pads for Girls' Education in Africa.

594. Tctr, Preventive m, social department Faculty of medicine C, Preventive m, social department Faculty of medicine C (2012) Effect of Self Management with Peer-support on Improving Health Outcomes of Patients with Type 2 Diabetes in Bangkok Metropolis.

595. Sathish T, Williams ED, Pasricha N, Absetz P, Lorgelly P, et al. (2013) Cluster randomised controlled trial of a peer-led lifestyle intervention program: study protocol for the Kerala diabetes prevention program. BMC public health 13: 1035-1035 1031p.

596. He FJ, Wu Y, Ma J, Feng X, Wang H, et al. (2013) A School-based Education Programme to Reduce salt intake in children and their families (School-EduSalt): Protocol of a cluster randomised controlled trial. BMJ Open 3 (7) (no pagination).

597. Actrn, University University of NSW (2013) The impact of a multi-component intervention (including strategies for engagement, education and skill development, personal development and empowerment, and case management support) on high-risk Indigenous and non-Indigenous young people.

598. Chi CO, Institute of C, Adolescent Health PU (2013) Study on obesity intervention with physical exercise among students in Changping District, Beijing.

599. Irct201201055499N, The Office of the Vice chancellor for research GUoMS (2013) The effectiveness of self-efficacy-based intervention on self-care level in diabetic adolescents.

600. Irct2013050613246N, Vice-chancellor for research TUoMS (2013) Health promotion Girles students from Tehran University of Medical Sciences with anemia through education and iron supplementation.

601. Isrctn, University of D (2013) Internet Delivered Treatment for generalized Anxiety symptoms in students.

602. Nct, Johns Hopkins U (2013) A Multi-Center Randomized Controlled Trial of Mentoring to Prevent Youth Violence.

603. Nct, Yale U, American Diabetes A (2013) Teens-Connect: Preventive Psycho-education for Transitioning Teens With Diabetes.

604. Nct, St. Justine's H (2013) Mindfulness-based Intervention for Teenagers With Cancer.

605. Nct, XXIII AOOPG, University of P (2013) ETOS: Minimal Hepatic Encephalopathy in Childhood and Young Adult: epidemiOlogical Study and Pilot Interventional Study.

606. Nct, Northwestern U, National Institute on Drug A, Emory U, Hunter C (2013) Keep It Up! 2.0: A Comparison of Two Online HIV Intervention Programs for Young Men Who Have Sex With Men.

607. Nct, Milton SHMC, National Institutes of H (2013) Impact of Volunteer Peer-Led Intervention for Weight Control in Primary Care.

608. Nct, Karolinska I, Child, Adolescent Psychiatry S, Stockholm County Council S, et al. (2013) Social Skills Group Training ("KONTAKT") for Children and Adolescent With High-functioning Autism Spectrum Disorders.

609. Nct, Hadassah Medical O (2013) The Efficacy of Group Therapy in Ultra-Orthodox Jewish Young Men Diagnosed With Type One Diabetes <Acronym />.

610. Nct, Northwestern U (2013) Technology Assisted Programs That Promote Mental Health for Teenagers.

611. Nct, Children's Hospital B (2013) A Randomized Controlled Trial of Students for Nutrition and eXercise.

612. Nct, Fhi, Gold Star K (2013) Evaluation of a Peer Education Program to Promote Family Planning Among Female Sex Workers in Kenya.

613. Nct, Wayne State U, Westat, The City University of New Y, University of California SD, et al. (2013) Comparing the Effectiveness of Two Alcohol+Adherence Interventions for HIV+ Youth.

614. Nct, The Hospital for Sick C (2013) Virtual Peer-to-Peer (VP2P) Support Mentoring for Chronic Pain: A Pilot RCT.

615. Nct, Grupo L, Fundação para a Ciência e a T, University of L (2013) Treatment of Pediatric Obesity (TOP) - A Multidisciplinary Approach Involving Adolescents and Their Peers.

616. Nct, Jury C, Merck S, amp, Dohme C (2013) A Pilot Study to Examine Efficacy of Peer Mentoring in Promoting Medication Adherence Among People Living With HIV/AIDS.

617. Nct, Karolinska I, Hanoi Medical U (2013) Assessment of Group Peer Support to Children With HIV in Vietnam.

618. Nct, University of P (2013) Influence of Peer Mentoring on Adolescent Contraceptive Decision Making.

619. Serra-Paya N, Ensenyat A, Real J, Castro-Vinuales I, Zapata A, et al. (2013) Evaluation of a family intervention programme for the treatment of overweight and obese children (Nereu Programme): a randomized clinical trial study protocol. BMC public health 13.

620. Huang KY, Nakigudde J, Calzada E, Boivin MJ, Ogedegbe G, et al. (2014) Implementing an early childhood school-based mental health promotion intervention in low-resource Ugandan schools: study protocol for a cluster randomized controlled trial. pp. 471.

621. Rosner R, Konig HH, Neuner F, Schmidt U, Steil R (2014) Developmentally adapted cognitive processing therapy for adolescents and young adults with PTSD symptoms after physical and sexual abuse: Study protocol for a randomized controlled trial.

622. Thorogood M, Goudge J, Bertram M, Chirwa T, Eldridge S, et al. (2014) The Nkateko health service trial to improve hypertension management in rural South Africa: study protocol for a randomised controlled trial. pp. 435.

623. Xie B, Ye XL, Sun ZL, Jia M, Jin H, et al. (2014) Peer support for patients with type 2 diabetes in rural communities of China: protocol for a cluster randomized controlled trial. pp. 747.

624. Irct201404167531N, Shiraz University of Medical S (2014) The effect of peer education on prevention of urinary tract infection.

625. Irct2013060912264N, Vice-Chancellor for Research Deputy in Tabriz University of Medical S (2014) The effect of peer coaches education on self care activities and its related confidence in adolescents with type 1 diabetes mellitus: a randomized controlled trial.

626. Irct2013112215484N, Vice-Chancellor for R, Technology of Shiraz University of Medical S (2014) Effect of coping strategies training on the use of coping strategies and quality of life in adolescents with thalassemia major.

627. Irct2014010715487N, Azad University Branch of Torbat-e J (2014) Investigating of Group Rational-Emotive-Behavior education efficacy on the juveniles.

628. Irct2014021613080N, Health Sciences Research C (2014) Effect of nutrition education on students nutritional status.

629. Irct2014042615650N, Vice- Chancellor for Health TUoMS, Center for Community Based Participatory Research Iorrb (2014) An educational intervention for improving responsibility for health, spiritual health and interpersonal relationships in girl students.

630. Isrctn, Al -Zaytoonah U (2014) Prevention of type 2 diabetes mellitus through educational intervention for adolescents at risk in high schools in Jordan.

631. Nct, University of Massachusetts B, Eunice Kennedy Shriver National Institute of Child H, Human D (2014) A Family-Based Weight Loss Intervention for Youth With Intellectual Disability.

632. Nct, University of R, National Institute of Mental H, Columbia U, Northwestern U, et al. (2014) Effectiveness Trial of Youth Suicide Prevention Delivered by Teen Peer Leaders.

633. Nct, Research VAOo, Development (2014) Veteran Peer-Assisted Computerized Cognitive Behavioral Therapy for Depression.

634. Nct, Seton Family of H (2014) A Psychoeducational Prevention for the Treatment of Atopic Dermatitis in Youth and Their Families.

635. Nct, Kaohsiung Medical University Chung-Ho Memorial H (2014) Prevention of Transmission of Sexually Transmitted Diseases in the Youth Through Effective Intervention.

636. Nct, Hai-Jun W (2014) Study on Obesity Intervention With Physical Exercise Among Students in Changping District, Beijing.

637. Nct, Virginia Commonwealth U, Children's Hospital F (2014) Study of a Structured Parent Intervention on Adolescent Weight Loss Modification Program.

638. Nct, University of Southern C (2014) Guided Imagery Lifestyle Intervention to Promote Health and Prevent Diabetes in Youth.

639. Nct, University of V, Eunice Kennedy Shriver National Institute of Child H, Human D (2014) Go Girls! Fitness Support Group Intervention Effectiveness Study.

640. Nct, Zhongda H (2014) Impact of Peer Support on Diabetes in China.

641. Nct, National D, Research Institutes I, National Institute on Drug A, Hanoi Medical U, et al. (2014) Implementation of a Sexual Health Intervention for Young Men Who Have Sex With Men (MSM) in Two Vietnamese Cities.

642. Nct, Harvard School of Public H, Caritas F, McGill U, Yale U, et al. (2014) Sub-Trial of the Youth Readiness Intervention (YRI): Treatment of Control Group and Addition of Stress Biomarkers.

643. Nct, Columbia U, National Institute of Mental H (2014) Peer-Led Healthy Lifestyle Program in Supportive Housing.

644. Nct, University of G, Kaufmännische K (2014) Effects of a Telephone Based Peer Support to Reduce Depressive Symptoms and Improve Social Support in Women With CHD.

645. Nct, Erasmus Medical C (2014) Working Memory Training for Dysphoric Students.

646. Nct, Hospital de Clinicas de Porto A, Yale U (2014) Randomized Controlled Trial With Use of Cognitive Training in Children and Adolescents With ADHD.

647. Nct, Rand (2014) Substance Use and Sexual Risk Reduction Intervention for Homeless Youth.

648. Nct, Faculdade de Motricidade H (2014) Effects of a Resistance and Stretching Training Program on Forward Head and Protracted Shoulder Posture Adolescents.

649. Nct, University of Massachusetts W, Brown U, Rhode Island H, The Miriam H, et al. (2014) Project HERA (Health, Education, and Relationship-building for Adolescents- Moms and Tots).

650. Nct, University Health Network T, Stony Brook U, York U (2014) Cardiac Rehabilitation Peer Mentorship.

651. Nct, University of California SF, National Institute on Drug A (2014) Tobacco Status Project: Social Media Intervention for Young Adult Smokers.

652. Nct, Colorado State U, Eunice Kennedy Shriver National Institute of Child H, Human D (2014) Learning to BREATHE: A Randomized Controlled Trial to Lower Diabetes Risk in Adolescent Girls.

653. Nct, Maastricht University Medical C (2014) Exercise Training in Healthy Young Men.

654. Nct, Massachusetts General H, National Institute of Mental H (2014) Enhancing Outcomes, Reducing Costs: Evaluating Peer Support for Mood Disorders.

655. Nct, Universitätsklinikum H-E, German Federal Ministry of E, Research (2014) Peer Support for Severe Mental Disorders.

656. Nct, University of M, National Institute of Mental H (2014) A Novel Intervention Promoting Eating Disorder Treatment Among College Students.

657. Nct, University of R, Johns Hopkins U, University of T (2014) Peer Led Asthma Self Management for Adolescents: PLASMA.

658. Nct, Eskisehir Osmangazi U (2014) Evaluation of the Effectiveness of Internet Based Nutrition and Physical Activity Education Programme in Adolescents.

659. Nct, Göteborg U, Södra Älvsborg H, Habilitation Skaraborg S (2014) Internet-based Support for Young People With ADHD and Autism - a Controlled Study.

660. Nct, Hospital de Clinicas de Porto A, Federal University of Rio Grande do S (2014) Aquatic and Land Physical Training Effects on Cardiometabolic Risk Factors in Adolescents With Overweight and Obesity.

661. Nct, Ohio State U (2014) A Skills-based RCT for Physical Activity Using Peer Mentors.

662. Nct, Possible, Brigham, Women's H (2014) Group Antenatal Care: The Power of Peers for Increasing Skilled Birth Attendance in Achham, Nepal.

663. Nct, University of M, National Institutes of H (2014) Peer Mentorship to Reduce Suicide Risk Following Psychiatric Hospitalization.

664. Nct, Group Health C (2014) Pilot Trial of Peer Support for Bipolar Disorder.

665. Nct, Connecticut Children's Medical C, Seattle Children's H (2014) Web-MAP Intervention for Youth With Sickle Cell Disease.

666. Ntr, Trimbos-institute - Netherlands Institute of Mental H, Addiction (2014) Effects of Rock and Water, a psycho-physical training for boys.

667. Boone P, Camara A, Eble A, Elbourne D, Fernandes S, et al. (2015) Remedial after-school support classes offered in rural Gambia (The SCORE trial): Study protocol for a cluster randomized controlled trial.

668. Friedrich RR, Caetano LC, Schiffner MD, Wagner MB, Schuch I (2015) Design, randomization and methodology of the TriAtiva Program to reduce obesity in school children in Southern Brazil. pp. 363.

669. Kumar S, Ray S, Mahapatra T, Gupta K, Mahapatra S, et al. (2015) Cardiovascular risk reduction intervention among school-students in Kolkata, West Bengal - The CRRIS study protocol. pp. 33-39.

670. Actrn, University Curtin U (2015) A pairwise randomised control trial of a peer-to-peer play-based intervention for children with Autism Spectrum Disorder to improve social play skills and pragmatic language.

671. Actrn, Professor David L, Nicole N (2015) Great Leaders, Active Students.- Evaluating the impact of a Peer Leadership Program for Grade 6 students on the movement skill competency and physical activity levels of students in Kindergarten, Grade 1 and 2.

672. Actrn, University University of N, Prof David L, Prof Philip M, Prof Ronald P, et al. (2015) The ATLAS (Active Teen Leaders Avoiding Screen-time) and NEAT (Nutrition and Enjoyable Activity for Teens) school-based health-related fitness programmes for adolescents.

673. Ctri, Dst Seed New D, Avinashilingam U (2015) Community Based Approach for Combating Anaemia Among Adolescent Girls in Rural Areas of Coimbatore District.

674. Irct2015010810426N, Hamadan University of Medical S (2015) Comparison the effect of menstrual health education with lecture and peer group method of on knowledge and practices among high school girls in Hamadan City , 2014.

675. Irct2015100624381N, Tehran University of Medical S (2015) The Effect of an Intervention on Preventive Behaviors of Domestic Violence in Female high school students.

676. Irct2015102520778N, Vice Chancellor for research of Urmia University of Medical S (2015) The Effect of Group Discussion on Adolescent Health Concerns.

677. Irct2015102824750N, Tehran University of Medical S (2015) Sexual education of female adolescents.

678. Isrctn, University of B (2015) A peer-led physical activity intervention for adolescent girls.

679. Isrctn, Newcastle U (2015) Screening and brief alcohol intervention to prevent risky drinking in young people aged 14-15 in a high school setting.

680. Isrctn, American University of B (2015) School-based waterpipe smoking cessation intervention program among Jordanian adolescents.

681. Isrctn, University of B (2015) Evaluating the Breakthrough Mentoring scheme: a feasibility pilot randomised control trial (RCT) with vulnerable adolescents deemed at risk of exclusion in a secondary school setting.

682. Nct, Sun Yat-sen U, Peking U, Central South U, Tianjin Medical U, et al. (2015) A National School-based Health Lifestyles Interventions Among Chinese Children and Adolescents Against Obesity.

683. Nct, Virginia Commonwealth U, Henrico County Public S, Virginia Department of A, Rehabilitative S, et al. (2015) A Collaborative Public/Private Employment Training and Placement Model ASD Transition Age Youth With Autism Spectrum Disorder (ASD).

684. Nct, Stanford U (2015) Social Motivation Intervention for Children With Autism Spectrum Disorder: Improving Peer Initiation.

685. Nct, University of Illinois at U-C, Penn State U (2015) Peer Education About Weight Steadiness.

686. Nct, Research VAOo, Development (2015) Evaluation of a Peer Coach-Led Intervention to Improve Pain Symptoms.

687. Nct, University of B, University of York DoHS, Birmingham, Solihull Mental Health NHSFT, et al. (2015) Family and Social Intervention for Young People.

688. Nct, City of Hope Medical C, National Cancer I (2015) Peer-to-Peer Support Program in Improving Quality of Life Outcomes in Patients With Gynecologic Cancer and Their Caregivers.

689. Nct, Assistance Publique Hopitaux De M (2015) Description of Socio-cognitive and Clinic Changes for Type 1 Diabetes Adolescents Cohort With Therapeutic Patient Education Program.

690. Nct, CoheroHealth, Icahn School of Medicine at Mount S (2015) Assessment of a Mobile Intervention to Increase Adherence to Asthma Medication Among Adolescents.

691. Nct, Children's Hospital B, American Foundation for Suicide P (2015) Brief Alcohol Intervention for Adolescents Who Have Attempted Suicide.

692. Nct, McGill U (2015) Transition Support Program for Young Adults With Autism Spectrum Disorders.

693. Nct, The University of Texas Health Science Center H, Paso del Norte Health F (2015) Teens Against Tobacco Use: A Model for Universal Tobacco Prevention and Youth Advocacy.

694. Nct, Centers for Disease C, Prevention, Ministry of E, Skills Development B, et al. (2015) An Assessment of an HIV Prevention Intervention (Project AIM) Among Junior Secondary School Students in Eastern Botswana.

695. Nct, Pontificia Universidade Católica do Rio Grande do S, Conselho Nacional de Desenvolvimento Científico e T, Fundação de Amparo à Pesquisa do Estado do Rio Grande do Sul B (2015) Interdisciplinary Intervention With Motivational Approach in Adolescents With Overweight and Obesity.

696. Nct, Virginia Polytechnic I, State U (2015) STEPS: Stepped Transition in Education Program for Students With ASD.

697. Nct, National Cancer I (2015) Acceptance and Commitment Training for Adolescents and Young Adults With Neurofibromatosis Type 1, Plexiform Neurofibromas, and Chronic Pain.

698. Nct, University of M, National Institute on D, Other Communication D (2015) Test of Hearing Health Education Programs for Farm and Rural Youth.

699. Nct, Elizabeth Glaser Pediatric AF, Ministry of H, Child Welfare Z, The Children's Investment Fund F (2015) Community-Based Peer Facilitator Intervention.

700. Nct, Florida International U (2015) Intervention for Teens With ADHD and Substance Use.

701. Nct, Boston U (2015) Testing Effectiveness of a Peer-Led Intervention to Enhance Community Integration.

702. Nct, Holland Bloorview Kids Rehabilitation H, Ability O, University of T, The Hospital for Sick C, et al. (2015) A Peer E-mentoring Intervention to Improve Employment.

703. Nct, University of Texas Southwestern Medical C, National Cancer I, Southern Methodist U, Parkland H, et al. (2015) Developing a Self-persuasion Intervention Promoting Adolescent HPV Vaccination: Feasibility Trial.

704. Nct, University of Texas Southwestern Medical C, National Cancer I, Southern Methodist U, Parkland H, et al. (2015) Developing a Self-persuasion Intervention Promoting Adolescent HPV Vaccination.

705. Nct, The University of Texas Health Science Center at San A, The Office of Adolescent H (2015) Evaluation of a Need to Know (N2K): A New Sex Education Program for High School Students.

706. Nct, University College D, University of B (2015) Emotion Recognition Training for Young People.

707. Nct, University of Texas at A, Ohio State U (2015) An Intervention to Promote Healthy Behaviors in Homeless Youth.

708. Nct, Qazvin University Of Medical S (2015) A Theory-based Sleep Intervention in Improving Sleep Quality in Adolescents.

709. Nct, Children's Hospital of P (2015) The P4 Intervention Study to Improve Adolescent Health.

710. Nct, Harvard School of Public H, Caritas F, World B, The City College of New Y (2015) Adapting Mental Health Interventions for War-Affected Youth Through Employment Programs.

711. Nct, Massachusetts General H, Foundation For Faces of C (2015) Resiliency Training for Adolescents With Craniofacial Conditions and Their Caregivers.

712. Nct, Ohio U, University of Kansas Medical C (2015) Interventions for Sanctioned Ohio University Students.

713. Nct, University of U, Center for Sepsis C, Care G, Zentrum für klinische Studien E, et al. (2015) Structured Psychoeducation for Unemployed Adolescents With Extreme Obesity in a Multicenter Observational Study Focusing on Feasibility.

714. Nct, University of S, University of L (2015) Sports, Education and Consumption of Substances in Adolescents.

715. Nct, Helsinki University Central H (2015) Motivational Interview in Adolescents With Poorly Controlled Type 1 Diabetes.

716. Nct, New York U (2015) Cornerstone: Boundary Spanning Case Management and Peer Support for Transition Age Youth With Mental Disorders.

717. Nct, National Health Promotion Associates I (2015) Testing a Multilevel Preventive Intervention in Youth Courts.

718. mnv5g RBR, Universidade Federal de São Paulo - Unifesp - São Paulo SPB, Ministério da Saúde - Distrito Federal DFB, Universidade Federal de São Paulo - Unifesp - São Paulo SPB (2015) Randomized Controlled Trial of the School Prevention Program for drug Unplugged (#Tamojunto) among Brazilian adolescents.

719. zp9n RBR, Escola de Nutrição da Universidade Federal da Bahia - Salvador BAB, Escola de Nutrição da Universidade Federal da Bahia - Salvador BAB (2015) Evaluation of the effects of a programme promoting adequate and healthy eating on adolescent health condiction: an interventional study.

720. Barbosa Filho VC, Lopes Ada S, Lima AB, de Souza EA, Gubert Fdo A, et al. (2015) Rationale and methods of a cluster-randomized controlled trial to promote active and healthy lifestyles among Brazilian students: the "Fortaleca sua Saude" program. BMC public health 15: 1212.

721. Woelk GB, Kieffer MP, Walker D, Mpofu D, Machekano R (2016) Evaluating the effectiveness of selected community-level interventions on key maternal, child health, and prevention of mother-to-child transmission of HIV outcomes in three countries (the ACCLAIM Project): A study protocol for a randomized controlled trial.

722. Lippman SA, Shade SB, Sumitani J, DeKadt J, Gilvydis JM, et al. (2016) Evaluation of short message service and peer navigation to improve engagement in HIV care in South Africa: Study protocol for a three-arm cluster randomized controlled trial. Trials 17 (1) (no pagination).

723. Actrn, Dr Carolyn S, Dr Ron R, Dr Danielle E (2016) The Chilled Plus Program: Helping Adolescents with Anxiety and Depression.

724. Actrn, Queensland University of T, Nil (2016) Theory-based intervention for smoking cessation among Chinese middle school students.

725. Chi CI, The Chinese University of Hong K (2016) Effect of Wellness Recovery Action Plan on mental wellbeing among college students who are at risk for depression or anxiety: A randomized controlled trial.

726. Ctri, Anusmitha S (2016) Attitude of teenage students towards premarital sex and knowledge regarding contraception.

727. Irct2015110324861N, Arak University of Medical S (2016) The effect of education on reduction of stress students.

728. Irct2015121525532N, Vice chancellor for research JondiShapour University of Medical S (2016) Effect of Family Psychoeducation on the burden of family care and self-care behaviors in adolescents with type 1 diabetes.

729. Irct2016011125954N, Ahvaz Jundishapur University ofMedical S (2016) Assessing the effect of a training program based on the PRECEDE -PROCEED Model on lifestyle of adolescents with beta thalassemia.

730. Isrctn, Institute of Education S (2016) Efficacy of home and school-based interventions for students with disruptive behavior.

731. Isrctn, The Chinese University of Hong K (2016) The efficacy of the internet for physical activity promotion among university students.

732. Isrctn, International Planned Parenthood Federation WHR (2016) Supporting adolescent mothers in El Salvador.

733. Nct, Human Development Research Foundation P, Duke U, Columbia U, University of L, et al. (2016) Thinking Healthy Program Peer Delivered Plus.

734. Nct, Institute NMPMR (2016) Peer Education Program for HIV/AIDS Related Sexual Behaviors of Secondary School Students.

735. Nct, Seoul National University Childrens H (2016) Effectiveness of an Anti-bullying Intervention for Adolescent Perpetrators.

736. Nct, The Center for Mind-Body M (2016) Mind-Body Skills Groups for Behavioral and Emotional Problems in War-Traumatized Male Adolescents in Gaza.

737. Nct, Institute NMPMR, Kaithal H (2016) Impact of Peer Education Program on Headache Disorders.

738. Nct, Coordinación de Investigación en Salud M, Hospital Infantil de Mexico Federico G, Universidad Nacional Autonoma de M (2016) Internet-based Intervention to Prevent Risky Sexual Behaviors in Mexican Adolescents.

739. Nct, University of Social S, Humanities W, University of Z, Free University of B, et al. (2016) Effects of Self-efficacy, Planning, and Self-efficacy+Planning Interventions on Body Fat Among Adolescents.

740. Nct, Rutgers TSUoNJ, National Cancer I (2016) Peer Mentoring in Promoting Follow-up Care Self-Management in Younger Childhood Cancer Survivors.

741. Nct, Rio de Janeiro State U, Universidade Federal do Rio de J (2016) Managing Adolescent Obesity at Local Level by Combining Primary and Secondary Intervention.

742. Nct, University of T (2016) Digital Health Game as an Intervention Supporting Tobacco-related Health Literacy in Early Adolescents.

743. Nct, Yale U, Kenyatta National H, Impact R, amp, et al. (2016) High-yield HIV Testing, Facilitated Linkage to Care, and Prevention for Female Youth in Kenya.

744. Nct, Vanderbilt U (2016) Check It! Positive Psychology Intervention to Improve Adherence in Adolescents With T1D.

745. Nct, Lawson Health Research I (2016) A Feasibility (Pilot) Study of an Innovative Non-pharmacological Intervention Program in at Risk Youth.

746. Nct, Universidad Santo T, Universidad de Santiago de C, Universidad de G, Universidad Pública de N, et al. (2016) Exercise Training and Hepatic Metabolism in Overweight/Obese Adolescent.

747. Nct, Johns Hopkins Bloomberg School of Public H (2016) A Peer-based Mobile-health Intervention to Increase Access and Adherence to Hepatitis C Treatment and HIV Viral Suppression.

748. g9q RBR, Universidade do Estado do Rio Grande do Norte - Mossoró RNB, Centro Municipal de Controle à Obesidade Jansen Jefferson Diógenes e Medeiros - Mossoró RNB (2016) Effects of weightlifting and aerobic training on bofy fat and laboratory blood tests in adolescents with overweight and obesity.

749. Amarasekara P, Katulanda P, De Silva PA (2013) A better way to reduce cardiovascular risk among patients with metabolic syndrome? study protocol for a hospital-based randomized controlled trial. Annals of Nutrition and Metabolism 63: 1359.

750. Leventhal KS, DeMaria LM, Gillham J, Andrew G, Peabody JW, et al. (2015) Fostering emotional, social, physical and educational wellbeing in rural India: The methods of a multi-arm randomized controlled trial of Girls First.

751. Isrctn, University of Z, University of B (2016) Research initiative to support the empowerment of girls.

752. Nct, University of Technology S, Jordan University of S, Technology (2013) Efficacy of School-Based, Peer-Led Asthma and Smoking Prevention Program on CO1 Levels and Smoking Behavior Among Early Adolescents in Jordan.

753. Austrian K, Muthengi E, Mumah J, Soler-Hampejsek E, Kabiru CW, et al. (2017) The Adolescent Girls Initiative-Kenya (AGI-K): study protocol. BMC public health. pp. 210.

754. ISRCTN43712527 (2017) GirlsRead! Zambia DREAMS Innovation.

755. NCT02888678 (2016) Evaluation of an Integrated Economic Strengthening and HIV Prevention Program for Vulnerable Youth in South Africa.

756. NCT02907125 (2016) Extended Evaluation of School-based Health Promotion Programmes in Bihar, India.

757. Sharif Ishak SIZ, Chin YS, Mohd Taib MN, Mohd Shariff Z (2016) School-based intervention to prevent overweight and disordered eating in secondary school Malaysian adolescents: a study protocol. BMC public health 16: 1101-1101.

758. SLCTR/2017/002 (2017) An intervention study to enable young people to act as change agents to reduce cardiovascular risk of their community in Sri Lanka.

759. Xavier LEDF, Bernardes-Souza B, Lisboa OC, Seeger W, Groneberg DA, et al. (2017) A Medical Student-Delivered Smoking Prevention Program, Education Against Tobacco, for Secondary Schools in Brazil: Study Protocol for a Randomized Trial. JMIR research protocols 6: e16-e16.

760. Merwe A, Dawes A Youth Violence: A Review of Risk Factors, Causal Pathways and Effective Intervention. Journal of Child and Adolescent Mental Health 19: 95-113.

761. N.A, World Health O, Anon Preventing HIV/AIDS in Young People: a Systematic Review of the Evidence from Developing Countries. WHO Technical Report Series ; N° 938: 348 p.-348 p.

762. Sule SS, Onayade AA Community-based antenatal and perinatal interventions and newborn survival. Nigerian Journal of Medicine 15: 108-114.

763. Oakley A, Fullerton D, Holland J, Arnold S, Francedawson M, et al. (1995) SEXUAL HEALTH-EDUCATION INTERVENTIONS FOR YOUNG-PEOPLE - A METHODOLOGICAL REVIEW. British Medical Journal 310: 158-162.

764. Cheng TO (1999) Teenage Smoking in China. Journal of Adolescence 22: 607-620.

765. Aggleton P, Rivers K (1999) Interventions for adolescents. Gibney, Laura [Ed]: 231-255.

766. Rutledge DN, Donaldson NE, Pravikoff DS (2000) Caring for families of patients in acute or chronic health care settings: part I -- principles. Online Journal of Clinical Innovations 3: 1-26 26p.

767. Mahomed K (2000) Iron supplementation in pregnancy. The Cochrane database of systematic reviews: CD000117-CD000117.

768. Gostin LO (2002) AIDS in Africa among women and infants: a human rights framework. The Hastings Center report 32: 9-10.

769. Fernandez S, Nebot M, Jane M (2002) The evaluation of effectiveness of scholastic programs in the prevention of consumption of tobacco, alcohol and cannabis: what do meta-analyses tell us? Revista espanola de salud publica 76: 175-187.

770. Van DE, Kulier R, Gulmezoglu AM, Villar J (2002) Vitamin A supplementation during pregnancy. The Cochrane database of systematic reviews: CD001996-CD001996.

771. Cameron G, Karabanow J (2003) The nature and effectiveness of program models for adolescents at risk for entering the formal child protection system. Child Welfare 82: 443-474 432p.

772. Speizer IS, Magnani RJ, Colvin CE (2003) The effectiveness of adolescent reproductive health interventions in developing countries: a review of the evidence (Structured abstract). pp. 324-348.

773. Gallant M, Maticka-Tyndale E (2004) School-based HIV prevention programs for African youth. Social Science & Medicine 58: 1337-1351 1315p.

774. Stephenson J, Obasi A (2004) HIV risk-reduction in adolescents. The Lancet 363: No Pagination Specified.

775. Kay-Hyon K, 노성덕 (2004) A Meta-Analysis on the Effectiveness of Peer Counseling Conducted in Korea. The Korea Journal of Youth Counseling 12: 3-10.

776. Mello EDd, Luft VC, Meyer F (2004) Childhood obesity--towards effectiveness. Jornal de Pediatria 80: 173-182.

777. Edejer TTT, Aikins M, Black R, Wolfson L, Hutubessy R, et al. (2005) Achieving the millennium development goals for health - Cost effectiveness analysis of strategies for child health in developing countries. British Medical Journal 331: 1177-1180.

778. Gates S, McCambridge J, Smith Lesley A, Foxcroft D (2006) Interventions for prevention of drug use by young people delivered in non-school settings. John Wiley & Sons, Ltd.

779. Larun L, Nordheim Lena V, Ekeland E, Hagen Kåre B, Heian F (2006) Exercise in prevention and treatment of anxiety and depression among children and young people. John Wiley & Sons, Ltd.

780. Mytton Julie A, DiGuiseppi C, Gough D, Taylor Rod S, Logan S (2006) School-based secondary prevention programmes for preventing violence. John Wiley & Sons, Ltd.

781. Casazza K, Ciccazzo M (2006) Improving the dietary patterns of adolescents using a computer-based approach. Journal of School Health 76: 43-46.

782. Grimshaw GM, Stanton A (2006) Tobacco cessation interventions for young people. Cochrane Database of Systematic Reviews: CD003289.

783. Kirby D, Obasi A, Laris BA (2006) The effectiveness of sex education and HIV education interventions in schools in developing countries. World Health Organization Technical Report Series 938: 103-150; discussion 317.

784. Hoffmann O, Boler T, Dick B, Ross DA, Dick B, et al. (2006) Achieving the global goals on HIV among young people most at risk in developing countries: young sex workers, injecting drug users and men who have sex with men. 938: 287-315.

785. Mahomed K (2006) WITHDRAWN: Iron supplementation in pregnancy. The Cochrane database of systematic reviews: CD000117-CD000117.

786. Maticka-Tyndale E, Brouillard-Coyle C, Ross DA, Dick B, Ferguson J (2006) The effectiveness of community interventions targeting HIV and AIDS prevention at young people in developing countries. 938: 243-285.

787. Saxena S, Jane-Llopis E, Hosman C (2006) Prevention of mental and behavioural disorders: implications for policy and practice. World psychiatry 5: 5-14.

788. Diareme S, Tsiantis J, Romer G, Tsalamanios E, Anasontzi S, et al. (2007) Mental health support for children of parents with somatic illness: a review of the theory and intervention concepts. Families, Systems & Health: The Journal of Collaborative Family HealthCare 25: 98-118 121p.

789. Kirby DB, Laris BA, Rolleri LA (2007) Sex and HIV education programs: their impact on sexual behaviors of young people throughout the world. Journal of Adolescent Health 40: 206-217 212p.

790. Gagnon Anita J, Sandall J (2007) Individual or group antenatal education for childbirth or parenthood, or both. John Wiley & Sons, Ltd.

791. Kristjansson B, Petticrew M, MacDonald B, Krasevec J, Janzen L, et al. (2007) School feeding for improving the physical and psychosocial health of disadvantaged students. John Wiley & Sons, Ltd.

792. Gakidou E, Oza S, Fuertes CV, Li AY, Lee DK, et al. (2007) Improving child survival through environmental and nutritional interventions: The importance of targeting interventions toward the poor. Journal of the American Medical Association 298: 1876-1887.

793. Connelly JB, Duaso MJ, Butler G (2007) A systematic review of controlled trials of interventions to prevent childhood obesity and overweight: a realistic synthesis of the evidence. Public Health 121: 510-517.

794. Foss AM, Hossain M, Vickerman PT, Watts CH (2007) A systematic review of published evidence on intervention impact on condom use in sub-Saharan Africa and Asia. Sexually Transmitted Infections 83: 510-516.

795. Johnson Wayne D, Diaz Rafael M, Flanders William D, Goodman M, Hill Andrew N, et al. (2008) Behavioral interventions to reduce risk for sexual transmission of HIV among men who have sex with men. John Wiley & Sons, Ltd.

796. Li M, Li S, Baur LA, Huxley RR (2008) A systematic review of school-based intervention studies for the prevention or reduction of excess weight among Chinese children and adolescents. Obesity Reviews 9: 548-559.

797. Joanna Briggs I (2008) Effective dietary interventions for overweight and obese children. Nursing Standard 22: 35-40.

798. Paul-Ebhohimhen VA, Poobalan A, van Teijlingen ER (2008) A systematic review of school-based sexual health interventions to prevent STI/HIV in sub-Saharan Africa. BMC public health 8: 4.

799. Graeff-Martins AS, Flament MF, Fayyad J, Tyano S, Jensen P, et al. (2008) Diffusion of efficacious interventions for children and adolescents with mental health problems. Journal of Child Psychology and Psychiatry 49: 335-352.

800. Bhutta ZA, Ahmed T, Black RE, Cousens S, Dewey K, et al. (2008) Maternal and Child Undernutrition 3 - What works? Interventions for maternal and child undernutrition and survival. Lancet 371: 417-440.

801. Shahmanesh M, Patel V, Mabey D, Cowan F (2008) Effectiveness of interventions for the prevention of HIV and other sexually transmitted infections in female sex workers in resource poor setting: a systematic review. Tropical Medicine & International Health 13: 659-679.

802. Sichieri R, de Souza RA (2008) Strategies for obesity prevention in children and adolescents. Cadernos de saude publica 24: S209-S223.

803. Stoltzfus RJ (2008) Research Needed to Strengthen Science and Programs for the Control of Iron Deficiency and Its Consequences in Young Children. Journal of Nutrition 138: 2542-2546.

804. Bolton P, Murray L, Bass J (2009) School based intervention improves PTSD symptoms in children affected by political violence. Evidence Based Mental Health 12: 47-47 41p.

805. Gonzalez-Suarez C, Worley A, Grimmer-Somers K, Dones V (2009) School-based interventions on childhood obesity a meta-analysis. American Journal of Preventive Medicine 37: 418-427 410p.

806. Li Y, Cai H, Li Q (2009) Community-based intervention for AIDS prevention. International Journal of Health Science 2: 266-270 265p.

807. Mikton C, Butchart A (2009) Child maltreatment prevention: a systematic review of reviews. Bulletin of the World Health Organization 87: 353-361 359p.

808. Mytton J, Towner E, Brussoni M, Gray S (2009) Unintentional injuries in school-aged children and adolescents: lessons from a systematic review of cohort studies. Injury Prevention (1353-8047) 15: 111-124 114p.

809. Wang Y, He G, Wang G (2009) Early problem behaviorals of children and its intervention study. Chinese Nursing Research 23: 945-947 943p.

810. Persson TJ, Rousseau C (2009) School-based interventions for minors in war-exposed countries: a review of targeted and general programmes. Torture : quarterly journal on rehabilitation of torture victims and prevention of torture 19: 88-101.

811. Medley A, Kennedy C, O'Reilly K, Sweat M (2009) Effectiveness of Peer Education Interventions for HIV Prevention in Developing Countries: A Systematic Review and Meta-Analysis. AIDS Education and Prevention 21: 181-206.

812. Acharya DR, Van Teijlingen ER, Simkhada P (2009) Opportunities and challenges in school-based sex and sexual health education in Nepal. Kathmandu University Medical Journal 7: 445-453.

813. Perez-Morales ME, Bacardi-Gascon M, Jimenez-Cruz A, Armendariz-Anguiano A (2009) [Randomized controlled school based interventions to prevent childhood obesity: systematic review from 2006 to 2009]. Archivos Latinoamericanos de Nutricion 59: 253-259.

814. Cernada GP (2009) Editor's foreword. International Quarterly of Community Health Education 29: 211-212.

815. Haider BA, Humayun Q, Bhutta ZA (2009) Effect of administration of antihelminthics for soil transmitted helminths during pregnancy. Cochrane Database of Systematic Reviews.

816. King E, De Silva M, Stein A, Patel V (2009) Interventions for improving the psychosocial well-being of children affected by HIV and AIDS. Cochrane Database of Systematic Reviews.

817. Agnihotri S, Lynn Keightley M, Colantonio A, Cameron D, Polatajko H (2010) Community integration interventions for youth with acquired brain injuries: a review. Developmental Neurorehabilitation 13: 369-382 314p.

818. Kesterton AJ, Cabral de Mello M (2010) Generating demand and community support for sexual and reproductive health services for young people: a review of the literature and programs. Reproductive Health 7: 12p-12p 11p.

819. Lopez LM, Hiller JE, Grimes DA (2010) Postpartum education for contraception: a systematic review. Obstetrical & Gynecological Survey 65: 325-331 327p.

820. Clasen Thomas F, Bostoen K, Schmidt W-P, Boisson S, Fung Isaac CH, et al. (2010) Interventions to improve disposal of human excreta for preventing diarrhoea. John Wiley & Sons, Ltd.

821. Hodnett Ellen D, Fredericks S, Weston J (2010) Support during pregnancy for women at increased risk of low birthweight babies. John Wiley & Sons, Ltd.

822. Lewin S, Munabi-Babigumira S, Glenton C, Daniels K, Bosch-Capblanch X, et al. (2010) Lay health workers in primary and community health care for maternal and child health and the management of infectious diseases. John Wiley & Sons, Ltd.

823. McCoy SI, Kangwende RA, Padian NS (2010) Behavior change interventions to prevent HIV infection among women living in low and middle income countries: a systematic review (Structured abstract). pp. 469-482.

824. Harrison A, Newell ML, Imrie J, Hoddinott G (2010) HIV prevention for South African youth: which interventions work? A systematic review of current evidence. BMC public health 10: 102.

825. Lazarus JV, Sihvonen-Riemenschneider H, Laukamm-Josten U, Wong F, Liljestrand J (2010) Systematic review of interventions to prevent the spread of sexually transmitted infections, including HIV, among young people in Europe. Croatian medical journal 51: 74-84.

826. Michielsen K, Chersich MF, Luchters S, De Koker P, Van Rossem R, et al. (2010) Effectiveness of HIV prevention for youth in sub-Saharan Africa: Systematic review and meta-analysis of randomized and nonrandomized trials. Aids 24: 1193-1202.

827. Lalor K, McElvaney R (2010) Child Sexual Abuse, Links to Later Sexual Exploitation/High-Risk Sexual Behavior, and Prevention/Treatment Programs. Trauma Violence & Abuse 11: 159-177.

828. Lassi ZS, Haider BA, Bhutta ZA (2010) Community-based intervention packages for reducing maternal and neonatal morbidity and mortality and improving neonatal outcomes. Cochrane Database of Systematic Reviews.

829. Michielsen K, Chersich MF, Luchters S, De Koker P, Van Rossem R, et al. (2010) Effectiveness of HIV prevention for youth in subSaharan Africa: systematic review and meta-analysis of randomized and nonrandomized trials. Aids 24: 1193-1202.

830. Murthy P, Subodh BN (2010) Current developments in behavioral interventions for tobacco cessation. Current Opinion in Psychiatry 23: 151-156.

831. Pattanaphesaj J, Teerawattananon Y (2010) Reviewing the evidence on effectiveness and cost-effectiveness of HIV prevention strategies in Thailand. BMC public health 10.

832. Reavley N, Jorm AF (2010) Prevention and early intervention to improve mental health in higher education students: a review. Early Intervention in Psychiatry 4: 132-142.

833. Ross DA (2010) Behavioural interventions to reduce HIV risk: what works? Aids 24: S4-S14.

834. Theodoratou E, Al-Jilaihawi S, Woodward F, Ferguson J, Jhass A, et al. (2010) The effect of case management on childhood pneumonia mortality in developing countries. International Journal of Epidemiology 39: 155-171.

835. van den Broek N, Kulier R, Gulmezoglu AM, Villar J (2010) WITHDRAWN: Vitamin A supplementation during pregnancy. The Cochrane database of systematic reviews: CD001996-CD001996.

836. 정순주 (2010) Research Trends in Education for Students with ADHD: A Review of Research from 1999 to 2008. Korean Journal of Special Education 44: 259-291.

837. (2011) Grand rounds: the opportunity for and challenges to malaria eradication. MMWR: Morbidity & Mortality Weekly Report 60: 476-480 475p.

838. Lau PWC, Lau EY, Wong DP, Ransdell L (2011) A systematic review of information and communication technology-based interventions for promoting physical activity behavior change in children and adolescents. Journal of medical Internet research 13: e48-e48 41p.

839. Medina-Blanco RI, Jiménez-Cruz A, Pérez-Morales ME, Armendáriz-Anguiano AL, Bacardí-Gascón M (2011) Intervention programs to promote physical activity in school children: systematic review]. Nutricion Hospitalaria 26: 265-270 266p.

840. Owen R, Kendrick D, Mulvaney C, Coleman T, Royal S (2011) Non-legislative interventions for the promotion of cycle helmet wearing by children. Cochrane Database of Systematic Reviews: N.PAG-N.PAG 1p.

841. Barlow J, Smailagic N, Bennett C, Huband N, Jones H, et al. (2011) Individual and group based parenting programmes for improving psychosocial outcomes for teenage parents and their children. John Wiley & Sons, Ltd.

842. Carson Kristin V, Brinn Malcolm P, Labiszewski Nadina A, Esterman Adrian J, Chang Anne B, et al. (2011) Community interventions for preventing smoking in young people. John Wiley & Sons, Ltd.

843. Carvalho Fernanda T, Gonçalves Tonantzin R, Faria Evelise R, Shoveller Jean A, Piccinini CA, et al. (2011) Behavioral interventions to promote condom use among women living with HIV. John Wiley & Sons, Ltd.

844. Deveaux L, Lunn S, Bain RM, Gomez P, Kelly T, et al. (2011) Focus on youth in the Caribbean: beyond the numbers. pp. [Epub].

845. Foxcroft David R, Tsertsvadze A (2011) Universal school-based prevention programs for alcohol misuse in young people. John Wiley & Sons, Ltd.

846. Khunpradit S, Tavender E, Lumbiganon P, Laopaiboon M, Wasiak J, et al. (2011) Non-clinical interventions for reducing unnecessary caesarean section. John Wiley & Sons, Ltd.

847. Reveiz L, Gyte Gillian ML, Cuervo Luis G, Casasbuenas A (2011) Treatments for iron-deficiency anaemia in pregnancy. John Wiley & Sons, Ltd.

848. Schroer-Gunther MA, Zhou M, Gerber A, Passon AM (2011) Primary tobacco prevention in China: a systematic review (Provisional abstract). pp. 2973-2980.

849. Waters E, de Silva-Sanigorski A, Burford Belinda J, Brown T, Campbell Karen J, et al. (2011) Interventions for preventing obesity in children. John Wiley & Sons, Ltd.

850. Bain-Brickley D, Butler LM, Kennedy GE, Rutherford GW (2011) Interventions to improve adherence to antiretroviral therapy in children with HIV infection. Cochrane database of systematic reviews (Online) 12: CD009513.

851. Samdal O, Rowling L (2011) Theoretical and Empirical Base for Implementation Components of Health-Promoting Schools. Health Education 111: 367-390.

852. Atun R, de Jongh TE, Secci FV, Ohiri K, Adeyi O, et al. (2011) Integration of priority population, health and nutrition interventions into health systems: systematic review. BMC public health 11: 780.

853. Napierala Mavedzenge SM, Doyle AM, Ross DA (2011) HIV prevention in young people in sub-Saharan Africa: a systematic review. Journal of Adolescent Health 49: 568-586.

854. Ritschel LA (2011) School-based group psychotherapy for at-risk adolescents. International Journal of Group Psychotherapy 61: 311-317.

855. Simoni JM, Nelson KM, Franks JC, Yard SS, Lehavot K (2011) Are peer interventions for HIV efficacious? A systematic review. AIDS and Behavior 15: 1589-1595.

856. Cooper Robbins SC, Ward K, Skinner SR (2011) School-based vaccination: a systematic review of process evaluations. Vaccine 29: 9588-9599.

857. Huang A-J (2011) Literature analysis of schistosomiasis control in Poyang Lake region. Zhongguo xue xi chong bing fang zhi za zhi = Chinese journal of schistosomiasis control 23: 330-332.

858. Williams ME, Thompson SC (2011) The Use of Community-Based Interventions in Reducing Morbidity from the Psychological Impact of Conflict-Related Trauma Among Refugee Populations: A Systematic Review of the Literature. Journal of Immigrant and Minority Health 13: 780-794.

859. Yang G-p, Wang Y-r, Zuo S-y, Zeng X-m (2011) Meta-analysis of intervention effects on obesity in Chinese pupils. Zhonghua yu fang yi xue za zhi [Chinese journal of preventive medicine] 45: 944-948.

860. Xiao Z, Li X, Mehrotra P (2012) HIV/Sexual Risk Reduction Interventions in China: A Meta-Analysis. AIDS Patient Care & Stds 26: 597-613 517p.

861. Gillies D, Taylor F, Gray C, O'Brien L, D'Abrew N (2012) Psychological therapies for the treatment of post-traumatic stress disorder in children and adolescents. John Wiley & Sons, Ltd.

862. Kendrick D, Young B, Mason-Jones Amanda J, Ilyas N, Achana Felix A, et al. (2012) Home safety education and provision of safety equipment for injury prevention. John Wiley & Sons, Ltd.

863. Klasen H (2012) What works where? A systematic review of child and adolescent mental health interventions for low and middle-income countries. pp. S248-s249.

864. Macdonald G, Higgins Julian PT, Ramchandani P, Valentine Jeffrey C, Bronger Latricia P, et al. (2012) Cognitive-behavioural interventions for children who have been sexually abused. John Wiley & Sons, Ltd.

865. Waters D, Theodoratou E, Campbell H, Rudan I, Chopra M (2012) Optimizing community case management strategies to achieve equitable reduction of childhood pneumonia mortality: an application of Equitable Impact Sensitive Tool (EQUIST) in five low- and middle-income countries (Provisional abstract). pp. 20402.

866. Witter S, Fretheim A, Kessy Flora L, Lindahl Anne K (2012) Paying for performance to improve the delivery of health interventions in low- and middle-income countries. John Wiley & Sons, Ltd.

867. Carney T, Myers B (2012) Effectiveness of early interventions for substance-using adolescents: findings from a systematic review and meta-analysis. Substance abuse treatment, prevention, and policy 7: 25.

868. Paech D, Coleman K, Mernagh PJ, Weston AR (2012) Assessing the cost effectiveness of public health interventions to prevent obesity: A systematic review of the effectiveness of 16 obesity prevention interventions. Value in Health 15 (7): A678.

869. Picot J, Shepherd J, Kavanagh J, Cooper K, Harden A, et al. (2012) Behavioural interventions for the prevention of sexually transmitted infections in young people aged 13-19 years: a systematic review. Health education research 27: 495-512.

870. Hastings RP, Robertson J, Yasamy MT (2012) Interventions for Children with Pervasive Developmental Disorders in Low and Middle Income Countries. Journal of Applied Research in Intellectual Disabilities 25: 119-134.

871. Emerson E, Yasamy MT, Saxena S (2012) Scaling up support for children with developmental disabilities in low- and middle-income countries. Journal of Applied Research in Intellectual Disabilities 25: 96-98.

872. Akinso OA, Rabiee F, Thomson K (2012) HIV risk-reduction interventions for adolescent's girls in sub-saharan Africa: a systematic review of qualitative studies relevant for future research and programme implementation. European Journal of Public Health 22: 179-180.

873. de Jongh T, Gurol-Urganci I, Vodopivec-Jamsek V, Car J, Atun R (2012) Mobile phone messaging for facilitating self-management of long-term illnesses. Cochrane Database of Systematic Reviews.

874. Homer CSE, Ryan C, Leap N, Foureur M, Teate A, et al. (2012) Group versus conventional antenatal care for women. Cochrane Database of Systematic Reviews.

875. Kain J, Uauy R, Concha F, Leyton B, Bustos N, et al. (2012) School-Based Obesity Prevention Interventions for Chilean Children During the Past Decades: Lessons Learned. Advances in Nutrition 3: 616S-621S.

876. Khoo S, Morris T (2012) Physical Activity and Obesity Research in the Asia-Pacific: A Review. Asia-Pacific Journal of Public Health 24: 435-449.

877. Martin RA, Bekirogullari Z (2012) Social and emotional learning research: Intervention studies for supporting adolescents in Turkey. 69: 1469-1476.

878. Noll M, Candotti CT, Vieira A (2012) Back school: systematic review of programs designed for schoolchildren in Brazil. Movimento 18: 265-291.

879. Tan JY, Huedo-Medina TB, Warren MR, Carey MP, Johnson BT (2012) A meta-analysis of the efficacy of HIV/AIDS prevention interventions in Asia, 1995-2009. Social Science & Medicine 75: 676-687.

880. Wariki WMV, Ota E, Mori R, Koyanagi A, Hori N, et al. (2012) Behavioral interventions to reduce the transmission of HIV infection among sex workers and their clients in low- and middle-income countries. Cochrane Database of Systematic Reviews.

881. Angela Shin-Yu L, Yen-Hua C, Jia-Ling T (2013) Effectiveness Evaluation of Healthy Lifestyle Interventions in Childhood Obesity Prevention: A Systematic Review. Journal of Nursing 60: 33-42 10p.

882. Guarino A, Bruzzese E, Lo Vecchio A, Dagan R, Tsolia M (2013) Definitions and Outcomes of Nutritional Interventions in Children with Respiratory Infections: The Approach of the COMMENT Initiative. Annals of Nutrition & Metabolism 63: 248-255 248p.

883. Kyung-Suk S, Young-Mi Y, Eun-Joo K (2013) Meta-analysis of the Effects of Obesity Management Program for Children. Child Health Nursing Research 19: 262-269 268p.

884. Marshall SJ, Simoes EJ, Eisenberg CM, Holub CK, Arredondo EM, et al. (2013) Weight-related child behavioral interventions in Brazil: a systematic review. American Journal of Preventive Medicine 44: 543-549 547p.

885. Reichow B, Servili C, Yasamy MT, Barbui C, Saxena S (2013) Non-specialist psychosocial interventions for children and adolescents with intellectual disability or lower-functioning autism spectrum disorders: a systematic review. PLoS Medicine 10: e1001572-e1001572 1001571p.

886. Sbruzzi G, Eibel B, Barbiero SM, Petkowicz RO, Ribeiro RA, et al. (2013) Educational interventions in childhood obesity: a systematic review with meta-analysis of randomized clinical trials. Preventive Medicine 56: 254-264 211p.

887. Sreevatsava M, Narayan KMV, Cunningham SA (2013) Evidence for interventions to prevent and control obesity among children and adolescents: its applicability to India. Indian Journal of Pediatrics 80: 115-122 118p.

888. Sydow K, Retzlaff R, Beher S, Haun MW, Schweitzer J (2013) The Efficacy of Systemic Therapy for Childhood and Adolescent Externalizing Disorders: A Systematic Review of 47 RCT. Family Process 52: 576-618 543p.

889. Chamberlain C, O'Mara-Eves A, Oliver S, Caird Jenny R, Perlen Susan M, et al. (2013) Psychosocial interventions for supporting women to stop smoking in pregnancy. John Wiley & Sons, Ltd.

890. Fellmeth Gracia LT, Heffernan C, Nurse J, Habibula S, Sethi D (2013) Educational and skills-based interventions for preventing relationship and dating violence in adolescents and young adults. John Wiley & Sons, Ltd.

891. Lopez Laureen M, Hilgenberg D, Chen M, Denison J, Stuart G (2013) Behavioral interventions for improving contraceptive use among women living with HIV. John Wiley & Sons, Ltd.

892. Pitt V, Lowe D, Hill S, Prictor M, Hetrick Sarah E, et al. (2013) Consumer-providers of care for adult clients of statutory mental health services. John Wiley & Sons, Ltd.

893. Thomas Roger E, McLellan J, Perera R (2013) School-based programmes for preventing smoking. John Wiley & Sons, Ltd.

894. van Ginneken N, Tharyan P, Lewin S, Rao Girish N, Meera SM, et al. (2013) Non-specialist health worker interventions for the care of mental, neurological and substance-abuse disorders in low- and middle-income countries. John Wiley & Sons, Ltd.

895. van-Velthoven Michelle H, Tudor Car L, Gentry S, Car J (2013) Telephone delivered interventions for preventing HIV infection in HIV-negative persons. John Wiley & Sons, Ltd.

896. Yonemoto N, Dowswell T, Nagai S, Mori R (2013) Schedules for home visits in the early postpartum period. John Wiley & Sons, Ltd.

897. Barry MM, Clarke AM, Jenkins R, Patel V (2013) A systematic review of the effectiveness of mental health promotion interventions for young people in low and middle income countries. BMC Public Health 13: 835.

898. Broutet N, Lehnertz N, Mehl G, Camacho AV, Bloem P, et al. (2013) Effective health interventions for adolescents that could be integrated with human papillomavirus vaccination programs. Journal of Adolescent Health 53: 6-13.

899. Coren E, Hossain R, Pardo Pardo J, Veras MM, Chakraborty K, et al. (2013) Interventions for promoting reintegration and reducing harmful behaviour and lifestyles in street-connected children and young people. Cochrane database of systematic reviews (Online) 2: CD009823.

900. Lobelo F, Garcia de Quevedo I, Holub CK, Nagle BJ, Arredondo EM, et al. (2013) School-based programs aimed at the prevention and treatment of obesity: Evidence-based interventions for youth in latin america. Journal of School Health 83: 668-677.

901. Lomotey M, Brown JL, DiClemente RJ (2013) Targeting STD/HIV prevention interventions for heterosexual male adolescents in North and Central America: A review. Current Pediatric Reviews 9: 376-382.

902. McQueston K, Silverman R, Glassman A (2013) The efficacy of interventions to reduce adolescent childbearing in low- and middle-income countries: A systematic review. Studies in Family Planning 44: 369-388.

903. Park BK, Calamaro C (2013) A systematic review of social networking sites: Innovative platforms for health research targeting adolescents and young adults. Journal of Nursing Scholarship 45: 256-264.

904. Tripney J, Hombrados J, Newman M, Hovish K, Brown C, et al. (2013) Technical and Vocational Education and Training (TVET) Interventions to Improve the Employability and Employment of Young People in Low- and Middle-Income Countries: A Systematic Review. Campbell Systematic Reviews 2013:9. Campbell Collaboration.

905. da Silva LS, Cotta RM, Rosa Cde O (2013) [Health promotion and primary prevention strategies to fight chronic disease: a systematic review]. Pan American Journal of Public Health 34: 343-350.

906. Joshi A, Amadi C (2013) Impact of water, sanitation, and hygiene interventions on improving health outcomes among school children. Journal Of Environmental & Public Health 2013: 984626.

907. Gupta R, Pandey A, John BM (2013) Home visits: A strategy to improve newborn survival. The Lancet 382: 1627.

908. Prost A, Colbourn T, Tripathy P, Osrin D, Costello A (2013) Analyses confirm effect of women's groups on maternal and newborn deaths. The Lancet 381: e15.

909. Betancourt TS, Meyers-Ohki SE, Charrow AP, Tol WA (2013) Interventions for Children Affected by War: An Ecological Perspective on Psychosocial Support and Mental Health Care. Harvard review of psychiatry 21: 70-91.

910. Das JK, Lassi ZS, Salam RA, Bhutta ZA (2013) Effect of community based interventions on childhood diarrhea and pneumonia: uptake of treatment modalities and impact on mortality. BMC public health 13.

911. Forman-Hoffman VL, Zolotor AJ, McKeeman JL, Blanco R, Knauer SR, et al. (2013) Comparative Effectiveness of Interventions for Children Exposed to Nonrelational Traumatic Events. Pediatrics 131: 526-539.

912. Hoehner CM, Ribeiro IC, Parra DC, Reis RS, Azevedo MR, et al. (2013) Physical Activity Interventions in Latin America Expanding and Classifying the Evidence. American Journal of Preventive Medicine 44: E31-E40.

913. Klasen H, Crombag A-C (2013) What works where? A systematic review of child and adolescent mental health interventions for low and middle income countries. Social psychiatry and psychiatric epidemiology 48: 595-611.

914. Lopez LM, Otterness C, Chen M, Steiner M, Gallo MF (2013) Behavioral interventions for improving condom use for dual protection. Cochrane Database of Systematic Reviews.

915. Makivic I, Kersnik J, Kolsek M (2013) MEASURES TO REDUCE RISKY AND HEAVY ALCOHOL DRINKING IN THE POPULATION OF STUDENTS: A SYSTEMATIC LITERATURE REVIEW. Zdravstveno Varstvo 52: 236-246.

916. Mutamba BB, van Ginneken N, Paintain LS, Wandiembe S, Schellenberg D (2013) Roles and effectiveness of lay community health workers in the prevention of mental, neurological and substance use disorders in low and middle income countries: a systematic review. BMC health services research 13.

917. Nagle BJ, Holub CK, Barquera S, Sanchez-Romero LM, Eisenberg CM, et al. (2013) Interventions for the treatment of obesity among children and adolescents in Latin America: a systematic review. Salud Publica de Mexico 55 Suppl 3: 434-440.

918. Walker CLF, Munos MK, Black RE (2013) Quantifying the indirect effects of key child survival interventions for pneumonia, diarrhoea, and measles. Epidemiology and Infection 141: 115-131.

919. 권경인 (2013) A Meta-Analysis on the Effects of Adolescent Stress Group Counseling Programs in Korea. Korean Journal Of Counseling And Psychotherapy 25: 41-62.

920. Mei-Chen S, Chia-Ling L, Lee-Ing T (2014) The Efficacy of E-Health Management on Weight Control in Adolescents: A Systematic Review. Journal of Nursing 61: 74-84 11p.

921. Vasconcellos F, Seabra A, Katzmarzyk P, Kraemer-Aguiar L, Bouskela E, et al. (2014) Physical Activity in Overweight and Obese Adolescents: Systematic Review of the Effects on Physical Fitness Components and Cardiovascular Risk Factors. Sports Medicine 44: 1139-1152 1114p.

922. Coppo A, Galanti Maria R, Giordano L, Buscemi D, Bremberg S, et al. (2014) School policies for preventing smoking among young people. John Wiley & Sons, Ltd.

923. Eccleston C, Palermo Tonya M, Williams Amanda CdC, Lewandowski Holley A, Morley S, et al. (2014) Psychological therapies for the management of chronic and recurrent pain in children and adolescents. John Wiley & Sons, Ltd.

924. Ersser Steven J, Cowdell F, Latter S, Gardiner E, Flohr C, et al. (2014) Psychological and educational interventions for atopic eczema in children. John Wiley & Sons, Ltd.

925. Gottschalk LB, Ortayli N (2014) Interventions to improve adolescents' contraceptive behaviors in low- and middle-income countries: a review of the evidence base (Provisional abstract). pp. 211-225.

926. Harrod Curtis S, Goss Cynthia W, Stallones L, DiGuiseppi C (2014) Interventions for primary prevention of suicide in university and other post-secondary educational settings. John Wiley & Sons, Ltd.

927. Jahanfar S, Howard Louise M, Medley N (2014) Interventions for preventing or reducing domestic violence against pregnant women. John Wiley & Sons, Ltd.

928. Langford R, Bonell Christopher P, Jones Hayley E, Pouliou T, Murphy Simon M, et al. (2014) The WHO Health Promoting School framework for improving the health and well-being of students and their academic achievement. John Wiley & Sons, Ltd.

929. Martin A, Saunders David H, Shenkin Susan D, Sproule J (2014) Lifestyle intervention for improving school achievement in overweight or obese children and adolescents. John Wiley & Sons, Ltd.

930. Patel N, Kellezi B, Williams Amanda CdC (2014) Psychological, social and welfare interventions for psychological health and well-being of torture survivors. John Wiley & Sons, Ltd.

931. Owusu-Addo E, Cross R (2014) The impact of conditional cash transfers on child health in low- and middle-income countries: a systematic review. International journal of public health 59: 609-618.

932. Schunter BT, Cheng WS, Kendall M, Marais H (2014) Lessons learned from a review of interventions for adolescent and young key populations in Asia Pacific and opportunities for programming. Journal of Acquired Immune Deficiency Syndromes 66: S186-S192.

933. Sherr L, Croome N, Bradshaw K, Parra Castaneda K (2014) A systematic review examining whether interventions are effective in reducing cognitive delay in children infected and affected with HIV. AIDS Care - Psychological and Socio-Medical Aspects of AIDS/HIV 26: S70-S77.

934. Stevens M, Kirsh B, Nixon SA (2014) Rehabilitation interventions for children living with HIV: a scoping review. Disability and rehabilitation 36: 865-874.

935. Wirtz AL, Pretorius C, Beyrer C, Baral S, Decker MR, et al. (2014) Epidemic impacts of a community empowerment intervention for HIV prevention among female sex workers in generalized and concentrated epidemics. PLoS ONE 9: e88047.

936. Baker-Henningham H (2014) The role of early childhood education programmes in the promotion of child and adolescent mental health in low- and middle-income countries. International Journal of Epidemiology 43: 407-433.

937. Jamil MS, Bauer HM, Hocking JS, Ali H, Wand H, et al. (2014) Chlamydia screening strategies and outcomes in educational settings: a systematic review. Sexually Transmitted Diseases 41: 180-187.

938. Purgato M, Gross AL, Jordans MJ, de Jong JT, Barbui C, et al. (2014) Psychosocial interventions for children exposed to traumatic events in low- and middle-income countries: study protocol of an individual patient data meta-analysis. Systems Review 3: 34.

939. Klasen H, Crombag A-C, Stolk K (2014) What works where? A systematic review of child and adolescent mental health interventions for low- and middle-income countries. Raynaud, Jean Philippe [Ed]: 125-155.

940. Naugle DA, Hornik RC (2014) Systematic review of the effectiveness of mass media interventions for child survival in low- and middle-income countries. Journal of health communication 19: 190-215.

941. Nyp SS (2014) School-based interventions for student mental health disorders. Journal of Developmental and Behavioral Pediatrics 35: 233.

942. Raynaud J-P, Hodes M, Gau SS-F (2014) From research to practice in child and adolescent mental health. 256.

943. Amaugo LG, Papadopoulos C, Ochieng BMN, Ali N (2014) The effectiveness of HIV/AIDS school-based sexual health education programmes in Nigeria: a systematic review. Health education research 29: 633-648.

944. Cai L, Wu Y, Wilson RF, Segal JB, Kim MT, et al. (2014) Effect of Childhood Obesity Prevention Programs on Blood Pressure. Circulation 129: 1832-1839.

945. Capurro D, Cole K, Echavarria MI, Joe J, Neogi T, et al. (2014) The Use of Social Networking Sites for Public Health Practice and Research: A Systematic Review. Journal of medical Internet research 16: 213-226.

946. Casemiro JP, Carvalho da Fonseca AB, Martins Secco FV (2014) Promoting health in school: reflections based on a review of school health in Latin America. Ciencia & Saude Coletiva 19: 829-840.

947. Fazel M, Patel V, Thomas S, Tol W (2014) Mental health interventions in schools in low-income and middle-income countries. Lancet Psychiatry 1: 388-398.

948. Fonner VA, Armstrong KS, Kennedy CE, O'Reilly KR, Sweat MD (2014) School Based Sex Education and HIV Prevention in Low- and Middle-Income Countries: A Systematic Review and Meta-Analysis. PLoS ONE 9.

949. Govindasamy D, Kranzer K, Ford N (2014) Strengthening the HIV cascade to ensure an effective future ART response in sub-Saharan Africa. Transactions of the Royal Society of Tropical Medicine and Hygiene 108: 1-+.

950. Govindasamy D, Meghij J, Negussi EK, Baggaley RC, Ford N, et al. (2014) Interventions to improve or facilitate linkage to or retention in pre-ART (HIV) care and initiation of ART in low- and middle-income settings - a systematic review. Journal of the International AIDS Society 17.

951. Grantham-McGregor SM, Fernald LCH, Kagawa RMC, Walker S, Black MM, et al. (2014) Effects of integrated child development and nutrition interventions on child development and nutritional status. 1308: 11-32.

952. Ickes MJ, McMullen J, Haider T, Sharma M (2014) Global School-Based Childhood Obesity Interventions: A Review. International Journal of Environmental Research and Public Health 11: 8940-8961.

953. Lieberman K, Huynh-Nhu L, Perry DF (2014) A systematic review of perinatal depression interventions for adolescent mothers. Journal of Adolescence 37: 1227-1235.

954. Mavedzenge SN, Luecke E, Ross DA (2014) Effective Approaches for Programming to Reduce Adolescent Vulnerability to HIV Infection, HIV Risk, and HIV-Related Morbidity and Mortality: A Systematic Review of Systematic Reviews. Jaids-Journal of Acquired Immune Deficiency Syndromes 66: S154-S169.

955. Moodley N, Gray G (2014) Global evidence reaffirms the case for routine HPV and potential HIV adolescent vaccination in South Africa. Future Virology 9: 207-220.

956. Noll M, Vieira A, Darski C, Candotti CT (2014) Back schools in Brazil: a review of the intervention methodology, assessment tools, and results. Revista Brasileira De Reumatologia 54: 51-58.

957. Roxby AC, Unger JA, Slyker JA, Kinuthia J, Lewis A, et al. (2014) A Lifecycle Approach to HIV Prevention in African Women and Children. Current Hiv/Aids Reports 11: 119-127.

958. Shephard DD (2014) Nonformal education for improving educational outcomes for street children and street youth in developing countries: A systematic review. International Journal of Social Welfare 23: 349-361.

959. Tsu VD, Cernuschi T, LaMontagne DS (2014) Lessons Learned From HPV Vaccine Delivery in Low-Resource Settings and Opportunities for HIV Prevention, Treatment, and Care Among Adolescents. Jaids-Journal of Acquired Immune Deficiency Syndromes 66: S209-S216.

960. Ye S, Yin L, Amico KR, Simoni JM, Vermund SH, et al. (2014) Efficacy of Peer-Led Interventions to Reduce Unprotected Anal Intercourse among Men Who Have Sex with Men: A Meta-Analysis. PLoS ONE 9.

961. 천정웅 (2014) A Study on Youth Mentoring. Journal of Future Oriented Youth Society 11: 117-135.

962. Akseer N, Kamali M, Husain S, Mirza M, Bakhache N, et al. (2015) Strategies to avert preventable mortality among mothers and children in the Eastern Mediterranean Region: new initiatives, new hope. Eastern Mediterranean Health Journal 21: 361-373 313p.

963. Bam K, Girase B (2015) Scenario of Adolescent Sexual and Reproductive Health with Opportunities for Information Communication and Technology Use in Selected South Asian Countries. Health Science Journal 9: 1-7 7p.

964. Hindin MJ, Bloem P, Ferguson J (2015) Effective Nonvaccine Interventions to Be Considered Alongside Human Papilloma Virus Vaccine Delivery. Journal of Adolescent Health 56: 10-18 19p.

965. Petrosino A, Morgan C, Fronius T, Tanner-Smith EE, Boruch RF (2015) What Works in Developing Nations to Get Children Into School or Keep Them There?: A Systematic Review of Rigorous Impact Studies. Research on Social Work Practice 25: 44-60 17p.

966. Augustincic Polec L, Petkovic J, Welch V, Ueffing E, Tanjong Ghogomu E, et al. (2015) Strategies to increase the ownership and use of insecticide-treated bednets to prevent malaria. John Wiley & Sons, Ltd.

967. Catling Christine J, Medley N, Foureur M, Ryan C, Leap N, et al. (2015) Group versus conventional antenatal care for women. John Wiley & Sons, Ltd.

968. Ejemot-Nwadiaro Regina I, Ehiri John E, Arikpo D, Meremikwu Martin M, Critchley Julia A (2015) Hand washing promotion for preventing diarrhoea. John Wiley & Sons, Ltd.

969. Fisher E, Law E, Palermo Tonya M, Eccleston C (2015) Psychological therapies (remotely delivered) for the management of chronic and recurrent pain in children and adolescents. John Wiley & Sons, Ltd.

970. Foxcroft David R, Moreira Maria T, Almeida Santimano Nerissa ML, Smith Lesley A (2015) Social norms information for alcohol misuse in university and college students. John Wiley & Sons, Ltd.

971. Hawton K, Witt Katrina G, Taylor Salisbury Tatiana L, Arensman E, Gunnell D, et al. (2015) Interventions for self-harm in children and adolescents. John Wiley & Sons, Ltd.

972. James Anthony C, James G, Cowdrey Felicity A, Soler A, Choke A (2015) Cognitive behavioural therapy for anxiety disorders in children and adolescents. John Wiley & Sons, Ltd.

973. Lassi Zohra S, Bhutta Zulfiqar A (2015) Community-based intervention packages for reducing maternal and neonatal morbidity and mortality and improving neonatal outcomes. John Wiley & Sons, Ltd.

974. Lindson-Hawley N, Thompson Tom P, Begh R (2015) Motivational interviewing for smoking cessation. John Wiley & Sons, Ltd.

975. Markova V, Norgaard A, Jørgensen Karsten J, Langhoff-Roos J (2015) Treatment for women with postpartum iron deficiency anaemia. John Wiley & Sons, Ltd.

976. Mbuagbaw L, Medley N, Darzi Andrea J, Richardson M, Habiba Garga K, et al. (2015) Health system and community level interventions for improving antenatal care coverage and health outcomes. John Wiley & Sons, Ltd.

977. Perry Amanda E, Neilson M, Martyn-St James M, Glanville Julie M, Woodhouse R, et al. (2015) Interventions for drug-using offenders with co-occurring mental illness. John Wiley & Sons, Ltd.

978. Rivas C, Ramsay J, Sadowski L, Davidson Leslie L, Dunne D, et al. (2015) Advocacy interventions to reduce or eliminate violence and promote the physical and psychosocial well-being of women who experience intimate partner abuse. John Wiley & Sons, Ltd.

979. Smith C, Gold J, Ngo Thoai D, Sumpter C, Free C (2015) Mobile phone-based interventions for improving contraception use. John Wiley & Sons, Ltd.

980. Thomas Roger E, Baker Philip RA, Thomas Bennett C, Lorenzetti Diane L (2015) Family-based programmes for preventing smoking by children and adolescents. John Wiley & Sons, Ltd.

981. Walsh K, Zwi K, Woolfenden S, Shlonsky A (2015) School-based education programmes for the prevention of child sexual abuse. John Wiley & Sons, Ltd.

982. Aggarwal S, Berk M (2015) Evolution of adolescent mental health in a rapidly changing socioeconomic environment: A review of mental health studies in adolescents in India over last 10 years. Asian Journal of Psychiatry 13: 3-12.

983. Lopez LM, Grey TW, Hiller JE, Chen M (2015) Education for contraceptive use by women after childbirth. The Cochrane database of systematic reviews 7: CD001863.

984. Till SR, Everetts D, Haas DM (2015) Incentives for increasing prenatal care use by women in order to improve maternal and neonatal outcomes. The Cochrane database of systematic reviews 12: CD009916.

985. Govindasamy D, Ferrand RA, Wilmore SM, Ford N, Ahmed S, et al. (2015) Uptake and yield of HIV testing and counselling among children and adolescents in sub-Saharan Africa: a systematic review. Journal of the International AIDS Society 18: 20182.

986. Kana MA, Doctor HV, Peleteiro B, Lunet N, Barros H (2015) Maternal and child health interventions in Nigeria: a systematic review of published studies from 1990 to 2014. BMC public health 15: 334.

987. MacPherson P, Munthali C, Ferguson J, Armstrong A, Kranzer K, et al. (2015) Service delivery interventions to improve adolescents' linkage, retention and adherence to antiretroviral therapy and HIV care. Tropical Medicine & International Health 20: 1015-1032.

988. Mureed S, Somronghtong R, Kumar R, Ghaffar A, Chapman RS (2015) Enhanced Immunization Coverage through Interventions for Childhood Cluster Diseases in Developing Countries. Journal of Ayub Medical College, Abbottabad: JAMC 27: 223-227.

989. Denno DM, Hoopes AJ, Chandra-Mouli V (2015) Effective strategies to provide adolescent sexual and reproductive health services and to increase demand and community support. Journal of Adolescent Health 56: S22-S41.

990. Lundgren R, Amin A (2015) Addressing intimate partner violence and sexual violence among adolescents: Emerging evidence of effectiveness. Journal of Adolescent Health 56: S42-S50.

991. Merrick J (2015) Child and adolescent health yearbook 2014. 475.

992. Kim E-K (2015) A Meta-Analysis and the Quality of Evidence of Single-Subject Research on Social Communication Interventions for Students with Autism Spectrum Disorders. Journal of the Korean Association for Persons with Autism 15: 69-99.

993. Morgan C, Petrosino A, Fronius T (2015) The impact of school vouchers in developing countries: A systematic review. International Journal of Educational Research 72: 70-79.

994. Pinzón Flórez CE, Díaz-Quijano DM, Yáñez Álvarez I, Catalina Mesa D (2015) Efectividad de los trabajadores comunitarios en medidas preventivas para salud maternal e infantil en países de bajos y medianos ingresos: revisión sistemática de la literatura and child health in low and middle income countries: systematic review of the literature. Revista Salud Uninorte 31: 309-328.

995. Salam RA, Haider BA, Humayun Q, Bhutta ZA (2015) Effect of administration of antihelminthics for soil-transmitted helminths during pregnancy. The Cochrane database of systematic reviews 6: CD005547-CD005547.

996. Weaver MS, Loennroth K, Howard SC, Roter DL, Lam CG (2015) Interventions to improve adherence to treatment for paediatric tuberculosis in low- and middle-income countries: a systematic review and meta-analysis. Bulletin of the World Health Organization 93: 700-711.

997. 이금진 (2015) Effects of Mentoring Program for children & Juvenile : A Meta-Analytic Review. Studies on Life and Culture 37: 173-214.

998. Carney T, Myers Bronwyn J, Louw J, Okwundu Charles I (2016) Brief school-based interventions and behavioural outcomes for substance-using adolescents. John Wiley & Sons, Ltd.

999. Coren E, Hossain R, Pardo Pardo J, Bakker B (2016) Interventions for promoting reintegration and reducing harmful behaviour and lifestyles in street-connected children and young people. John Wiley & Sons, Ltd.

1000. Lopez Laureen M, Grey Thomas W, Tolley Elizabeth E, Chen M (2016) Brief educational strategies for improving contraception use in young people. John Wiley & Sons, Ltd.

1001. Oringanje C, Meremikwu Martin M, Eko H, Esu E, Meremikwu A, et al. (2016) Interventions for preventing unintended pregnancies among adolescents. John Wiley & Sons, Ltd.

1002. Guerra PH, Da Silveira JAC, Salvador EP (2016) Physical activity and nutrition education at the school environment aimed at preventing childhood obesity: Evidence from systematic reviews. Jornal de Pediatria 92: 15-23.

1003. Abreu S, Miranda AAV, Murta SG (2016) Programas Preventivos Brasileiros: Quem Faz e como É Feita a Prevenção em Saúde Mental? Mental Health is Developed? en Salud Mental? Psico-USF 21: 163-177.

1004. Hennegan J, Montgomery P (2016) Do Menstrual Hygiene Management Interventions Improve Education and Psychosocial Outcomes for Women and Girls in Low and Middle Income Countries? A Systematic Review. PLoS ONE 11.

1005. Jordans MJD, Pigott H, Tol WA (2016) Interventions for Children Affected by Armed Conflict: a Systematic Review of Mental Health and Psychosocial Support in Low- and Middle-Income Countries. Current Psychiatry Reports 18.

1006. Leavy JE, Crawford G, Leaversuch F, Nimmo L, McCausland K, et al. (2016) A Review of Drowning Prevention Interventions for Children and Young People in High, Low and Middle Income Countries. Journal of community health 41: 424-441.

1007. Thornicroft G, Mehta N, Clement S, Evans-Lacko S, Doherty M, et al. (2016) Evidence for effective interventions to reduce mental-health-related stigma and discrimination. Lancet 387: 1123-1132.

1008. van Eijk AM, Sivakami M, Thakkar MB, Bauman A, Laserson KF, et al. (2016) Menstrual hygiene management among adolescent girls in India: a systematic review and meta-analysis. BMJ Open 6: e010290-e010290.

1009. Brown T, Smith S, Bhopal R, Kasim A, Summerbell CD (2015) Diet and physical activity interventions to prevent or treat obesity in South Asian children and adults: A systematic review and meta-analysis. Obesity Facts 8: 47.

1010. Brown T, Smith S, Bhopal R, Kasim A, Summerbell C (2015) Diet and physical activity interventions to prevent or treat obesity in south asian children and adults: A systematic review and meta-analysis. International Journal of Environmental Research and Public Health 12: 566-594.

1011. Abubakar A, Ssewanyana D, Newton CR (2016) A systematic review of research on autism spectrum disorders in sub-Saharan Africa. Behavioural Neurology 2016 (no pagination).

1012. Asnani Monika R, Quimby Kim R, Bennett Nadia R, Francis Damian K (2016) Interventions for patients and caregivers to improve knowledge of sickle cell disease and recognition of its related complications. Cochrane Database of Systematic Reviews: John Wiley & Sons, Ltd.

1013. Barbosa Filho VC, Minatto G, Mota J, Silva KS, de Campos W, et al. (2016) Promoting physical activity for children and adolescents in low- and middle-income countries: An umbrella systematic review. A review on promoting physical activity in LMIC. Preventive Medicine 88: 115-126.

1014. Brown FL, de Graaff AM, Annan J, Betancourt TS (2016) Annual Research Review: Breaking cycles of violence - a systematic review and common practice elements analysis of psychosocial interventions for children and youth affected by armed conflict. Journal of child psychology and psychiatry, and allied disciplines.

1015. Carvalho Coelho AC, Barretto Cardoso LS, de Souza-Machado C, Souza-Machado A (2016) The Impacts of Educational Asthma Interventions in Schools: A Systematic Review of the Literature. Canadian Respiratory Journal.

1016. Das JK, Salam RA, Lassi ZS, Khan MN, Mahmood W, et al. (2016) Interventions for Adolescent Mental Health: An Overview of Systematic Reviews. Journal of Adolescent Health 59: S49-S60.

1017. Fatusi AO (2016) Young people's sexual and reproductive health interventions in developing countries: Making the investments count. Journal of Adolescent Health 59: S1-S3.

1018. Genberg BL, Shangani S, Sabatino K, Rachlis B, Wachira J, et al. (2016) Improving engagement in the HIV care cascade: A systematic review of interventions involving people living with HIV/AIDS as peers. AIDS and Behavior 20: 2452-2463.

1019. Gillies D, Maiocchi L, Bhandari Abhishta P, Taylor F, Gray C, et al. (2016) Psychological therapies for children and adolescents exposed to trauma. Cochrane Database of Systematic Reviews: John Wiley & Sons, Ltd.

1020. Goudet S, Griffiths P, Bogin B, Madise N (2017) Interventions to tackle malnutrition and its risk factors in children living in slums: a scoping review. Annals of Human Biology 44: 1-10.

1021. Hetrick Sarah E, Cox Georgina R, Witt Katrina G, Bir Julliet J, Merry Sally N (2016) Cognitive behavioural therapy (CBT), third-wave CBT and interpersonal therapy (IPT) based interventions for preventing depression in children and adolescents. Cochrane Database of Systematic Reviews: John Wiley & Sons, Ltd.

1022. Hindin MJ, Kalamar AM, Thompson T-A, Upadhyay UD (2016) Interventions to prevent unintended and repeat pregnancy among young people in low- and middle-income countries: A systematic review of the published and gray literature. Journal of Adolescent Health 59: S8-S15.

1023. Hofmann SG, Doan SN, Sprung M, Wilson A, Ebesutani C, et al. (2016) Training children's theory-of-mind: A meta-analysis of controlled studies. Cognition 150: 200-212.

1024. Kalamar AM, Lee-Rife S, Hindin MJ (2016) Interventions to Prevent Child Marriage Among Young People in Low- and Middle-Income Countries: A Systematic Review of the Published and Gray Literature. Journal of Adolescent Health 59: S16-S21.

1025. Kanters S, Park JJ, Chan K, Ford N, Forrest J, et al. (2016) Use of peers to improve adherence to antiretroviral therapy: a global network meta-analysis. Journal of the International AIDS Society 19: 21141-21141.

1026. Lopez LM, Bernholc A, Chen M, Tolley EE (2016) School-based interventions for improving contraceptive use in adolescents. Cochrane Database of Systematic Reviews 2016 (6) (no pagination).

1027. Lopez LM, Grey TW, Tolley EE, Chen M (2016) Brief educational strategies for improving contraception use in young people. Cochrane Database of Systematic Reviews 2016 (3) (no pagination).

1028. Machado AP, Lima BM, Laureano MG, Silva PHB, Tardin GP, et al. (2016) Educational strategies for the prevention of diabetes, hypertension, and obesity. Revista da Associacao Medica Brasileira 62: 800-808.

1029. Mason-Jones AJ, Sinclair D, Mathews C, Kagee A, Hillman A, et al. (2017) School-based interventions for preventing HIV, sexually transmitted infections, and pregnancy in adolescents. Cochrane database of systematic reviews (online).

1030. Morina N, Malek M, Nickerson A, Bryant RA (2017) Psychological interventions for post-traumatic stress disorder and depression in young survivors of mass violence in low- and middle-income countries: meta-analysis. The British journal of psychiatry : the journal of mental science.

1031. Mukamana O, Johri M (2016) What is known about school-based interventions for health promotion and their impact in developing countries? A scoping review of the literature. Health Education Research 31: 587-602.

1032. O'Sullivan C, Bosqui T, Shannon C (2016) Psychological interventions for children and young people affected by armed conflict or political violence: a systematic literature review. Intervention-International Journal of Mental Health Psychosocial Work and Counselling in Areas of Armed Conflict 14: 142-164.

1033. Orton E, Whitehead J, Mhizha-Murira J, Clarkson M, Watson MC, et al. (2017) School-based education programmes for the prevention of unintentional injuries in children and young people. Cochrane database of systematic reviews (online).

1034. Salam RA, Arshad A, Das JK, Khan MN, Mahmood W, et al. (2016) Interventions to Prevent Unintentional Injuries Among Adolescents: A Systematic Review and Meta-Analysis. Journal of Adolescent Health 59: S76-S87.

1035. Salam RA, Faqqah A, Sajjad N, Lassi ZS, Das JK, et al. (2016) Improving Adolescent Sexual and Reproductive Health: A Systematic Review of Potential Interventions. Journal of Adolescent Health 59: S11-S28.

1036. Stoner L, Rowlands D, Morrison A, Credeur D, Hamlin M, et al. (2016) Efficacy of Exercise Intervention for Weight Loss in Overweight and Obese Adolescents: Meta-Analysis and Implications. Sports Medicine 46: 1737-1751.

1037. Vally Z, Abrahams L (2016) The effectiveness of peer-delivered services in the management of mental health conditions: A meta-analysis of studies from low- and middle-income countries. International Journal for the Advancement of Counselling 38: 330-344.

1038. Watters C, O'Callaghan P (2016) Mental health and psychosocial interventions for children and adolescents in street situations in low- and middle-income countries: A systematic review. Child Abuse & Neglect 60: 18-26.

1039. Stanton BF, Li X, Kahihuata J, Fitzgerald AM, Neumbo S, et al. (1998) Increased protected sex and abstinence among Namibian youth following a HIV risk-reduction intervention: a randomized, longitudinal study. pp. 2473-2480.

1040. Fitzgerald AM, Stanton BF, Terreri N, Shipena H, Li X, et al. (1999) Use of Western-based HIV risk-reduction interventions targeting adolescents in an African setting. pp. 52-61.

1041. Dick J, Clarke M, Zyl H, Daniels K (2007) Primary health care nurses implement and evaluate a community outreach approach to health care in the South African agricultural sector. pp. 383-390.

1042. Talukder K, Talukder MQ, Farooque MG, Khairul M, Sharmin F, et al. (2013) Controlling scabies in madrasahs (Islamic religious schools) in Bangladesh. pp. 83-91.

1043. Adam MB (2014) Effectiveness trial of community-based I Choose Life-Africa human immunodeficiency virus prevention program in Kenya. pp. 645-648.

1044. Chen Y, Shen WW, Gao K, Lam CS, Chang WC, et al. (2014) Effectiveness RCT of a CBT Intervention for Youths Who Lost Parents in the Sichuan, China, Earthquake. Psychiatric Services 65: 259-262.

1045. Puffer ES, Green EP, Sikkema KJ, Broverman SA, Ogwang-Odhiambo RA, et al. (2016) A church-based intervention for families to promote mental health and prevent HIV among adolescents in rural Kenya: Results of a randomized trial. Journal of Consulting and Clinical Psychology 84: 511-525.

1046. Perry CL, Grant M (1988) Comparing peer-led to teacher-led youth alcohol education in four countries. (Australia, Chile, Norway and Swaziland.). pp. 322-326.

1047. Perry CL, Grant M, Ernberg G, Florenzano RU, Langdon MC, et al. (1989) WHO Collaborative Study on Alcohol Education and Young People: outcomes of a four-country pilot study. pp. 1145-1171.

1048. Catani C, Kohiladevy M, Ruf M, Schauer E, Elbert T, et al. (2009) Treating children traumatized by war and Tsunami: a comparison between exposure therapy and meditation-relaxation in North-East Sri Lanka. pp. 22.

1049. Ghrayeb FAW, Rusli MA, Al Rifai A, Ismail MI (2013) Effectiveness of nutrition education intervention among high school students in Tarqumia, Palestine. pp. 787-792.

1050. Al-Sheyab N, Alomari M, Shah S, Gallagher R (2015) 'Class smoke-free' pledge impacts on nicotine dependence in male adolescents: A cluster randomized controlled trial. pp. 255-256.

1051. Ssewamala FM, Karimli L, Torsten N, Wang JS-H, Han C-K, et al. (2016) Applying a family-level economic strengthening intervention to improve education and health-related outcomes of school-going AIDS-orphaned children: Lessons from a randomized experiment in Southern Uganda. Prevention Science 17: 134-143.

1052. Al-sheyab NA, Alomari MA, Shah S, Gallagher R (2016) "Class smoke-free" pledge impacts on nicotine dependence in male adolescents: A cluster randomized controlled trial. Journal of Substance Use 21: 566-574.

1. These are the studies we excluded from our initial search (up to 9^th^ March 2017). [↑](#footnote-ref-1)
