# Supplementary material for: Peer-facilitated community-based interventions for adolescent health in low- and middle-income countries: A systematic review
Source: PLoS One. 2019 Jan 23;14(1):e0210468. doi: 10.1371/journal.pone.0210468 (PMC6343892; doi:10.1371/journal.pone.0210468)
Supplement: S1 Table — (DOCX) [file pone.0210468.s003.docx]

**S1 table: Articles of potentially eligible registered studies or study protocols for which we did not find published results**

|  | **Title** | **Setting** | **Participants** | **Details of the intervention** | **Outcomes** |
| --- | --- | --- | --- | --- | --- |
| 1 | Scientific evaluation of peer education and STD treatment to reduce the spread of HIV in Zimbabwe[[1](#_ENREF_1)] | Zimbabwe | Child, adult, senior | Community-based peer education and condom distribution combined with improved treatment of sexually transmitted infections | HIV incidence at the community level  Self-reported genital ulcers  Self-reported urethral or genital discharge  Sexually transmitted infection treatment effectiveness (self-reported cessation of symptoms) Sexual behaviour change (sexual debut, sexual partner change, non-regular partners, unprotected sex with regular and casual partners)  Health-seeking behaviour change  HIV/AIDS knowledge |
| 2 | Impact on student health status of a training program of university student as health promoters[[2](#_ENREF_2)]/ A randomized control trial: training program of university students as health promoters[[3](#_ENREF_3)] | Mexico | Health science university students | E-learning course for students to train them as health promoters on topics such as healthy eating, physical activity, addiction prevention, conflict management and self-esteem. | Healthy life style questionnaire  Prevalence of overweight, obesity, underweight  Prevalence of depression  Prevalence of addictions |
| 3 | Preventing early and unintended pregnancy in Vanuatu: a community-based intervention targeting adolescents[[4](#_ENREF_4)] | Vanuatu | Males and females aged 15-19 years | Trained peer educators will conduct adolescent participatory group workshops and individual sessions in order to: improve knowledge and attitudes towards pregnancy prevention; address misconceptions about contraception; promote voluntary contraception; and build interpersonal and negotiation skills. In addition, individual sessions will also provide personalised information, counselling and referral to youth-friendly health services. | Knowledge of reproduction and contraception  Attitude towards contraception  Self-reported use of any modern method of contraception at last sex  Contraceptive self-efficacy scale score |
| 4 | Evaluating a Youth-Focused Economic Empowerment Approach to HIV Treatment Adherence[[5](#_ENREF_5)] | Uganda | AIDS-orphaned adolescents | Matched savings accounts/child development accounts (CDAs) for the adolescents held in a local bank.  Financial education and workshops on asset-building, future planning, and protection from risks  Mentorship from a young adult/near-peer  Family-based microenterprise development training  Medical event monitoring system | Adherence to HIV treatment  Protective health behaviours (financial/economic stability, sexual risk-taking behaviour, personal beliefs about HIV medication, hopelessness, future plans and aspirations, adherence self-efficacy)  Cost-effectiveness |
| 5 | Microfinance and Health Intervention Trial for Youth in Dar es Salaam, Tanzania[[6](#_ENREF_6)] | Tanzania | Individuals aged 15 years or above and registered as a member of a ‘camp’ – i.e. a social group or club of mostly men | Microfinance and health leadership: participants receive small loans and business training. Nominated camp leaders will receive health leadership training on prevention of HIV, risk behaviours and gender-based violence perpetration. Leaders will then pass their knowledge on to other camp members. | Incidence of new sexually transmitted infections  Proportion of men reporting perpetration of physical, sexual or psychological partner violence at 12 months and 30 months |
| 6 | Evaluation of an Intervention for Adolescent Girls in Karnataka[[7](#_ENREF_7)]/ Supporting adolescent girls to stay in school, reduce child marriage and reduce entry into sex work as HIV risk prevention in north Karnataka, India: protocol for a cluster randomised controlled trial[[8](#_ENREF_8)] | India | Females aged 13-15 years | Education enhancement intervention: teacher training and academic support to students, addressing gender-related and skill-based haps among teachers; training local female teachers; increasing the knowledge of secondary school teachers; decreasing household economic pressure to withdraw girls from school; building appreciation and value of girls’ education; building family and community accountability for school attendance; creating safe spaces for girls; strengthening girls’ agency; and engaging boys in promoting and supporting girls’ education. | Delayed entry into sex work  Delayed sexual debut  Increased age at marriage |
| 7 | Promoting sexual and reproductive health among adolescents in southern and eastern Africa (PREPARE): project design and conceptual framework[[9](#_ENREF_9)] | South Africa, Uganda, Tanzania | School students aged 12-14 years | Two school-based comprehensive community prevention interventions and two more focussed school-based interventions to reduce STDs and unwanted pregnancies by changing sexual and reproductive behaviour and determinants of such behaviour. | Incidence of sexual debut (South Africa, Tanzania)  Behavioural beliefs related to sexual behaviours (South Africa)  Frequency of parent-child sexuality-related communication (Uganda) |
| 8 | Efficacy of peer education on HIV-related high risk behaviours[[10](#_ENREF_10)] | Iran | Females? 15-25 years from ‘malfunctioning families’ | HIV/AIDS prevention education delivered by peer educators | HIV-related high risk behaviours  Knowledge, attitudes and practice |
| 9 | Improving Adherence Among HIV+ Rwandan Youth: A TI-CBTe Indigenous Leader Model[[11](#_ENREF_11)] | Rwanda | Males and females aged 14-21 years | Group-based culturally adapted, ‘trauma-informed’ cognitive behavioural therapy intervention led by indigenous youth leaders. The intervention includes psychosocial health education, relaxation training, cognitive restructuring, adherence barriers and caregiver psycho-education. | ART adherence behaviour composite self report  Health care utilisation  HIV/AIDS/Sexually transmitted infection risk |
| 10 | Development and evaluation of a school-based smoking prevention intervention for 13-14 year old adolescents in Malaysia[[12](#_ENREF_12)] | Malaysia | Form 1 school students aged 13-14 years | A selection of students will be trained as anti-smoking peer supporters during a three-day course involving videos, talks and discussions, role plays and team building exercises. Peer supporters will then be asked to have informal anti-smoking conversations with their classmates. | Self-reported smoking  Peer educators: smoking-related knowledge and attitudes  All students: proportion of students who have had a one-to-one conversation about smoking; questionnaire on attitudes, subjective norms, perceived behavioural control and smoking intention |
| 11 | A Cluster-randomized Trial to Assess a Sexual Assault Prevention Intervention in Adolescents in Nairobi, Kenya[[13](#_ENREF_13)] | Kenya | Males and females aged 11-15 years | Two interventions: (i) the 12-hr ‘IMPower’ self defense course to empower girls to avoid risky situations, advocate for themselves and defend themselves from attack if necessary; (ii) the 12-hr ‘Source of Strength’ curriculum for boys to promote gender equality, develop positive masculinity and teach safe and effective bystander intervention techniques | Sexual assault incidence  Physical violence incidence |
| 12 | Creating opportunities through mentorship, parental involvement, and safe spaces (COMPASS) program: multi-country study protocol to protect girls from violence in humanitarian settings[[14](#_ENREF_14)] | Democratic Republic of Congo (DRC), Ethiopia, Pakistan | Females aged 10-14 years (DRC), females aged 13-19 years (Ethiopia) | Facilitated sessions for girls focusing on self-confidence, building friendships, communication, problem solving, puberty and reproduction, gender-based violence and creating healthy relationships to prepare girls for making decisions about relationships including their ability to negotiate sex, and understand their self-worth. Adolescents will also create plans to minimise risk of entering relationships where they may experience abuse, violence or exploitation  Caregivers will participate in monthly discussion groups to increase knowledge about the needs of adolescent girls  Engagement and training of health providers to improve the quality of services for girl survivors of violence | Girl outcomes:  Past year sexual violence  Past year positive interpersonal relationships  Past year early marriage  Past year physical violence and emotional and verbal abuse  Self-esteem  Gender equitable norms  Hope and future orientation  Accepting attitudes towards domestic violence  Caregiver outcomes:  Parental acceptance  Accepting attitudes of negative discipline  Gender equitable norms |
| 13 | The Peer Support Intervention – Supporting HIV Positive Adolescents in Zimbabwe to Improve HIV Care Continuum Outcomes[[15](#_ENREF_15)] | Zimbabwe | Males and females aged 10-24 years | Monthly peer support intervention group facilitated by professional HIV counsellor and Peer Support Intervention counsellor. Regular counselling through home visits and SMS messages. Weekly home visits. Daily WhatsApp messages enquiring about well being from Community Adolescent Treatment Supporters. 3-session intervention for caregivers to better support their adolescents. | Virologic suppression rate  Frequency of drug resistant mutations  Prevalence of psychological distress  Viral load, DBS and hair (tenofovir concentrations)  Detection of drug resistance |
| 14 | Peer Groups for Healthy Pregnancy & HIV Prevention for Young Malawian Women[[16](#_ENREF_16)] | Malawi | Females aged 15-20 years | Community-based peer groups to increase young women’s preconception behaviours and optimise their reproductive health. 8 group sessions led by community young women focused on reproductive health: HIV, STI and unintended pregnancy prevention. Skill building for self-efficacy. | Change in unprotected sex  Change in having had an HIV test  Change in having STI symptoms  Change in unintended pregnancy |
| 15 | An evaluation of the effectiveness of peer-education in preventing new HIV infections among adolescents in Northern Malawi[[17](#_ENREF_17)] | Malawi | Males and females aged 9-20 years | Peer education through fortnightly sessions | Number of sexual partners  Psychosocial determinants of sexual practices (self-esteem and self concept)  Attitudes, beliefs and practices towards abstinence, faithfulness to partner and condom use  Age at sexual debut  Secondary abstinence  Knowledge  Self-efficacy  Reported sexual behaviour  Attitude to youth services and service providers  Accepting attitudes and reporting attitudes against stigma or discrimination of people with HIV/AIDS  Adherence to biomedical protocols (VCT, male circumcision, seeking STI treatment) |
| 16 | Effectiveness of a girls’ empowerment programme on early childbearing, marriage and school dropout among adolescent girls in rural Zambia: study protocol for a cluster randomised trial[[18](#_ENREF_18)] | Zambia | Girls enrolled in grade 7 | Economic support consisting of monthly cash transfer to the girl, an annual cash grant to her parents/guardians and payment of school fees for girls who enrol in grades 8 and 9; economic support combined with community-orientated intervention including community and parent meetings to promote supportive community norms around education for girls and establishment of youth clubs to provide comprehensive sexual and reproductive health education. | Incidence of births within 8 months of the end of the intervention period  Incidence of births before girls’ 18^th^ birthday  Proportion of girls taking grade 9 exam  Incidence of pregnancies within 2 years of the end of the intervention period  Incidence of births within 2 years of the end of the intervention period  Incidence of pregnancies before girls’ 16^th^ birthday  Incidence of births before girls’ 16^th^ birthday  Incidence of pregnancies before girls’ 18^th^ birthday  Socioeconomic inequality in proportion of girls giving birth before 18^th^ birthday  Proportion of girls married and/or cohabiting before 16^th^ birthday  Proportion of girls married and/or cohabiting before 18^th^ birthday  Socioeconomic inequality in proportion of girls married and/or cohabiting before 18^th^ birthday  Proportion of girls enrolling in grade 8  Socioeconomic inequality in proportion of girls participating in grade 9 exam  Proportion of girls enrolling in grade 10  Girls’ average grade 9 exam score for English, maths and science  School attendance of grade 8 girls  School attendance of grade 9 girls  Proportion of adolescents sexually active in last 4 weeks  Proportion of adolescent girls currently using modern contraceptives  Knowledge of modern contraceptives  Perceived community norms regarding modern contraceptive use among unmarried adolescent girls  Perceived community norms regarding early marriage among girls  Perceived community norms regarding adolescent pregnancy among girls  Perceived community norms regarding education among girls  Proportion of girls currently employed or self-employed |
| 17 | Vijana Vijiweni II: a cluster-randomized trial to evaluate the efficacy of a microfinance and peer health leadership intervention for HIV and intimate partner violence prevention among social networks of young men in Dar es Salaam[[19](#_ENREF_19)] | Dar es Salaam | Young men aged 15+ | Microfinance intervention including business skills training, formation of loan groups, applications for loans, distribution of loans, weekly repayment sessions, ongoing access to credit and monitoring repayments.  Peer health leader component: peer leaders called camp health leaders are trained in communication skills and learn how to address myths and misconceptions concerning HIV transmission and prevention, condoms, violence and multiple sexual partnerships. Camp health leaders are asked to implement strategies among their peers and to keep a weekly diary documenting the number of HIV and gender-based violence conversations they had per week. | Sexually transmitted infection incidence  Intimate partner violence perpetration  Gender normsHope  Future orientation  Perceived social support  Social cohesion  Social ties  Unprotected sex  Sexual partner concurrency  Alcohol  Other substance use |
| 18 | Evaluating a multi-component, community-based program to improve adherence and retention in care among adolescents living with HIV in Zimbabwe: study protocol for a cluster randomized controlled trial [[20](#_ENREF_20)] [[21](#_ENREF_21)] | Zimbabwe | HIV-positive adolescents aged 13-19 years and eligible for antiretroviral therapy (ART) | Zvandiri intervention: Monthly support group and allocation to a designated community adolescent treatment supporter. Adolescents are followed up through SMS, calls and home visits. Participants in intervention and control arms receive ART and adherence support. | Mortality or viral load ≥ 1000 copies/ml at 96 weeks, assessed on a dried blood-spot sample  Retention in clinical services  Discontinuation of ART  Depression symptoms  Symptoms of common mental disorders  Quality of life |
| 19 | MAMAS: Mentoring Adolescent Mothers at School[[22](#_ENREF_22)] | South Africa | Girls aged 14-19 years who gave birth in the previous two weeks and have been enrolled in school in the previous year | Fifteen group sessions with a mentor mother and up to two home visits. Girls will also receive adolescent-friendly clinical care postnatally | Primary outcomes:  School enrolment  School absence  Frequency of sex  Condom use  Intimate partner violence  HIV, gonorrhoea, chlamydia and trichomonas vaginalis incidence  Secondary outcomes:  Peer support scale  Familial support scale  Application for child support grant  Received child support grant |
| 20 | Promoting adolescent engagement, knowledge and health evaluation of PAnKH: an adolescent girl intervention in Rajasthan, India[[23](#_ENREF_23)] | India | Girls aged 12-17 (unmarried) or 12-19 (married) | A rural community-based programme involving group education activities and weekly sports sessions. The education activities cover topics including gender norms, gender-based violence, sexual and reproductive health and education. They also aim to promote socio-emotional and non-cognitive skills and wellbeing. Some clusters will also receive community campaigns and events on these topics that are delivered at a community level. Activities are delivered by male and female mentors who are aged 18 to 24 years, reside in the study area, and have at least 12 years of schooling. | Primary outcomes:  Early marriage  Enrolment in school  Depression  Anxiety  Rumination  Socio-emotional and non-cognitive skills (e.g. self-efficacy, resilience and self-esteem)  Gender attitudes  Secondary outcomes:  Attitude towards school  Sexual and reproductive health knowledge  Attitudes and responses to violence  Attitude towards and participation in sports  Restrictions during menstruation |
| 21 | School-based programme for emotional and behavioural problems in adolescents[[24](#_ENREF_24)] | India | Boys and girls aged 11–18 years with a diagnosis of attention deficit hyperactivity disorder (ADHD), disruptive disorders, substance use disorder or mild to moderate depression or anxiety | Students, teachers, counsellors and nurses will be given training on the intervention and will provide collaborative care for adolescents with emotional and behavioural problems. | Primary outcomes:  Severity of ADHD, disruptive disorders, substance use disorder, anxiety and depression  Secondary outcomes:  School drop out rate  Assault or destructive behaviour in school  Academic performance  Self -harm |
| 22 | Participatory adolescent groups, youth leadership training and livelihood promotion to improve school attendance, dietary diversity and mental health among adolescent girls in rural eastern India [[25](#_ENREF_25)] | India | Eligibility to receive the intervention: Adolescent girls and boys aged 10-19 living in villages and adjoining hamlets located in rural areas of Jharkhand (eastern India)  Eligibility for the baseline and endline surveys: Girls aged 10-19 living in the study area and agree to be interviewed | Jharkhand Initiative for Adolescent Health (JIAH)  Three components: (i) peer facilitated participatory learning and action groups focusing on education, nutrition, health and violence; (ii) youth leadership activities to develop adolescents’ confidence and leadership skills; (iii) livelihood promotion activities (implemented in intervention and control areas) for adolescents and their families | **Primary outcome measure**  Assessed during the endline survey: Mean dietary diversity score among adolescent girls  Mean score on the Brief Problem Monitor – Youth % of adolescent girls attending school  **Secondary outcome measures**  Assessed during the endline survey: % of girls making decisions independently and with others about the food they eat including how much they eat and what types of food they eat  Mean score on gender role attitudes index % of girls making decisions independently and with others about friends, spending money and purchases Mean score on the Schwarzer General Self-Efficacy Scale Mean score on the Child and Youth Resilience Measure 11-item version (CYRM-Brief)  % of girls who report experiencing emotional violence in the past 12 months  % of girls who report experiencing physical violence in the past 12 months % of girls who report intervening to reduce emotional violence against their peers in the past 12 months  % of girls who report intervening to reduce physical violence against their peers in the past 12 months % of girls who report being absent from school in the past two weeks  % of girls accessing at least one school-related entitlement (cash, bicycles, books, midday meal scheme)  % of girls who drank alcohol in the past month |

**References**

1. Nct, Imperial College London, Biomedical Research and Training Institute (2006) Scientific Evaluation of Peer Education and STD Treatment to Reduce the Spread of HIV in Zimbabwe.

2. Isrctn, National Autonomous University of Mexico (2012) Impact on student health status of a training program of university student as health promoters.

3. Mendoza-Núñez VM, Mecalco-Herrera C, Ortega-Ávila C, Mecalco-Herrera L, Soto-Espinosa JL, et al. (2013) A randomized control trial: training program of university students as health promoters. pp. 162.

4. Actrn, Burnet Institute, Wan Smolbag Theatre (2013) Preventing early and unintended pregnancy in Vanuatu: a community-based intervention targeting adolescents.

5. Nct, Columbia University, Eunice Kennedy Shriver National Institute of Child Health and Human Development (2013) Evaluating a Youth-Focused Economic Empowerment Approach to HIV Treatment Adherence.

6. Nct, University of North Carolina CH, National Institute of Mental Health (NIMH), Muhimbili University of Health and Allied Sciences, American University (2013) Microfinance and Health Intervention Trial for Youth in Dar es Salaam, Tanzania.

7. Nct, Karnataka Health Promotion Trust, University of Manitoba, London School of Hygiene and Tropical Medicine (2013) Evaluation of an Intervention for Adolescent Girls in Karnataka.

8. Beattie TS, Bhattacharjee P, Isac S, Davey C, Javalkar P, et al. (2015) Supporting adolescent girls to stay in school, reduce child marriage and reduce entry into sex work as HIV risk prevention in north Karnataka, India: protocol for a cluster randomised controlled trial. pp. 292.

9. Aarø LE, Mathews C, Kaaya S, Katahoire AR, Onya H, et al. (2014) Promoting sexual and reproductive health among adolescents in southern and eastern Africa (PREPARE): project design and conceptual framework. pp. 54.

10. Irct201311124076N, Research Deputy Tehran University of Medical Sciences (2014) Efficacy of peer education on HIV-related high risk behaviors.

11. Nct, Hektoen Institute for Medical Research, Development EKSNIoCHaHa (2015) Improving Adherence Among HIV+ Rwandan Youth: A TI-CBTe Indigenous Leader Model.

12. Isrctn, Ministry of Health Malaysia (2016) Development and evaluation of a school-based smoking prevention intervention for 13-14 year old adolescents in Malaysia.

13. Nct, Stanford University, African Institute for Health and Development Kenya, Medical Research Council South Africa (2016) A Cluster-randomized Trial to Assess a Sexual Assault Prevention Intervention in Adolescents in Nairobi, Kenya.

14. Falb KL, Tanner S, Ward L, Erksine D, Noble E, et al. (2017) Creating opportunities through mentorship, parental involvement, and safe spaces (COMPASS) program: multi-country study protocol to protect girls from violence in humanitarian settings. BMC public health. pp. 231.

15. NCT02833441 (2016) The Peer Support Intervention - Supporting HIV Positive Adolescents in Zimbabwe to Improve HIV Care Continuum Outcomes.

16. NCT02882607 (2016) Peer Groups for Healthy Pregnancy & HIV Prevention for Young Malawian Women.

17. PACTR201612001889209 (2016) An evaluation of the effectiveness of peer-education in preventing new HIV infections among adolescents in Northern Malawi.

18. Sandoy IF, Mudenda M, Zulu J, Munsaka E, Blystad A, et al. (2017) Effectiveness of a girls' empowerment programme on early childbearing, marriage and school dropout among adolescent girls in rural Zambia: study protocol for a cluster randomized trial. Trials.

19. Kajula L, Balvanz P, Kilonzo MN, Mwikoko G, Yamanis T, et al. (2016) Vijana Vijiweni II: a cluster-randomized trial to evaluate the efficacy of a microfinance and peer health leadership intervention for HIV and intimate partner violence prevention among social networks of young men in Dar es Salaam. BMC Public Health 16: 113.

20. Mavhu W, Willis N, Mufuka J, Mangenah C, Mvududu K, et al. (2017) Evaluating a multi-component, community-based program to improve adherence and retention in care among adolescents living with HIV in Zimbabwe: study protocol for a cluster randomized controlled trial. Trials 18.

21. PACTR201711002755428 (2017) The impact of Community Adolescent Treatment Supporters on linkage, retention, adherence and psychosocial wellbeing among adolescents with HIV.

22. NCT03090802 (2017) MAMAS: Mentoring Adolescent Mothers at School.

23. ISRCTN77449378 (2018) Promoting adolescent engagement, knowledge and health evaluation of PAnKH: an adolescent girl intervention in Rajasthan, India.

24. CTRI/2018/01/011190 (2018) School-based Programme for Emotional and Behavioural problems in adolescents.

25. Prost A (2018) ISRCTN17206016: Participatory adolescent groups, youth leadership training and livelihood promotion to improve school attendance, dietary diversity and mental health among adolescent girls in rural eastern India.
