# Supplementary material for: Peer-facilitated community-based interventions for adolescent health in low- and middle-income countries: A systematic review
Source: PLoS One. 2019 Jan 23;14(1):e0210468. doi: 10.1371/journal.pone.0210468 (PMC6343892; doi:10.1371/journal.pone.0210468)
Supplement: S2 Table — (DOCX) [file pone.0210468.s004.docx]

**S2 Table: Characteristics of studies of community-based peer-facilitated interventions for adolescent health**

| **First author and year of publication of main trial paper** | **Design (total number of clusters, number of participants per cluster)** | **Country** | **Rural/urban** | **Setting** | **Area of adolescent health need** | **Primary and secondary outcomes** | **Intervention** | **Control** | **Baseline n (intervention, control)** | **Females (%)** | **Mean age (Years), (SD; Range)** |
| --- | --- | --- | --- | --- | --- | --- | --- | --- | --- | --- | --- |
| **Al-Sheyab 2012 [**[**31**](#_ENREF_31)**]** | cRCT (4, ?) | Jordan | Urban | School | Physical disorders | Primary outcomes: pediatric asthmatic quality of life, self-efficacy to resist smoking, knowledge of self-management of asthma | Triple A Program: a peer -led educational intervention on asthma self-management | Usual care | 261 (132, 129) | Intervention 36.4; Control 50.4 | Unclear. Year 8 to Year 10 students |
| **Ayaz 2015 [**[**32**](#_ENREF_32)**]** | cRCT (2, 297 in intervention and 110 in control) | Turkey | Urban | School | Substance abuse disorders | Smoking after peer education; knowledge about the dangers of smoking | Peer education for school-aged children on the dangers of smoking | Usual care (teacher led sessions on smoking and its dangers) | 407 (297, 110) | Intervention 53.0; Control 56.4 | Intervention 11.4 (1.7, ~8-13); Control 11.6 (1.8, ?) |
| **Balaji 2010 [**[**36**](#_ENREF_36)**]** | cRCT (4, ?) | India | Rural and Urban | School and community | Sexual and reproductive health/Violence/Mental disorders/Substance abuse disorders | Substance abuse, suicidal behaviour, probable depression (GHQ-12), experience of physical abuse, experience of sexual abuse, perpetration of physical violence, complaints of penile discharge, complaints of vaginal symptoms, complaints of menstrual problems, help seeking for RSH complaints by men, help seeking for RSH complaints by women, knowledge and attitudes (emotional health), knowledge and attitudes (substance use), knowledge and attitudes (RSH) | Yuva Mitr (Friend of youth in Konkani): a multicomponent population-based intervention to promote youth health involving peer education and peer led community based activities, teacher training (urban communities only) and dissemination of health information materials. | Waitlist | 3663 (?, ?) | Rural intervention 46.4, control 52.2; Urban intervention 49.9, control 56.2 | Rural 19.8 (?, 16-24); Urban 19.2 (?, 16-24) |
| **Carlson 2012 [**[**37**](#_ENREF_37)**,**[**55**](#_ENREF_55)**,**[**56**](#_ENREF_56)**]** | cRCT (30, 24) | Tanzania | Urban | School and community | Reduction in educational and employment marginalisation | Deliberative efficacy, communicative efficacy, academic efficacy, emotional control, peer resistance, child collective efficacy, neighbourhood-level collective efficacy, neighbourhood problems | Young Citizens Program: a curriculum-based intervention to develop young people's individual and collective efficacy to dramatize science and social context around HIV, HIV stigma, testing and treatment. | Waitlist | 724 (364, 360) | ? | ? (?, 9-14) |
| **Chen 2014 [**[**33**](#_ENREF_33)**]** | cRCT (4, ?)f | China | Urban | School | Substance abuse disorders | Primary outcomes: smoking-related knowledge, attitudes, ever smoking, daily smoking, weekly smoking and current smoking | Multicomponent school-based smoking intervention focusing on health policies, the school environment and personal health skills | Usual care | 1807 (941, 866) | Linzhi Tibetan intervention 55.6, control 58.1; Guangzhou Han intervention 45.0, control 48.0 | 14.5 (1.1, ?) |
| **Church 2012 [**[**46**](#_ENREF_46)**,**[**57**](#_ENREF_57)**]** | RCT | Philippines | Urban | College | Mental disorders | Depression (BDI) | Emotional freedom psychotherapy techniques | Usual care | 30 (15, 15) | Total 83.3^a^ | 16.7 (?, 16-18) |
| **Cowan 2010 [**[**38**](#_ENREF_38)**,**[**58**](#_ENREF_58)**]** | cRCT (30, ?) | Zimbabwe | Rural | School, health facilities and community | HIV & AIDS/Sexual and reproductive health | Primary outcomes: prevalence of HIV, prevalence of HSV-2,  Secondary outcomes: reported symptoms of STDs, pregnancy prevalence, knowledge related to STD acquisition, pregnancy prevention and HIV acquisition, self-efficacy related to condoms, sexual refusal and HIV testing, attitudes related to control over sex, gender empowerment, control over life and future, reported sexual behaviour (ever had sex, sexual debut 17 or younger, two or more lifetime partners, condom use), reported pregnancy prevention, clinic attendance and perceptions of staff | Regai Dzive Shiri Intervention: a multicomponent youth programme for in and out of school youths, their parents, and community stakeholders. | Waitlist | 6791 (3381, 3410) | Intervention 47.9; control 52.1^a^ | ?, (?, 18-22) |
| **Decat 2015 [**[**44**](#_ENREF_44)**,**[**59**](#_ENREF_59)**,**[**60**](#_ENREF_60)**]** | cRCT (18, ?) | Nicaragua (also Bolivia and Ecuador, however an RCT was not used in these two countries)^c^ | Urban | Health facilities, community (door to door outreach, mobile) cinemas, campaigns) | Sexual and reproductive health | Communication on sex, use and knowledge of health services, improved condom use | Community embedded reproductive health care for adolescents led by 'Friends of Youths'. The intervention involves family talks, mobile cinema, distributing educational materials for parents of adolescents, work with community leaders to provide opportunities for adolescents, social media campaigns and resources, awareness raising/capacity building with health facilities, outreach to vulnerable adolescents to encourage them to go to healthcare centres, work with the Ministry of Health | Usual care | 2766 (?, ?) | Total 53.5 | 15.2 (?,13-19) |
| **Devries 2015 [**[**41**](#_ENREF_41)**,**[**48**](#_ENREF_48)**,**[**61**](#_ENREF_61)**]** | cRCT (42, ≤130) | Uganda | Rural and Urban | School | Violence/Mental disorders | Primary outcomes: past week physical violence by school staff (self-reported by students and school staff)  Secondary outcomes: educational achievement, mental health status (SDQ), safety and wellbeing in school, past term physical violence by school staff (self-reported by students) | Good School Toolkit: a school-based multicomponent intervention to reduce violence against students by school staff involving behaviour change techniques that engage students, school staff and parents, including setting school-wide goals, developing action plans, reflection on experiences of violence to encourage empathy, and improving knowledge on positive forms of discipline. Schools work through six steps in the Toolkit manual. The programme is led by a small team of staff and student 'protagonists'. | Waitlist | 3706 (1824, 1882) | Intervention 50.8; control 53.7 | 13.0 (1.5, ~11-14) |
| **Harrell 2016 [**[**42**](#_ENREF_42)**,**[**62**](#_ENREF_62)**]** | cRCT (14, ?) | India | Urban | Community | Substance abuse disorder | Current tobacco use (defined as smoking, chewing or using other smokeless products in the past 30 days), current smokeless tobacco use, intention to smoke, intention to use smokeless tobacco, susceptibility to smoke, susceptibility to use smokeless tobacco, psychosocial and environmental risk factors for tobacco use (e.g. knowledge, normative beliefs, perceptions related to tobacco use) | Project ACTIVITY: a multicomponent intervention involving training of NGO workers, youth and adult leaders to deliver the intervention; peer-led interactive activities with support groups and outreach; tobacco cessation camps; enforcement of India's tobacco control law in local communities | Usual care plus free eye and dental care | 6023 (3034, 2989) | Intervention 48.62; Control 47.78) | 52% 10-14 years; 48% 15-19 years |
| **Jewkes 2008 [**[**39**](#_ENREF_39)**,**[**63**](#_ENREF_63)**]** | cRCT (70, 40) | South Africa | Rural | Community | HIV & AIDS/Sexual and reproductive health/Violence/Mental disorders/Substance abuse disorders | Incidence of HIV, incidence of HSV-2, number of partners, any transactional sex with casual partner, >1 incident of physical or sexual intimate partner violence, rape or attempted rape (men only), correct condom use at last sex, any casual partner, unwanted pregnancy, depression (CES-D), problem drinking, ever misused drugs | Stepping Stones: Participatory learning approach to build knowledge, risk awareness and communication skills and to stimulate critical reflection. It uses roleplay and drama, drawing on the reality of participants’ lives. 13 group sessions are delivered to single sex groups, plus there are 3 meetings for males and females, and a final community meeting. Group sessions covered topics such as sex and love, conception and contraception, unwanted pregnancy and sexually transmitted diseases and HIV. | A single three-hour session on HIV, safer sex, and condoms. The content was taken from Stepping Stones. | 2776 (1367, 1409) | Intervention 50.7; control 51.3 | ? (?. 15-26) |
| **Lotrean 2010 [**[**34**](#_ENREF_34)**]** | cRCT (20, ?) | Romania | Urban | School | Substance abuse disorders | Risk of non-smokers becoming regular smokers, attitudes, self-efficacy and intentions related to smoking | A peer-led school based smoking prevention program | ? | 1196 (?, ?) | Intervention 50.9; control 51.5 | 13.7 (0.3, 13-14) |
| **Mmbaga 2017 [**[**35**](#_ENREF_35)**]** | cRCT  (38, 153) | Tanzania | Urban/peri-urban | School | Sexual and reproductive health | Condom use | PREPARE intervention involving a peer, teacher and health care provider implemented components | Waitlist | 5091 (2503, 2588) | Intervention 50.9; control 50.3 | ? (?, 12-14) |
| **Okonofua 2003 [**[**45**](#_ENREF_45)**]** | cRCT (12, 160)^g^ | Nigeria | Urban | School, health facilities and community | Sexual and reproductive health | Knowledge of STDs, condom use, reported symptoms of and STD, ever had sex, place of STD treatment | Multicomponent adolescent sexual and reproductive health intervention involving: health clubs in schools to raise awareness and discuss reproductive health; training of peer educators on STD prevention and treatment to provide one-to-one or group counselling to other students, to distribute educational materials, and to refer adolescents with STD symptoms to health providers; and training health providers | Usual care | 1896 (643, 649, 604)^b^ | Intervention 52.5; Benin control 50.7; Ekpoma control 56.6 | Intervention 17.4 (?, 14-18); Benin control 18.2 (?, 14-18); Ekpoma control 18.5 (?, 14-18) |
| **Perry 2009 [**[**47**](#_ENREF_47)**,**[**64**](#_ENREF_64)**,**[**65**](#_ENREF_65)**]** | cRCT (32, ?) | India | Urban | School | Substance abuse disorders | Chewing tobacco use, bidi smoking, cigarette smoking, any tobacco use, intention to use chewing tobacco, intention to smoke cigarettes, knowledge, beliefs, refusal skills, perceptions, normative beliefs, advocacy skills related to tobacco use, support of tobacco control policies, reasons not to use tobacco, social susceptibility to chewing tobacco or cigarette smoking | Project MYTRI: a multicomponent school-based intervention with peer led health activism, school posters and postcards for parents | Waitlist | 11748 (?,?) | 48.4^e^ | Mean ages: 11 years in grade 6; 12.1 in Grade 7; 12.8 in Grade 8; 13.9 in Grade 9 |
| **Ross 2007 [**[**43**](#_ENREF_43)**,**[**66-70**](#_ENREF_66)**]** | cRCT (20, ?) | Tanzania | Rural | School, communities, health facilities | HIV & AIDS/Sexual and reproductive health | Primary outcomes: HIV seroincidence, HSV-2 seroprevalence  Secondary outcomes: knowledge on HIV acquisition, STD acquisition and pregnancy prevention, reported attitudes to sex, reported sexual behaviour ( sexual debut, more than one partner in past 12 months, first used condom, used condom at last sex, went to health facility for most recent STD symptoms, syphilis prevalence, chlamydia prevalence, gonorrhoea prevalence, trichomonas prevalence, pregnancy prevalence, reported pregnancy | MEMA kwa Vijana Project: a multicomponent intervention involving reproductive health education in primary schools, training of health workers to provide empathic and confidential health services, community condom provision and promotion by youth, community activities, including youth health weeks, quarterly screening of videos and interschool competitions and performances to enhance the acceptability and reach of interventions. | Usual care plus family planning services and improved case management of STD in control and intervention | 9645 (4870, 4775) | Intervention 44.4; Control 44.9 | ? (?, 14-18+) |
| **Sherman 2009 [**[**19**](#_ENREF_19)**,**[**53**](#_ENREF_53)**]** | RCT | Thailand | Rural and Urban | Community | HIV & AIDS/Sexual and reproductive health/mental health/substance abuse disorders | Methamphetamine use in the past three months, condom use, incidence of HIV, chlamydia, gonorrhoea, HSV-2, HCV, depression (CES-D) | Peer educator network orientated intervention involving peer facilitators training current methamphetamine users ('index participants') in communication techniques which they can use to share messages about the risks of drug use with members of their social network. | Thai best practice "life skills" curriculum including discussions on problem-solving, decision-making skills, MA use and STDs | 983 (495, 488) | Intervention 27.3; Control 27.2 | 19 (18-25)^f^ |
|  |  |  |  |  |  |  |  |  |  |  |  |
| **Singhal 2010 [**[**49**](#_ENREF_49)**]** | cRCT (2, c.100) | India | Urban | School, health facilities and community | Physical disorders | Height, weight, BMI, waist circumference, mid upper arm circumference, mid-thigh circumference, sagittal abdominal diameter, triceps and biceps skinfold thickness, waist to height ratio, waist to hip ratio, waist to thigh ratio, arm fat mass, arm muscle mass, leg fat mass, leg muscle mass, trunk fat mass, trunk muscle mass, fasting blood glucose, triglycerides, high density lipoprotein cholesterol, knowledge about nutrition, change in food intake, behaviour related to diet, change in lifestyle, change in attitude related to food, change in body image and self-esteem | Multicomponent school based programme for nutrition and health involving: dissemination of health-related information through lectures and focused group discussions; promotion of physical activity; diet planning and nutrition education through quizzes and planning activities; individual counselling with a trained nutritionist; policy changes in the school to change the canteen menu; health camp in school for parents and teachers; training student volunteers to disseminate health messages through skits, recipe demonstrations, counselling junior students on what foods to put in their tiffin | Usual care | 201 (99, 102) | Intervention 39.3; Control 40.2 | Intervention 16.04 (0.41, 15-17); Control 16.0 (0.5, 15-17) |
| **Ssewamala 2010 [**[**50**](#_ENREF_50)**,**[**71-75**](#_ENREF_71)**]** | cRCT (15, ?) | Uganda | Rural | School | Mental disorders/Sexual and reproductive heath/Physical disorders/Education and employment marginalisation | Sexual risk-taking intentions, mental health functioning (Tennessee Self-Concept Scale) depression (Children’s Depression Inventory), self-rated physical health, attitudes towards sexual risk-taking, average monthly net deposit, attitudes towards saving money, educational plans and aspirations (planning to go on to secondary school, planning to go to college or university, certainty to accomplish educational plans), academic performance (school grades, school attendance) | Suubi-program: a multicomponent intervention for AIDS orphaned children attending selected primary schools, involving workshops focused on asset-building and future planning, a monthly mentorship program for adolescents with peer mentors on life options; a Child Development Account (CDA) dedicated to paying for secondary schooling, vocational training and/or a family small business (CDAs were matched savings accounts with a match rate of 2:1) | Usual "reactive" care for orphaned children | 286 (138, 148) | Intervention 60.3; Control 53.3 | 13.7 (1.3, 10-14) |
| **Thurman 2016 [**[**40**](#_ENREF_40)**,**[**76**](#_ENREF_76)**]** | cRCT (57, ?) | South Africa | Rural | Community (home visits and drop in centres) | Sexual and reproductive health/mental disorders | Primary outcomes: sexual debut, consistent condom use (i.e. reported use of a condom at every episode of sexual intercourse in the prior six months)  Secondary outcomes: engagement in risky sexual partnerships, depression symptomatology (CES-DC) | Two interventions: (i) interpersonal psychotherapy for groups; (ii) 'Vhutshilo', a curriculum-based behavioural intervention led by peer facilitators | Usual care | 1014 (Vhutshilo 243, IPTG 260, Vhutshilo & IPTG 282, Control 229) | Vhutshilo 47.8; IPTG 52.0; Vhutshilo & IPTG 46.1; Control 48.0 | 15.5 (1.05 ^d^, 14-17) |

cRCT cluster Randomised Controlled Trial; RCT Randomised Controlled Trial; ? unknown; BDI Beck Depression Inventory; SDQ Strengths and Difficulties Questionnaire; CES-D Center for Epidemiologic Studies Depression Scale; CES-DC Centre for Epidemiologic Studies Depression Scale for Children; MA methamphetamine use; RSH Reproductive and Sexual Health; GHQ-12 General Health Questionnaire (12 item); STD Sexually Transmitted Disease

^a^ Percentage of females at follow-up; ^b^ Number of participants in intervention arm plus Benin control arm plus Ekpoma control arm; ^c^ This study was conducted in three Latin American countries - Bolivia, Ecuador and Nicaragua - however we only present data from Nicaragua because an RCT design was not used in the other countries; ^d^ standard error; ^e^ Percentage of females who completed one or more surveys; ^f^ median (IQR); ^g^ This study involved two control groups: (i) randomly selected control schools in the same city (Benin) as the intervention schools; and (ii) randomly selected schools from a nearby town (Ekpoma) allocated to the control intervention based on location –we only included outcomes comparing the intervention arm to (i), as (ii) is not compatible with an RCT design.
